# The safety of combining immune checkpoint inhibitors and platinum-based chemotherapy for the treatment of solid tumors: A systematic review and network meta-analysis

**DOI:** 10.3389/fimmu.2023.1062679

**Published:** 2023-02-07

**Authors:** Ting Mei, Ting Wang, Qianyue Deng, Youling Gong

**Affiliations:** ^1^ Department of Thoracic Oncology, Cancer Center and State Key Laboratory of Biotherapy, West China Hospital, Sichuan University, Chengdu, China; ^2^ Lung Cancer Center, West China Hospital, Sichuan University, Chengdu, China

**Keywords:** immune checkpoint inhibitors, safety, chemotherapy, adverse events, network comparison

## Abstract

**Objective:**

Combination treatment regimens consisting of both immune checkpoint inhibitors (ICI) and chemotherapeutic agents have emerged as the standard of care for a range of cancers. This network meta-analysis (NMA) examined the toxicity profiles and safety rankings of these different ICI-based combination regimens.

**Methods:**

The PubMed, EMBASE, Web of Science, and Cochrane Library databases were searched for all randomized controlled trials (RCTs) published as of March 1, 2022 comparing two or more treatment regimens in which at least one arm was comprised of an ICI + platinum-based chemotherapeutic regimen. Treatment-related adverse events (AEs) of any grade and AEs of grade 3 or higher were the primary endpoints for this analysis, while specific AE types were secondary endpoints. This NMA combined both direct and indirect comparisons when analyzing odds ratios (ORs) and the surface under the cumulative ranking curve (SUCRA) for different ICI-based treatment regimens.

**Results:**

In total, 33 RCTs enrolling 19,012 cancer patients were included in this NMA. Of the analyzed regimens, avelumab + chemotherapy and camrelizumab + chemotherapy were associated with a significantly greater risk of AEs of any grade relative to ipilimumab + chemotherapy, durvalumab + chemotherapy, or pembrolizumab + chemotherapy. No significant differences in the risk of AEs of grade 3 or higher were observed when comparing different ICI regimens. Hepatotoxicity and pyrexia were the most common AEs associated with atezolizumab + chemotherapy treatment. Ipilimumab + chemotherapy was associated with a relatively higher risk of gastrointestinal and skin toxicity. Skin toxicity and hypothyroidism were the major AEs associated with nivolumab + chemotherapy. Fatigue and pneumonia were the most common AEs respectively associated with sugemalimab + chemotherapy and pembrolizumab + chemotherapy regimens.

**Conclusions:**

Of the evaluated regimens, camrelizumab + chemotherapy and avelumab + chemotherapy were associated with significantly higher rates of AEs of any grade, whereas durvalumab and sintilimab were relatively safe PD-L1 and PD-1 inhibitors, respectively, when administered in combination with platinum-based chemotherapy. However, none of the evaluated ICI + chemotherapy regimens exhibited any differences with respect to the incidence of grade 3 or higher AEs, offering guidance that may be of value in routine clinical practice.

## Introduction

Immune checkpoint inhibitors (ICIs), particularly antibodies targeting programmed cell death 1 (PD-1), programmed death ligand 1 (PD-L1), and anti-cytotoxic T-lymphocyte antigen 4 (CTLA-4), have revolutionized the field of Immuno-oncology and emerged as the standard of care for first- or second-line treatment of a range of cancer types (1, 2). Clinical data derived from several phase III randomized controlled trials (RCTs) has revealed that combining ICIs with platinum-based chemotherapy can improve the overall survival and objective response rates for several cancers (3). It is worth noting that some cancer patients may be combined with BRCA1/2 mutations, resulting in the disruption of the high-fidelity homologous recombination DNA repair pathway, so these patients are very sensitive to poly ADP ribose polymerase (PARP) inhibitors. In addition, several studies have shown that BRCA1/2 mutations harbor more neoantigens, harbor more tumor-infiltrating lymphocytes, and increase PD-L1 expression. Therefore, for patients with BRCA1/2 mutations, combined use of PARP inhibitors and PD-1 inhibitors may enhance antitumor responses (4).

Chemotherapeutic agents exhibit exhaustively studied toxicity profiles that can be managed with appropriate clinical experience (5, 6). Immune-related AEs (irAEs) are unintended side effects that result from the activation of the immune system in response to ICI treatment, and can impact any organ including the lungs, endocrine system, heart, kidneys, muscle, liver, or skin (7). A network meta-analysis (NMA) conducted by Geisler et al. revealed that combining immunotherapy and chemotherapy was associated with an increased risk of grade 3 or higher AEs as compared to ICI monotherapy (8), whereas Chen et al. conversely found that combining chemotherapy and ICI regimens resulted in a significant reduction in treatment-related AE incidence as compared to ICI monotherapy (9). The impact of combining chemotherapy and ICIs on AE incidence may be complex and difficult to predict. When AEs are not detected and treated in a timely manner, they can result in severe complications resulting in interrupted treatment, treatment failure, or death.

Prior NMAs have explored AEs associated with different ICIs, ICIs + chemotherapy relative to chemotherapy alone, single-agent ICIs relative to two-agent ICIs, and PD-1 versus PD-L1 ICIs (9–12). Given that ICI + platinum-based chemotherapy is the standard of care for many cancers, examining the safety profiles associated with different ICI + chemotherapy regimens can provide information that can guide clinical decision-making efforts. However, practical limitations and study designs can make adequately understanding AE profiles resulting from different ICI + chemotherapy regimens in different RCTs challenging. As such, further work is needed to establish which ICIs exhibit the best safety profile when combined with platinum-based chemotherapeutic agents.

NMA approaches offer an opportunity to compare a range of treatments based on a combination of indirect and direct evidence, allowing for the ranking of different therapeutic regimens. As such, the present study was designed as an NMA exploring the relative safety and toxicity profiles of different ICI + chemotherapy regimens.

## Methods

This NMA was conducted based on a protocol established in advance on the PROSPERO platform (PROSPERO: CRD42022315954; https://www.crd.york.ac.uk/PROSPERO/#searchadvanced), and was performed in a manner consistent with the PRISMA (Preferred Reporting Items for Systematic Reviews and Meta-analyses) guidelines and the NMA extension of these guidelines (**eTable 1**).

## Data sources and searches

Those RCTs including at least one arm consisting of ICI + platinum-based chemotherapy treatment regimens published in English as of March 2022 were identified by searching the PubMed, Embase, Cochrane Library, and Web of Science databases. For full details regarding the terms used for this search strategy, see **eTable 2**. The titles, abstracts, and full text of included studies were independently screened by two investigators (MT and GYL) to determine whether studies were eligible for inclusion. Any discrepancies were resolved through discussion with a third investigator (DQY).

### Study selection

Phase II and Phase III RCTs comparing two or more therapeutic regimens, including at least one ICI + platinum-based chemotherapy regimen, were included in this analysis. Studies were excluded if they were derived from posters, conference abstracts, or reports pertaining to currently ongoing RCTs. When multiple studies were derived from a single trial, only one study was analyzed. Studies were excluded if they did not report on safety analyses, included targeted therapies, including bevacizumab, or utilized chemotherapy regimens that were not platinum-based (carboplatin or cisplatin).

### Study outcomes and data extraction

The primary outcome indicators for this NMA were treatment-related AEs of any grade and AEs of grade 3 or higher. When studies did not report treatment-related AEs, AEs of any cause were instead evaluated. Secondary endpoints for this analysis included specific types of AEs (of any grade or grade 3+). Pre-designed tables were independently used by two investigators (MT and GYL) to extract data including study ID, first author, year of publication, study design, arms, treatment regimens, total patient number, number of patients included in safety analyses, and follow-up duration.

### Quality assessment

The Cochrane Risk of Bias Assessment Tool was used by two investigators to evaluate the quality of included RCTs based on seven potential sources of bias, including random sequence generation, allocation concealment, blinding of participants and personnel, outcome assessment blinding, incomplete outcome data, selective reporting, and other biases. The risk of bias for each category was classified as low (green), medium (yellow), or high (red).

### Data synthesis and analysis

STATA 16.0 was used to generate a network geometry map outlining direct and indirect comparisons between treatment strategies. Odds ratios and 95% confidence intervals were used to examine the impact of individual treatment regimens on the risk of any AEs or AEs of grade 3 or higher. The I^2^ statistic was used to quantify heterogeneity, with I^2^ < 25%, 25% - 50%, and > 50% respectively corresponding to low, medium, and high degrees of heterogeneity, with random effects models being employed when I^2^ > 50% (13). Global inconsistency in the overall network model was analyzed through the statistical global inconsistency test. Differences between indirect and direct comparisons in the closed treatment loop were explored *via* a node-splitting analysis. *p* < 0.05 was considered indicative of significant inconsistency. Random effects and consistency models were constructed with the Markov chain Monte Carlo (MCMC) method from the “JAGS” (v 4.3.0) and “getmtc” (v 0.8.2) packages in R (v 4.0.2). Four independent Markov chains were simultaneously run for each outcome measurement for 5,000 burn-ins and 20,000 inference iterations per chain to generate the posterior distribution. Model convergence was analyzed with trace plots and the Brooks-Gelman-Rubin method (14). A Bayesian approach was used to calculate the surface under the cumulative ranking curves (SUCRA) for each therapeutic regimen, with larger SUCRA values corresponding to better safety profiles for a given regimen (15). Publication bias was analyzed using funnel plots, with symmetrical funnel plot distributions being suggestive of an absence of publication bias (16). Sensitivity analyses were used to confirm the robustness of these results by specifically focusing on phase III RCTs or studies enrolling lung cancer patients.

## Results

### Study selection and characteristics

**Figure 1:**
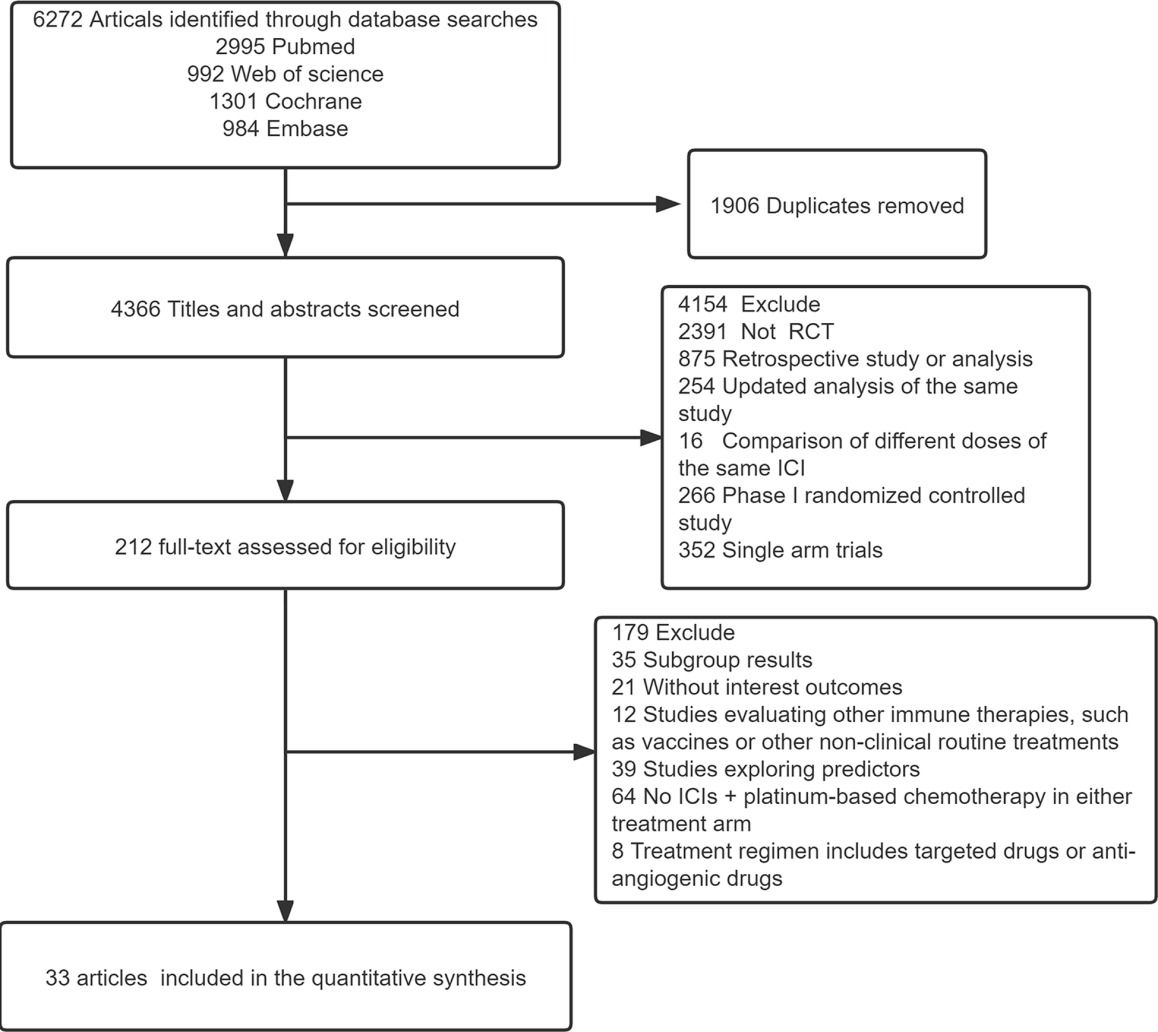
Flow diagram for the study selection process. ICI, immune checkpoint inhibitor; RCT, Randomized controlled trial.

**Figure 2:**
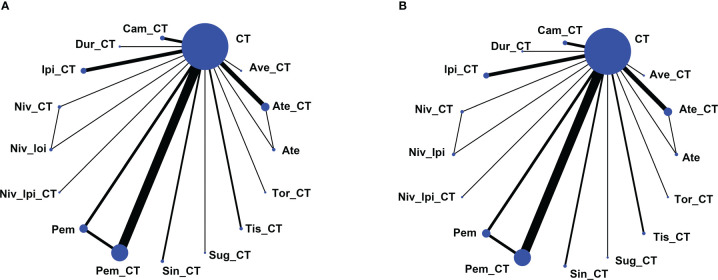
Network map of comparisons based on 16 therapeutic regimens for adverse events of any grade **(A)** or adverse events of grade 3 or higher **(B)**. Treatment types are represented by circular nodes, and node size is proportional to the number of patients administered a given treatment. Lines represent head-to-head comparisons, and line width is proportional to the total number of studies comparing the connected treatments. Ate, Atezolizumab; CT, Chemotherapy; Ate_CT, Atezolizumab + chemotherapy; Ave_CT, Avelumab + chemotherapy; Cam_CT, Camrelizumab + chemotherapy; Dur_CT, Durvalumab + chemotherapy; Ipi_CT, Ipilimumab + chemotherapy; Niv_CT, Nivolumab + chemotherapy; Niv_Ipi, Nivolumab + Ipilimumab; Niv_Ipi_CT, Nivolumab + Ipilimumab + chemotherapy; Pem, Pembrolizumab; Pem_CT, Pembrolizumab + chemotherapy; Sin_CT, Sintilimab + chemotherapy; Sug_CT, Sugemalimab + chemotherapy; Tis_CT, Tislelizumab + chemotherapy; Tor_CT, Toripalimab + chemotherapy.

Using the search strategy outlined in **Figure 1**, 6,272 studies of potential relevance were initially identified, with 33 RCT studies (3 phase II RCTs and 30 phase III RCTs) evaluating 16 treatment options ultimately being incorporated into this meta-analysis, as detailed in **Table 1** (17–49). **Figure 2** presents the outcome comparisons for this network meta-analysis, including adverse events of any grade and events of grade 3 or higher, with specific adverse event types being detailed in **eFigure 1**.

**Table 1:** Baseline characteristics of 33 studies.

Study ID	Year	First author	Trial phase	Total number	Safety analysis number	Arm	Treatment	Median follow-up time
IMvigor130	2020	Matthew DGalsky	III	451	453	1	Atezolizumab 1200mg + platinum-based chemotherapy every 3 weeks	11.8
				362	354	2	Atezolizumab 1200 mg every 3 weeks	
				400	390	3	Platinum-based chemotherapy	
KEYNOTE-361	2021	ThomasPowles	III	351	349	1	Pembrolizumab 200 mg + platinum-based chemotherapy every 3 weeks	31.7
				307	302	2	Pembrolizumab 200 mg every 3 weeks	
				352	342	3	Gemcitabine plus investigator’s choice of cisplatin or carboplatin	
CAPTAIN-1st	2021	Yunpeng Yang	III	134	134	1	Camrelizumab 200 mg + gemcitabine and cisplatin every 3 week	10.2
				129	129	2	Gemcitabine and cisplatin every 3 week	
JUPITER-02	2021	Hai-Qiang Mai	III	146	146	1	Toripalimab and gemcitabine and cisplatin once every 3 weeks	NR
				143	143	2	Gemcitabine and cisplatin once every 3 weeks	
KEYNOTE-048	2019	BarbaraBurtness	III	301	300	1	Pembrolizumab 200 mg every 3 weeks	11.5
				281	276	2	Pembrolizumab 200 mg plus a platinum and 5-fluorouracil every 3 weeks	13
				300	287	3	Cetuximab 400 mg/m2 loading dose, then 250 mg/m2 per week plus a platinum and 5-fluorouracil	10.7
ESCORT-1st	2021	Huiyan Luo	III	298	298	1	Camrelizumab 200mg plus paclitaxel and cisplatin every 3 weeks	10.8
				298	297	2	Paclitaxel (175 mg/m2) and cisplatin (75 mg/m2)	
KEYNOTE-062	2022	Kohei Shitara	III	256	254	1	Pembrolizumab 200 mg every 3 weeks	29.4
				257	250	2	Pembrolizumab plus cisplatin + fluorouracil or capecitabine every 3 weeks	
				250	244	3	Cisplatin + fluorouracil or capecitabine every 3 weeks	
KEYNOTE-590	2021	Jong-Mu Sun	III	373	370	1	Pembrolizumab 200 mg plus 5-fluorouracil and cisplatin once every 3 weeks	22.6
				376	370	2	5-fluorouracil and cisplatin once every 3 weeks	
CheckMate 648	2022	Yuichiro Doki	III	321	310	1	Nivolumab 240 mg every 2 weeks plus fluorouracil and cisplatin	NR
				324	322	2	Nivolumab (3 mg/kg every 2 weeks) plus ipilimumab (1 mg/kg every 6 weeks)	
				304	325	3	Fluorouracil and cisplatin every 3 weeks	
GEMSTONE-302	2022	Caicun Zhou	III	320	320	1	Sugemalimab 1200mg every 3 weeks plus platinum-based chemotherapy	17.8
				159	159	2	Platinum-based chemotherapy	
IMpower131	2020	RobertJotte	III	338	332	1	Atezolizumab 1200 mg plus carboplatin + paclitaxel	NR
				343	334	2	Atezolizumab 1200 mg plus carboplatin + nab-paclitaxel	26.8
				340	334	3	Carboplatin+nab-paclitaxel	24.8
IMpower130	2019	HowardWest	III	483	473	1	Atezolizumab 1200 mg every 3 weeks plus chemotherapy (carboplatin plus nab-paclitaxel)	18.5
				240	232	2	Chemotherapy (carboplatin plus nab-paclitaxel)	19.2
KEYNOTE-189	2018	Leena Gandhi	III	410	405	1	Pembrolizumab 200 mg plus chemotherapy (pemetrexed and platinum-based drug)	10.5
				266	202	2	Chemotherapy (pemetrexed and platinum-based drug)	
KEYNOTE-407	2018	Luis Paz-Ares	III	278	278	1	Pembrolizumab 200 mg plus carboplatin and either paclitaxel or [nab]–paclitaxel	7.8
				281	280	2	Carboplatin and either paclitaxel or [nab]–paclitaxel	
KEYNOTE-021	2022	Corey J Langer	II	60	59	1	pembrolizumab 200 mg plus carboplatin and pemetrexed every 3 weeks	10.6
				63	62	2	Carboplatin and pemetrexed every 3 weeks	
RATIONALE 304	2021	Shun Lu	III	223	222	1	Tislelizumab plus platinum (carboplatin or cisplatin) and pemetrexed every 3 weeks	9.8
				111	110	2	Platinum (carboplatin or cisplatin) and pemetrexed every 3 weeks	
NCT03594747	2021	Jie Wang	III	120	120	1	Tislelizumab 200 mg plus paclitaxel and carboplatin	8.6
				119	118	2	Tislelizumab plus nab-paclitaxel and carboplatin	
				121	117	3	Paclitaxel and carboplatin	
CASPIAN	2019	LuisPaz-Ares	III	268	265	1	Durvalumab 1500 mg plus platinum–etoposide every 3 weeks	14.2
				269	266	2	Platinum–etoposide every 3 weeks	
KEYNOTE-604	2020	Charles M. Rudin	III	228	223	1	Pembrolizumab 200 mg plus etoposide and platinum every 3 weeks	21.6
				225	223	2	Etoposide and platinum every 3 weeks	
IMpower133	2018	Leora Horn	III	201	198	1	Atezolizumab plus carboplatin and etoposide every 3 weeks	13.9
				202	196	2	Carboplatin and etoposide every 3 weeks	
CA184-156 study	2016	Martin Reck	III	478	478	1	Ipilimumab plus etoposide and platinum (cisplatin or carboplatin)	10.5
				476	476	2	Etoposide and platinum (cisplatin or carboplatin)	10.2
M.Reck 2013	2013	M.Reck	II	45	44	1	Paclitaxel + carboplatin every 3 weeks	11.1
				43	42	2	Concurrent-ipilimumab regimen (ipilimumab + paclitaxel + carboplatin)	
				42	42	3	Phased-ipilimumab regimen (paclitaxel + carboplatin followed ipilimumab)	
CheckMate 9LA	2021	LuisPaz-Ares	III	361	358	1	Nivolumab plus ipilimumab combined with platinum doublet chemotherapy	13.2
				358	349	2	Platinum doublet chemotherapy	
CameL	2021	Caicun Zhou	III	205	205	1	Camrelizumab plus carboplatin + pemetrexed every 3 weeks	11.9
				207	207	2	Carboplatin + pemetrexed every 3 weeks	
NCT03607539	2020	Yunpeng Yang	III	266	266	1	Sintilimab 200 mg plus pemetrexed and platinum once every 3 weeks	8.9
				131	131	2	Pemetrexed and platinum once every 3 weeks	
IMpower132(Japanese)	2021	Makoto Nishio	III	48	48	1	Atezolizumab 1200 mg plus pemetrexed and cisplatin or carboplatin	31.7
				53	52	2	Pemetrexed and cisplatin or carboplatin	29.3
IMpower132	2021	MakotoNishio	III	292	291	1	Atezolizumab 1200 mg plus pemetrexed and cisplatin or carboplatin	31.7
				286	274	2	Pemetrexed and cisplatin or carboplatin	29.3
NCT01285609	2017	Ramaswamy Govindan	III	388	388	1	Ipilimumab plus paclitaxel and carboplatin every 3 weeks	12.5
				361	361	2	Paclitaxel and carboplatin every 3 weeks	11.8
NCT00527735	2012	Thomas J. Lynch	II	66	65	1	Paclitaxel and carboplatin every 3 weeks	NR
				70	71	2	Concurrent-ipilimumab (ipilimumab plus paclitaxel and carboplatin)	
				68	67	3	Phased ipilimumab (paclitaxel and carboplatin followed by ipilimumab plus paclitaxel and carboplatin)	
ORIENT-12	2021	Caicun Zhou	III	179	179	1	Sintilimab 200 mg plus gemcitabine and cisplatin or carboplatin	12.9
				178	178	2	Gemcitabine and cisplatin or carboplatin	
JAVELIN Ovarian 100	2021	Bradley JMonk	III	332	328	1	Chemotherapy (carboplatin plus paclitaxel) followed by avelumab maintenance every 2 weeks)	12.6
				331	329	2	Chemotherapy plus avelumab every 3 weeks followed by avelumab maintenance	12.6
				335	334	3	Chemotherapy followed by observation	11.8
KEYNOTE-355	2020	JavierCortes	III	566	562	1	pembrolizumab 200 mg every 3 weeks plus chemotherapy (nab-paclitaxel; paclitaxel; or gemcitabine plus carboplatin)	25.9
				281	281	2	Chemotherapy (nab-paclitaxel; paclitaxel; or gemcitabine plus carboplatin)	26.3
KEYNOTE-522	2020	Peter Schmid	III	784	781	1	Pembrolizumab plus paclitaxel and carboplatin every 3 weeks + doxorubicin-cyclophosphamide or epirubicin-cyclophosphamide	15.5
				390	389	2	Paclitaxel and carboplatin every 3 weeks + doxorubicin- cyclophosphamide or epirubicin -cyclophosphamide	

NR, not report.

### Assessment of risk of bias and heterogeneity/inconsistency

**Figure 3:**
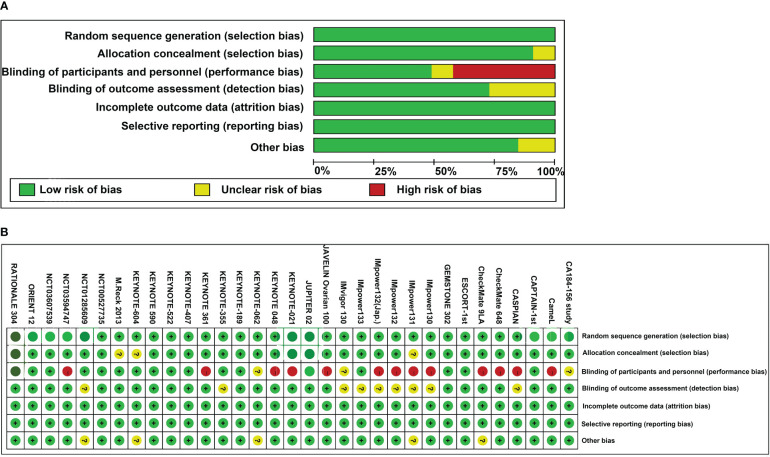
Risk bias assessment **(A)** Risk of bias graph; **(B)** Risk of bias summary.

The results of risk of bias analyses for these RCTs are provided in **Figure 3**. Of these trials, 14 exhibited a high risk of bias, 14 exhibited an unclear risk of bias, and the remaining studies exhibited a low risk of bias.

As significant heterogeneity was evident for some study outcomes (I^2^ > 50%), this network meta-analysis was performed with a random effects model (**eFigure 2**).

The global inconsistency test did not detect any significant inconsistency in this network meta-analysis (all *p*>0.05) (**eFigure 3**), nor did node splitting analyses reveal any significant inconsistency (**eTable 3**). As such, this analysis was performed using the consistency model.

### Pairwise meta-analysis based on head-to-head comparisons

The risk of AEs associated with different treatment regimens was directly compared to those associated with chemotherapy. Relative to chemotherapy, a significantly reduced risk of AEs of any grade was observed for treatment with single agent atezolizumab (OR, 0.12, 95%CI, 0.02-0.51), whereas this risk was significantly increased for avelumab + chemotherapy (OR, 19.00, 95%CI, 2.00-710.00) and camrelizumab + chemotherapy (OR, 7.70, 95%CI, 1.80-44.00), and it was unchanged for other ICIs + chemotherapy. No significant differences in the risk of grade 3 or higher AEs were observed for any ICI + chemotherapy combination as compared to chemotherapy alone. (**eFigure 4**).

### Network meta-analysis of AEs in the overall population

**Figure 4:**
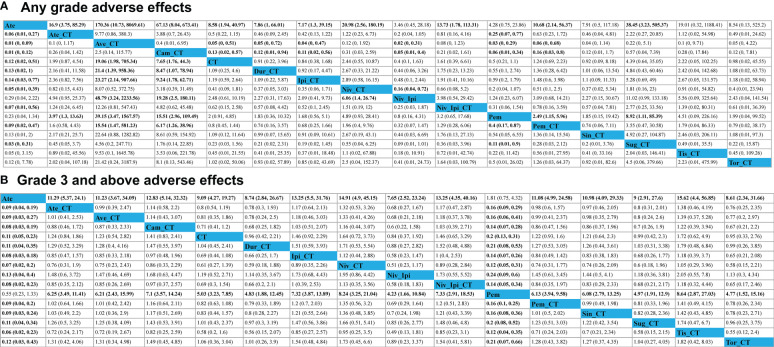
Safety profiles for adverse events of any grade **(A)** or adverse events of grade 3 or higher **(B)**. Each cell (light gray) in the safety profile includes pooled odds ratios and 95% confidence intervals, with significant results being shown in bold red text significant results are in bold. Pooled odds ratios and 95% confidence intervals correspond to outcomes for the upper treatment tier relative to the lower tier.

**Figure 5:**
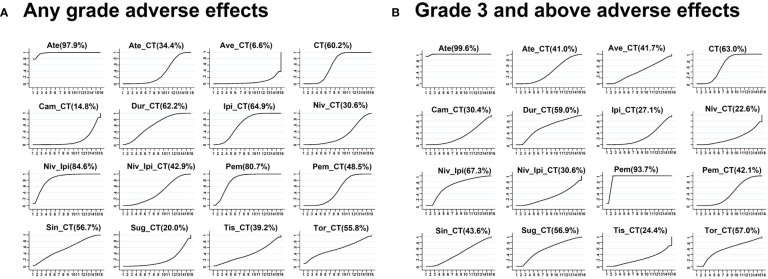
Network meta-analysis ranking diagram based on all adverse events **(A)** or grade 3 or higher adverse events **(B)**.

Avelumab + chemotherapy and camrelizumab + chemotherapy were linked to a significant increase in the risk of AEs of any grade relative to ipilimumab + chemotherapy (Ave: OR, 23.27, 95%CI, 2.14-907.66; Cam: OR, 9.24, 95%CI, 1.78-62.71), durvalumab + chemotherapy (Ave: OR, 21.40, 95%CI, 1.39-958.36; Cam: OR, 8.47, 95%CI, 1.07-78.94), and pembrolizumab + chemotherapy (Ave: OR, 15.54, 95%CI, 1.47-581.23; Cam: OR, 6.17, 95%CI, 1.26-38.96). Moreover, the risks of AEs of any grade associated with avelumab + chemotherapy (OR, 48.97, 95%CI, 3.24-2233.56), camrelizumab + chemotherapy (OR, 19.28, 95%CI, 2.50-180.11), and nivolumab + chemotherapy (OR, 6.06, 95%CI, 1.40-26.74) were significantly higher than those associated with the ipilimumab + nivolumab regimen (**Figure 4**). The rank order for these 16 treatment regimens in order of decreasing safety was as follows: atezolizumab (97.9%), nivolumab + ipilimumab (84.6%), pembrolizumab (80.7%), ipilimumab + chemotherapy (64.9%), durvalumab + chemotherapy (62.2%), chemotherapy (60.2%), sintilimab + chemotherapy (56.7%), toripalimab + chemotherapy (55.8%), pembrolizumab + chemotherapy (48.5%), nivolumab + ipilimumab + chemotherapy (42.9%), tislelizumab + chemotherapy (39.2%), atezolizumab + chemotherapy (34.4%), nivolumab + chemotherapy (30.6%), sugemalimab + chemotherapy (20.0%), camrelizumab + chemotherapy (14.8%), and avelumab + chemotherapy (6.6%) (**Figure 5**).

No significant differences in AEs of grade 3 or higher were observed when comparing the four evaluated treatment strategies (single-agent ICI + chemotherapy, two ICIs, two ICIs + chemotherapy, and chemotherapy alone) (**Figure 4**). ICI + chemotherapy regimens were associated with a higher risk of grade 3 or higher AEs relative to pembrolizumab monotherapy. The rank order for these 16 treatment regimens in order of decreasing safety was as follows: atezolizumab (99.6%), pembrolizumab (93.7%), nivolumab + ipilimumab (67.3%), chemotherapy (63.0%), durvalumab + chemotherapy (59.0%), toripalimab + chemotherapy (57.0%), sugemalimab + chemotherapy (56.9%), sintilimab + chemotherapy (43.6%), pembrolizumab + chemotherapy (42.1%), atezolizumab + chemotherapy (41.0%), nivolumab + ipilimumab + chemotherapy (30.6%), camrelizumab + chemotherapy (30.4%), ipilimumab + chemotherapy (27.1%), tislelizumab + chemotherapy (24.4%), and nivolumab + chemotherapy (22.6%) (**Figure 5**).

### Subgroup analyses for specific treatment-associated AEs

Only ipilimumab + chemotherapy was associated with an increase in the risk of hepatotoxicity of any grade relative to chemotherapy alone (ALT: OR, 0.23, 95%CI, 0.06-0.73; AST: OR, 0.22, 95%CI, 0.05-0.75). ICIs + chemotherapy treatment was not associated with a higher risk of hepatotoxicity relative to pembrolizumab monotherapy (**eFigure 5**). The regimens with the poorest safety profiles in this context were ipilimumab + chemotherapy (AST: 9.8%, ALT:10.7%) and atezolizumab + chemotherapy (AST: 12%, ALT:10.5%) (**eFigure 6**).

With respect to gastrointestinal toxicity of any grade, nivolumab + ipilimumab was associated with significantly better safety outcomes relative to outcomes for chemotherapy or ICIs + chemotherapy (**eFigure 5**). Ipilimumab + chemotherapy was associated with the greatest risk of gastrointestinal toxicity (nausea: 14.6%, decreased appetite: 8.3%, diarrhea: 2.6%) (**eFigure 6**).

With respect to hypothyroidism of any grade, all ICI + chemotherapy regimens other than those containing toripalimab were associated with an elevated risk relative to that observed for chemotherapy alone. A significantly higher risk of hypothyroidism was observed for durvalumab + chemotherapy relative to pembrolizumab + chemotherapy (OR, 8.90, 95%CI, 1.03-304.07) or toripalimab + chemotherapy (OR, 17.20, 95%CI, 1.43-680.94). Two-drug ICI regimens with or without chemotherapy were associated with an increased risk of hypothyroidism relative to single-drug ICI regimens with or without chemotherapy (**eFigure 5**).

With respect to cutaneous toxicity, the risk of pruritus was higher for camrelizumab + chemotherapy (OR, 3.11, 95%CI, 1.36-7.69), nivolumab + chemotherapy (OR, 15.32, 95%CI, 2.50-176.65), and pembrolizumab + chemotherapy (OR, 3.36, 95%CI, 1.38-8.29) relative to chemotherapy alone. Two-drug ICI and two-drug ICI + chemotherapy regimens were associated with an increased risk of pruritus, with no significant difference between these two regimens. This risk did not differ significantly among ICI + chemotherapy regimens with the exception of a higher observed risk for ipilimumab + chemotherapy (OR, 6.30, 95%CI, 1.81-23.13) relative to pembrolizumab + chemotherapy (**eFigure 5**). When ranking these different regimens, the odds of developing a rash were highest for ipilimumab + chemotherapy (**eFigure 6**).

Only pembrolizumab + chemotherapy (OR, 6.54, 95%CI, 1.60-80.83) was associated with an increase in the risk of pneumonia of any grade relative to chemotherapy alone, while no significant differences in the risk of this AE were observed among ICI + chemotherapy regimens or when comparing the remaining ICIs + chemotherapy regimens to chemotherapy alone (**eFigure 5**).

With respect to pyrexia of any grade, atezolizumab + chemotherapy (OR, 4.18, 95%CI, 1.50-12.11), avelumab + chemotherapy (OR, 2.29, 95%CI, 1.13-4.65), ipilimumab + chemotherapy (OR, 3.46, 95%CI, 1.45-9.04), and pembrolizumab + chemotherapy (OR, 1.73, 95%CI, 1.24-2.42) were associated with increased risk as compared to chemotherapy alone, while atezolizumab + chemotherapy (OR, 3.71, 95%CI, 1.23-11.77) and ipilimumab + chemotherapy (OR, 3.07, 95%CI, 1.17-8.72) were associated with increased risk relative to pembrolizumab monotherapy. Atezolizumab + chemotherapy also had a higher risk than sintilimab + chemotherapy (OR, 3.20, 95%CI, 1.05-10.24) (**eFigure 5**).

The risk of fatigue of any grade was lower for atezolizumab + chemotherapy (OR, 0.29, 95%CI, 0.09-0.84), avelumab + chemotherapy (OR, 0.25, 95%CI, 0.07-0.80), ipilimumab + chemotherapy (OR, 0.31, 95%CI, 0.09-0.92), pembrolizumab + chemotherapy (OR, 0.33, 95%CI, 0.10-0.92), and toripalimab + chemotherapy (OR, 0.27, 95%CI, 0.07-0.94) relative to sugemalimab + chemotherapy (**eFigure 5**).

No significant differences in the risk of grade 3 or higher AEs of any of the specific types discussed above were observed among the analyzed ICI + chemotherapy regimens, all of which exhibited comparable safety profiles at this level (**eFigure 7**).

### Sensitivity analyses

Next, sensitivity analyses specifically focusing on 20 clinical studies of lung cancer patients and 30 phase III RCTs revealed stable results in line with the original network meta-analysis (**eFigure 8**). Both subgroup analyzes of studies including only phase III RCTs or studies including only patients with lung cancer found acceptable heterogeneity between studies (I^2^<50%).Relatively minor changes were observed with respect to the rankings of nivolumab + ipilimumab + chemotherapy and ipilimumab + chemotherapy in lung cancer and phase III studies, respectively. Rates of grade 3 or higher AEs in lung cancer also exhibited a slight change in the ranking of camrelizumab + chemotherapy.

### Convergence and publication bias

**Figure 6:**
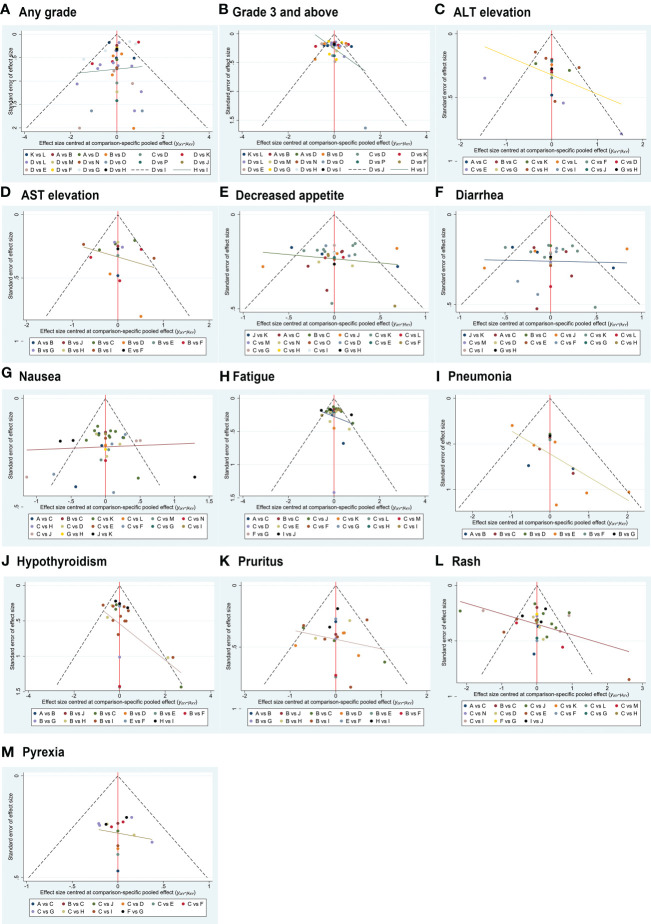
Funnel plot-based evaluation of publication bias. The symmetrical funnel plot distribution suggests a lack of publication bias.

Trace plots (**eFigure 9**) and Brooks-Gelman-Rubin diagnostic plots (**eFigure 10**) for all AE comparisons in this NMA exhibited good model convergence. Funnel plots for these analyses were also symmetrical, with no evidence of publication bias (**Figure 6**).

## Discussion

A growing number of combination ICI and chemotherapy regimens have been granted approval as first-line treatments for a variety of cancers, and even highly aggressive cancers of unknown primary (CUP) may be particularly sensitive to ICI, especially with tumor mutational burden > 10 mutations per megabase (50), leading to increased clinical interest in the risk of irAEs. However, there have not been any studies to date comprehensively analyzing the toxicity and safety profiles of different ICI and chemotherapy combination regimens. Results from the present network meta-analysis revealed that camrelizumab + chemotherapy and avelumab + chemotherapy were associated with a higher relative risk of AEs of any grade, while of the evaluated PD-1-based and PD-L1-based combination chemotherapy regimens, sintilimab + chemotherapy and durvalumab + chemotherapy were respectively associated with the best safety outcomes. However, with respect to the risk of grade 3 or higher AEs, which are of more significant clinical concern, all analyzed ICI and chemotherapy combination regimens exhibited similar risk profiles.

Several meta-analyses published to date have examined the toxicity profiles associated with ICI + chemotherapy regimens relative to chemotherapy alone or ICI + chemotherapy with immunotherapy (51–53), but the majority of these studies did not focus on the specific ICIs used, even those these agents exhibit different immunological mechanisms of action that may yield distinct AE risk profiles. In this study, we performed a comprehensive evaluation of the toxicity of different ICI + chemotherapy regimens and thereby determined that camrelizumab + chemotherapy and avelumab + chemotherapy were associated with a significant increase in the risk of AEs of any grade as compared to durvalumab + chemotherapy, ipilimumab + chemotherapy, or pembrolizumab + chemotherapy. Ipilimumab + chemotherapy exhibited reduced toxicity relative to camrelizumab + chemotherapy and avelumab + chemotherapy, which is unexpected given that CTLA-4 inhibitors are generally regarded as being more toxic than other ICIs. Many prior studies, however, have focused on AEs of any grade in patients undergoing ICI monotherapy-based treatment. For example, one analysis of atezolizumab, nivolumab, pembrolizumab, and durvalumab found durvalumab to exhibit the greatest toxicity of these ICIs (12). In a separate report, the rankings of analyzed ICIs based on rates of grade 1-5 AECs were atezolizumab, nivolumab, pembrolizumab, and ipilimumab in descending order (54). The differences among these studies may be attributable to changes in the safety profiles of ICI agents when administered in combination with chemotherapy owing to the immunosuppressive effects of these cytotoxic agents and the pretreatment use of cortisol.

Grade 3 or higher AEs often result in the discontinuation of treatment, and it is thus essential that clinicians be aware of these potential AEs and differences in their incidence rates associated with ICI + chemotherapy regimens in order to guide appropriate clinical decision-making efforts. Here, no significant differences in grade 3 or higher AE rates were observed among different ICI + chemotherapy regimens, even when examining specific AEs. Peng et al. previously detected a reduction in the risk of grade 3 or higher AEs in patients undergoing pembrolizumab + chemotherapy treatment relative to those being treated with ipilimumab + chemotherapy, nivolumab + chemotherapy, or atezolizumab + chemotherapy. Moreover, Li et al. determined that nivolumab + chemotherapy was associated with the risk of grade 3 or higher AEs as compared to pembrolizumab + chemotherapy or camrelizumab + chemotherapy (55). Our findings, however, are in line with data published by Liu et al. and Chen et al., both of whom observed no significant differences in grade 3 or higher AE rates among pembrolizumab + chemotherapy, ipilimumab + chemotherapy, atezolizumab + chemotherapy, and durvalumab + chemotherapy regimens (56, 57). These results also partially support the findings of Zhou et al., who did not observe any significant increase in rates of serious AEs when comparing PD-1 inhibitor + chemotherapy and PD-L1 inhibitor + chemotherapy groups (58).

In addition to evaluating the relative rates of specific AEs associated with particular ICI + chemotherapy regimens, it is of value for clinicians to understand the general toxicity profile of each such regimen. The present results suggested that rates of gastrointestinal toxicity were highest in the context of ipilimumab + chemotherapy treatment, in line with several prior reports (54, 56). One meta-analysis examining the rates of immune-related hepatotoxicity in solid tumor patients observed a higher risk of AST and ALT elevation in patients undergoing PD-1 inhibitor treatment relative to patients undergoing PD-L1 inhibitor treatment, with the highest odds of hepatotoxicity being observed for pembrolizumab and nivolumab (59). This is not consistent with our findings, given that atezolizumab + chemotherapy and ipilimumab + chemotherapy were herein identified as the regimens most closely associated with a risk of hepatotoxicity. Cutaneous complications are among the most common and earliest irAEs observed in patients undergoing ICI treatment (60), with anti-CTLA-4 having been linked to higher rates of such toxicity (61). In this NMA, of the analyzed single-agent ICI + chemotherapy regimens, nivolumab + chemotherapy and ipilimumab + chemotherapy were associated with the highest risk of skin toxicity, echoing data published previously by Xu et al., who found ipilimumab to be primarily associated with skin toxicity (54). Several reports have linked ICI + chemotherapy regimens to a reduction in pneumonia risk relative to two-drug ICI combinations or ICI monotherapy. Moreover, Khoja et al. posited that pneumonia and hypothyroidism were complications more commonly observed in the context of PD-1 checkpoint blockade as compared to CTLA-4 checkpoint blockade (62). Zhang et al. additionally detected an increase in the risk of pneumonia in individuals undergoing pembrolizumab treatment, while nivolumab was associated with a greater risk of hypothyroidism (63, 64). Our results are consistent with these prior findings, as pembrolizumab + chemotherapy and nivolumab + chemotherapy were the most common respective causes of pneumonia and hypothyroidism. These findings offer potential safety-related information that can guide future clinical decision-making.

Reported irAE incidence rates for CTLA-4 inhibitors were 72% for AEs of any grade and 24% for AEs of grade 3 or higher (65), while for PD-1 or PD-L1 inhibitors these respective irAE rates were lower at 27% and 6% (66). PD-L1 inhibitors are generally regarded as the safest ICIs currently available owing to their PD-L2-sparing activity, thus preserving appropriate immunological homeostasis. In line with prior reports from Cheng et al. (54), atezolizumab was herein found to exhibit the best overall safety profile. Of the analyzed ICI + chemotherapy regimens, however, durvalumab + chemotherapy and sintilimab + chemotherapy were found to be relatively safe PD-L1 inhibitor- and PD-1 inhibitor-containing regimens, respectively. Similarly, another report recently found durvalumab + chemotherapy regimens to be associated with better safety profiles than atezolizumab + chemotherapy and pembrolizumab + chemotherapy regimens (67). While the mechanistic basis for these results remains to be clarified, these findings nonetheless offer valuable guidance for oncologists seeking to choose the optimal immunotherapeutic regimens for patients in their care.

The present study was focused on toxicity outcomes associated with different regimens across all cancer types, raising the question of whether these safety profiles are tumor-type-dependent. One recent review suggests that irAE frequencies are largely dependent on the type of drug used (60), while another study detected no relationship between tumor type and irAE incidence (68). Sensitivity analyses in this study focused specifically on lung cancer patients did not reveal any significant differences in the safety rankings of these different therapeutic regimens, further supporting a model wherein tumor type largely fails to impact the occurrence of irAEs.

However, there are certain limitations to this study. For one, the detection and diagnosis of AEs and irAEs are largely based upon the clinical experience of individual physicians, potentially introducing some degree of bias into the diagnostic process. Second, certain irAEs may have a delayed onset such that they were not identified within the follow-up period of particular RCTs, leading to missing data. Third, the media follow-up duration in each included RCT differed, potentially contributing to increased rates of immunotherapy-related irAEs and associated confounding factors. Fourth, high levels of heterogeneity were evident for some of these comparisons, potentially contributing to the under- or overestimation of certain results even though a random effects model was used. Fifth, no effort was made to control for high-risk factors with the potential to impact AEs such as age, history of smoking, or PS score, potentially confounding these results. Despite these limitations, we believe that the results of this study are meaningful and can aid clinicians in selecting an appropriate therapeutic regimen with the best efficacy and safety profile for their patients.

## Conclusions

This present network meta-analysis was developed to explore the incidence and risk of AEs associated with different ICI + chemotherapy regimens in cancer patients. The risk of AEs of any grade was found to vary among analyzed therapeutic regimens, with higher AE rates for regimens consisting of chemotherapy combined with avelumab or camrelizumab, whereas this risk was lower for combination regimens including durvalumab/sintilimab. While variability was observed with respect to the toxicity profiles of these analyzed ICI + chemotherapy regimens, no significant differences in the risk of severe AEs were evident among these regimens, with this latter concern being of particularly high importance when selecting the most appropriate immunotherapeutic regimen to use in a clinical setting.

## Data availability statement

The raw data supporting the conclusions of this article will be made available by the authors, without undue reservation.

## Author contributions

Conceptualization: YLG, TM. Methodology: TM. Data curation: YLG, TM, QYD. Writing- Original draft preparation: TM. Supervision: YLG. Writing- Reviewing and Editing: YLG, TM, QYD. Manuscript revision: TW, TM. All authors contributed to the article and approved the submitted version.

## References

[1] Sharon E Streicher H Goncalves P Chen HX. Immune checkpoint inhibitors in clinical trials. Chin J Cancer (2014) 33(9):434–44. doi: 10.5732/cjc.014.10122 PMC4190433 25189716

[2] Yang Y. Cancer immunotherapy: harnessing the immune system to battle cancer. J Clin Invest (2015) 125(9):3335–7. doi: 10.1172/JCI83871 PMC4588312 26325031

[3] Larroquette M Domblides C Lefort F Lasserre M Quivy A Sionneau B. Combining immune checkpoint inhibitors with chemotherapy in advanced solid tumours: A review. Eur J Cancer (2021) 158:47–62. doi: 10.1016/j.ejca.2021.09.013 34655837

[4] Revythis A Limbu A Mikropoulos C Ghose A Sanchez E Sheriff M. Recent insights into PARP and immuno-checkpoint inhibitors in epithelial ovarian cancer. Int J Environ Res Public Health (2022) 19(14):8577. doi: 10.3390/ijerph19148577 35886427 PMC9317199

[5] Livshits Z Rao RB Smith SW. An approach to chemotherapy-associated toxicity. Emerg Med Clin North Am (2014) 32(1):167–203. doi: 10.1016/j.emc.2013.09.002 24275174

[6] Fox P Darley A Furlong E Miaskowski C Patiraki E Armes J. The assessment and management of chemotherapy-related toxicities in patients with breast cancer, colorectal cancer, and hodgkin's and non-hodgkin's lymphomas: A scoping review. Eur J Oncol Nurs (2017) 26:63–82. doi: 10.1016/j.ejon.2016.12.008 28069154

[7] Brahmer JR Abu-Sbeih H Ascierto PA Brufsky J Cappelli LC Cortazar FB. Society for immunotherapy of cancer (SITC) clinical practice guideline on immune checkpoint inhibitor-related adverse events. J Immunother Cancer (2021) 9(6):e002435. doi: 10.1136/jitc-2021-002435 34172516 PMC8237720

[8] Berti A Bortolotti R Dipasquale M Kinspergher S Prokop L Grandi G. Meta-analysis of immune-related adverse events in phase 3 clinical trials assessing immune checkpoint inhibitors for lung cancer. Crit Rev Oncol Hematol (2021) 162:103351. doi: 10.1016/j.critrevonc.2021.103351 33989769

[9] Chen CY Huang CH Chen WC Huang MS Wei YF. Comparative safety of immune checkpoint inhibitors and chemotherapy in advanced non-small cell lung cancer: A systematic review and network meta-analysis. Int Immunopharmacol (2022) 108:108848. doi: 10.1016/j.intimp.2022.108848 35597121

[10] Yan YD Cui JJ Fu J Su YJ Chen XY Gu ZC. A network comparison on safety profiling of immune checkpoint inhibitors in advanced lung cancer. Front Immunol (2021) 12:760737. doi: 10.3389/fimmu.2021.760737 34925331 PMC8677695

[11] Xu Q Zhang X Huang M Dai X Gao J Li S. Comparison of efficacy and safety of single and double immune checkpoint inhibitor-based first-line treatments for advanced driver-gene wild-type non-small cell lung cancer: A systematic review and network meta-analysis. Front Immunol (2021) 12:731546. doi: 10.3389/fimmu.2021.731546 34484242 PMC8415225

[12] Liang J Li M Sui Q Hu Z Bian Y Huang Y. Compare the efficacy and safety of programmed cell death-1 (PD-1) and programmed cell death ligand-1 (PD-L1) inhibitors for advanced non-small cell lung cancer: a Bayesian analysis. Transl Lung Cancer Res (2020) 9(4):1302–23. doi: 10.21037/tlcr-20-192 PMC7481633 32953506

[13] Mills EJ Thorlund K Ioannidis JP. Demystifying trial networks and network meta-analysis. Bmj (2013) 346:f2914. doi: 10.1136/bmj.f2914 23674332

[14] Brooks SP Gelman A. General methods for monitoring convergence of iterative simulations. J Comput Graphical Statistics (1998) 7(4):434–55. doi: 10.1080/10618600.1998.10474787

[15] Salanti G Ades AE Ioannidis JP. Graphical methods and numerical summaries for presenting results from multiple-treatment meta-analysis: an overview and tutorial. J Clin Epidemiol (2011) 64(2):163–71. doi: 10.1016/j.jclinepi.2010.03.016 20688472

[16] Egger M Davey Smith G Schneider M Minder C. Bias in meta-analysis detected by a simple, graphical test. Bmj (1997) 315(7109):629–34. doi: 10.1136/bmj.315.7109.629 PMC2127453 9310563

[17] Galsky MD Arija JÁChecktae Bamias A Davis ID De Santis M Kikuchi E. Atezolizumab with or without chemotherapy in metastatic urothelial cancer (IMvigor130): a multicentre, randomised, placebo-controlled phase 3 trial. Lancet (2020) 395(10236):1547–57. doi: 10.1016/S0140-6736(20)30230-0 32416780

[18] Powles T Csőszi T Özgüroğlu M Matsubara N Géczi L Cheng SY. Pembrolizumab alone or combined with chemotherapy versus chemotherapy as first-line therapy for advanced urothelial carcinoma (KEYNOTE-361): a randomised, open-label, phase 3 trial. Lancet Oncol (2021) 22(7):931–45. doi: 10.1016/S1470-2045(21)00152-2 34051178

[19] Yang Y Qu S Li J Hu C Xu M Li W. Camrelizumab versus placebo in combination with gemcitabine and cisplatin as first-line treatment for recurrent or metastatic nasopharyngeal carcinoma (CAPTAIN-1st): a multicentre, randomised, double-blind, phase 3 trial. Lancet Oncol (2021) 22(8):1162–74. doi: 10.1016/S1470-2045(21)00302-8 34174189

[20] Mai HQ Chen QY Chen D Hu C Yang K Wen J. Toripalimab or placebo plus chemotherapy as first-line treatment in advanced nasopharyngeal carcinoma: a multicenter randomized phase 3 trial. Nat Med (2021) 27(9):1536–43. doi: 10.1038/s41591-021-01444-0 34341578

[21] Burtness B Harrington KJ Greil R Soulières D Tahara M de Castro G Jr.. Pembrolizumab alone or with chemotherapy versus cetuximab with chemotherapy for recurrent or metastatic squamous cell carcinoma of the head and neck (KEYNOTE-048): a randomised, open-label, phase 3 study. Lancet (2019) 394(10212):1915–28. doi: 10.1016/S0140-6736(19)32591-7 31679945

[22] Luo H Lu J Bai Y Mao T Wang J Fan Q. Effect of camrelizumab vs placebo added to chemotherapy on survival and progression-free survival in patients with advanced or metastatic esophageal squamous cell carcinoma: The ESCORT-1st randomized clinical trial. Jama (2021) 326(10):916–25. doi: 10.1001/jama.2021.12836 PMC8441593 34519801

[23] Shitara K Van Cutsem E Bang YJ Fuchs C Wyrwicz L Lee KW. Efficacy and safety of pembrolizumab or pembrolizumab plus chemotherapy vs chemotherapy alone for patients with first-line, advanced gastric cancer: The KEYNOTE-062 phase 3 randomized clinical trial. JAMA Oncol (2020) 6(10):1571–80. doi: 10.1001/jamaoncol.2020.3370 PMC7489405 32880601

[24] Sun JM Shen L Shah MA Enzinger P Adenis A Doi T. Pembrolizumab plus chemotherapy versus chemotherapy alone for first-line treatment of advanced oesophageal cancer (KEYNOTE-590): a randomised, placebo-controlled, phase 3 study. Lancet (2021) 398(10302):759–71. doi: 10.1016/S0140-6736(21)01234-4 34454674

[25] Doki Y Ajani JA Kato K Xu J Wyrwicz L Motoyama S. Nivolumab combination therapy in advanced esophageal squamous-cell carcinoma. N Engl J Med (2022) 386(5):449–62. doi: 10.1056/NEJMoa2111380 35108470

[26] Zhou C Wang Z Sun Y Cao L Ma Z Wu R. Sugemalimab versus placebo, in combination with platinum-based chemotherapy, as first-line treatment of metastatic non-small-cell lung cancer (GEMSTONE-302): interim and final analyses of a double-blind, randomised, phase 3 clinical trial. Lancet Oncol (2022) 23(2):220–33. doi: 10.1016/S1470-2045(21)00650-1 35038432

[27] Jotte R Cappuzzo F Vynnychenko I Stroyakovskiy D Rodríguez-Abreu D Hussein M. Atezolizumab in combination with carboplatin and nab-paclitaxel in advanced squamous NSCLC (IMpower131): Results from a randomized phase III trial. J Thorac Oncol (2020) 15(8):1351–60. doi: 10.1016/j.jtho.2020.03.028 32302702

[28] West H McCleod M Hussein M Morabito A Rittmeyer A Conter HJ. Atezolizumab in combination with carboplatin plus nab-paclitaxel chemotherapy compared with chemotherapy alone as first-line treatment for metastatic non-squamous non-small-cell lung cancer (IMpower130): a multicentre, randomised, open-label, phase 3 trial. Lancet Oncol (2019) 20(7):924–37. doi: 10.1016/S1470-2045(19)30167-6 31122901

[29] Gadgeel S Rodríguez-Abreu D Speranza G Esteban E Felip E Dómine M. Updated analysis from KEYNOTE-189: Pembrolizumab or placebo plus pemetrexed and platinum for previously untreated metastatic nonsquamous non-Small-Cell lung cancer. J Clin Oncol (2020) 38(14):1505–17. doi: 10.1200/JCO.19.03136 32150489

[30] Paz-Ares L Vicente D Tafreshi A Robinson A Soto Parra H Mazières J. A randomized, placebo-controlled trial of pembrolizumab plus chemotherapy in patients with metastatic squamous NSCLC: Protocol-specified final analysis of KEYNOTE-407. J Thorac Oncol (2020) 15(10):1657–69. doi: 10.1016/j.jtho.2020.06.015 32599071

[31] Langer CJ Gadgeel SM Borghaei H Papadimitrakopoulou VA Patnaik A Powell SF. Carboplatin and pemetrexed with or without pembrolizumab for advanced, non-squamous non-small-cell lung cancer: a randomised, phase 2 cohort of the open-label KEYNOTE-021 study. Lancet Oncol (2016) 17(11):1497–508. doi: 10.1016/S1470-2045(16)30498-3 PMC6886237 27745820

[32] Lu S Wang J Yu Y Yu X Hu Y Ai X. Tislelizumab plus chemotherapy as first-line treatment for locally advanced or metastatic nonsquamous NSCLC (RATIONALE 304): A randomized phase 3 trial. J Thorac Oncol (2021) 16(9):1512–22. doi: 10.1016/j.jtho.2021.05.005 34033975

[33] Wang J Lu S Yu X Hu Y Sun Y Wang Z. Tislelizumab plus chemotherapy vs chemotherapy alone as first-line treatment for advanced squamous non-Small-Cell lung cancer: A phase 3 randomized clinical trial. JAMA Oncol (2021) 7(5):709–17. doi: 10.1001/jamaoncol.2021.0366 PMC8017481 33792623

[34] Paz-Ares L Dvorkin M Chen Y Reinmuth N Hotta K Trukhin D. Durvalumab plus platinum-etoposide versus platinum-etoposide in first-line treatment of extensive-stage small-cell lung cancer (CASPIAN): a randomised, controlled, open-label, phase 3 trial. Lancet (2019) 394(10212):1929–39. doi: 10.1016/S0140-6736(19)32222-6 31590988

[35] Rudin CM Awad MM Navarro A Gottfried M Peters S Csőszi T. Pembrolizumab or placebo plus etoposide and platinum as first-line therapy for extensive-stage small-cell lung cancer: Randomized, double-blind, phase III KEYNOTE-604 study. J Clin Oncol (2020) 38(21):2369–79. doi: 10.1200/JCO.20.00793 PMC7474472 32468956

[36] Horn L Mansfield AS Szczęsna A Havel L Krzakowski M Hochmair MJ. First-line atezolizumab plus chemotherapy in extensive-stage small-cell lung cancer. N Engl J Med (2018) 379(23):2220–9. doi: 10.1056/NEJMoa1809064 30280641

[37] Reck M Luft A Szczesna A Havel L Kim SW Akerley W. Phase III randomized trial of ipilimumab plus etoposide and platinum versus placebo plus etoposide and platinum in extensive-stage small-cell lung cancer. J Clin Oncol (2016) 34(31):3740–8. doi: 10.1200/JCO.2016.67.6601 27458307

[38] Reck M Bondarenko I Luft A Serwatowski P Barlesi F Chacko R. Ipilimumab in combination with paclitaxel and carboplatin as first-line therapy in extensive-disease-small-cell lung cancer: results from a randomized, double-blind, multicenter phase 2 trial. Ann Oncol (2013) 24(1):75–83. doi: 10.1093/annonc/mds213 22858559

[39] Paz-Ares L Ciuleanu TE Cobo M Schenker M Zurawski B Menezes J. First-line nivolumab plus ipilimumab combined with two cycles of chemotherapy in patients with non-small-cell lung cancer (CheckMate 9LA): an international, randomised, open-label, phase 3 trial. Lancet Oncol (2021) 22(2):198–211. doi: 10.1016/S1470-2045(20)30641-0 33476593

[40] Zhou C Chen G Huang Y Zhou J Lin L Feng J. Camrelizumab plus carboplatin and pemetrexed versus chemotherapy alone in chemotherapy-naive patients with advanced non-squamous non-small-cell lung cancer (CameL): a randomised, open-label, multicentre, phase 3 trial. Lancet Respir Med (2021) 9(3):305–14. doi: 10.1016/S2213-2600(20)30365-9 33347829

[41] Yang Y Wang Z Fang J Yu Q Han B Cang S. Efficacy and safety of sintilimab plus pemetrexed and platinum as first-line treatment for locally advanced or metastatic nonsquamous NSCLC: a randomized, double-blind, phase 3 study (Oncology pRogram by InnovENT anti-PD-1-11). J Thorac Oncol (2020) 15(10):1636–46. doi: 10.1016/j.jtho.2020.07.014 32781263

[42] Nishio M Barlesi F West H Ball S Bordoni R Cobo M. Atezolizumab plus chemotherapy for first-line treatment of nonsquamous NSCLC: Results from the randomized phase 3 IMpower132 trial. J Thorac Oncol (2021) 16(4):653–64. doi: 10.1016/j.jtho.2020.11.025 33333328

[43] Nishio M Saito H Goto K Watanabe S Sueoka-Aragane N Okuma Y. IMpower132: Atezolizumab plus platinum-based chemotherapy vs chemotherapy for advanced NSCLC in Japanese patients. Cancer Sci (2021) 112(4):1534–44. doi: 10.1111/cas.14817 PMC8019191 33462883

[44] Govindan R Szczesna A Ahn MJ Schneider CP Gonzalez Mella PF Barlesi F. Phase III trial of ipilimumab combined with paclitaxel and carboplatin in advanced squamous non-Small-Cell lung cancer. J Clin Oncol (2017) 35(30):3449–57. doi: 10.1200/JCO.2016.71.7629 28854067

[45] Lynch TJ Bondarenko I Luft A Serwatowski P Barlesi F Chacko R. Ipilimumab in combination with paclitaxel and carboplatin as first-line treatment in stage IIIB/IV non-small-cell lung cancer: results from a randomized, double-blind, multicenter phase II study. J Clin Oncol (2012) 30(17):2046–54. doi: 10.1200/JCO.2011.38.4032 22547592

[46] Zhou C Wu L Fan Y Wang Z Liu L Chen G. Sintilimab plus platinum and gemcitabine as first-line treatment for advanced or metastatic squamous NSCLC: Results from a randomized, double-blind, phase 3 trial (ORIENT-12). J Thorac Oncol (2021) 16(9):1501–11. doi: 10.1016/j.jtho.2021.04.011 34048947

[47] Monk BJ Colombo N Oza AM Fujiwara K Birrer MJ Randall L. Chemotherapy with or without avelumab followed by avelumab maintenance versus chemotherapy alone in patients with previously untreated epithelial ovarian cancer (JAVELIN ovarian 100): an open-label, randomised, phase 3 trial. Lancet Oncol (2021) 22(9):1275–89. doi: 10.1016/S1470-2045(21)00342-9 34363762

[48] Cortes J Cescon DW Rugo HS Nowecki Z Im SA Yusof MM. Pembrolizumab plus chemotherapy versus placebo plus chemotherapy for previously untreated locally recurrent inoperable or metastatic triple-negative breast cancer (KEYNOTE-355): a randomised, placebo-controlled, double-blind, phase 3 clinical trial. Lancet (2020) 396(10265):1817–28. doi: 10.1016/S0140-6736(20)32531-9 33278935

[49] Schmid P Cortes J Pusztai L McArthur H Kümmel S Bergh J. Pembrolizumab for early triple-negative breast cancer. N Engl J Med (2020) 382(9):810–21. doi: 10.1056/NEJMoa1910549 32101663

[50] Rassy E Boussios S Pavlidis N. Genomic correlates of response and resistance to immune checkpoint inhibitors in carcinomas of unknown primary. Eur J Clin Invest (2021) 51(9):e13583. doi: 10.1111/eci.13583 33970501

[51] Wang M Liang H Wang W Zhao S Cai X Zhao Y. Immune-related adverse events of a PD-L1 inhibitor plus chemotherapy versus a PD-L1 inhibitor alone in first-line treatment for advanced non-small cell lung cancer: A meta-analysis of randomized control trials. Cancer (2021) 127(5):777–86. doi: 10.1002/cncr.33270 33119182

[52] Dafni U Tsourti Z Vervita K Peters S. Immune checkpoint inhibitors, alone or in combination with chemotherapy, as first-line treatment for advanced non-small cell lung cancer. a systematic review and network meta-analysis. Lung Cancer (2019) 134:127–40. doi: 10.1016/j.lungcan.2019.05.029 31319971

[53] Liu L Bai H Wang C Seery S Wang Z Duan J. Efficacy and safety of first-line immunotherapy combinations for advanced NSCLC: A systematic review and network meta-analysis. J Thorac Oncol (2021) 16(7):1099–117. doi: 10.1016/j.jtho.2021.03.016 33839365

[54] Xu C Chen YP Du XJ Liu JQ Huang CL Chen L. Comparative safety of immune checkpoint inhibitors in cancer: systematic review and network meta-analysis. Bmj (2018) 363:k4226. doi: 10.1136/bmj.k4226 30409774 PMC6222274

[55] Li ZC Sun YT Lai MY Zhou YX Qiu MZ. Efficacy and safety of PD-1 inhibitors combined with chemotherapy as first-line therapy for advanced esophageal cancer: A systematic review and network meta-analysis. Int Immunopharmacol (2022) 109:108790. doi: 10.1016/j.intimp.2022.108790 35504202

[56] Liu T Jin B Chen J Wang H Lin S Dang J. Comparative risk of serious and fatal treatment-related adverse events caused by 19 immune checkpoint inhibitors used in cancer treatment: a network meta-analysis. Ther Adv Med Oncol (2020) 12:1758835920940927. doi: 10.1177/1758835920940927 32774474 PMC7394035

[57] Chen HL Tu YK Chang HM Lee TH Wu KL Tsai YC. Systematic review and network meta-analysis of immune checkpoint inhibitors in combination with chemotherapy as a first-line therapy for extensive-stage small cell carcinoma. Cancers (Basel) (2020) 12(12):3629. doi: 10.3390/cancers12123629 33287455 PMC7761843

[58] Zhou C Li M Wang Z An D Li B. Adverse events of immunotherapy in non-small cell lung cancer: A systematic review and network meta-analysis. Int Immunopharmacol (2022) 102:108353. doi: 10.1016/j.intimp.2021.108353 34883352

[59] Deng S Yang Q Shu X Lang J Lu S. The relative risk of immune-related liver dysfunction of PD-1/PD-L1 inhibitors versus chemotherapy in solid tumors: A meta-analysis of randomized controlled trials. Front Pharmacol (2019) 10:1063. doi: 10.3389/fphar.2019.01063 31607917 PMC6767975

[60] Martins F Sofiya L Sykiotis GP Lamine F Maillard M Fraga M. Adverse effects of immune-checkpoint inhibitors: epidemiology, management and surveillance. Nat Rev Clin Oncol (2019) 16(9):563–80. doi: 10.1038/s41571-019-0218-0 31092901

[61] Chen CH Yu HS Yu S. Cutaneous adverse events associated with immune checkpoint inhibitors: A review article. Curr Oncol (2022) 29(4):2871–86. doi: 10.3390/curroncol29040234 PMC9032875 35448208

[62] Khoja L Day D Wei-Wu Chen T Siu LL Hansen AR. Tumour- and class-specific patterns of immune-related adverse events of immune checkpoint inhibitors: a systematic review. Ann Oncol (2017) 28(10):2377–85. doi: 10.1093/annonc/mdx286 28945858

[63] Zhang W Gu J Bian C Huang G. Immune-related adverse events associated with immune checkpoint inhibitors for advanced non-small cell lung cancer: A network meta-analysis of randomized clinical trials. Front Pharmacol (2021) 12:686876. doi: 10.3389/fphar.2021.686876 34759817 PMC8574003

[64] Gu J Shi L Jiang X Wen J Zheng X Cai H. Severe immune-related adverse events of immune checkpoint inhibitors for advanced non-small cell lung cancer: a network meta-analysis of randomized clinical trials. Cancer Immunol Immunother (2022) 71(9):2239–54. doi: 10.1007/s00262-022-03140-5 PMC10992828 35124713

[65] Bertrand A Kostine M Barnetche T Truchetet ME Schaeverbeke T. Immune related adverse events associated with anti-CTLA-4 antibodies: systematic review and meta-analysis. BMC Med (2015) 13:211. doi: 10.1186/s12916-015-0455-8 26337719 PMC4559965

[66] Wang PF Chen Y Song SY Wang TJ Ji WJ Li SW. Immune-related adverse events associated with anti-PD-1/PD-L1 treatment for malignancies: A meta-analysis. Front Pharmacol (2017) 8:730. doi: 10.3389/fphar.2017.00730 29093678 PMC5651530

[67] Ando K Manabe R Kishino Y Kusumoto S Yamaoka T Tanaka A. Comparative efficacy and safety of immunotherapeutic regimens with PD-1/PD-L1 inhibitors for previously untreated extensive-stage small cell lung cancer: A systematic review and network meta-analysis. Curr Oncol (2021) 28(2):1094–113. doi: 10.3390/curroncol28020106 PMC8025754 33673470

[68] Barroso-Sousa R Barry WT Garrido-Castro AC Hodi FS Min L Krop IE. Incidence of endocrine dysfunction following the use of different immune checkpoint inhibitor regimens: A systematic review and meta-analysis. JAMA Oncol (2018) 4(2):173–82. doi: 10.1001/jamaoncol.2017.3064 PMC5838579 28973656

