# Supplementary material for: The safety of combining immune checkpoint inhibitors and platinum-based chemotherapy for the treatment of solid tumors: A systematic review and network meta-analysis
Source: Front Immunol. 2023 Feb 7;14:1062679. doi: 10.3389/fimmu.2023.1062679 (PMC9941623; doi:10.3389/fimmu.2023.1062679)
Supplement: Supplementary file 1 [file DataSheet_1.pdf]

Supplementary Online Content

eTable 1. PRISMA extension checklist for this network meta-analysis

PICOS = population, intervention, comparators, outcomes, study design. *Italicized text denotes wording specific to network meta-analysis reporting added to guidance from the PRISMA statement. † Authors may utilize appendices to provide the full details pertaining to items included in this section.

eTable 2. Search strategy

eTable 3. Network meta-analysis node-splitting analysis

AE, Adverse effect; Ate, Atezolizumab; CT, Chemotherapy; Ate_CT, Atezolizumab + chemotherapy; Ave_CT, Avelumab + chemotherapy; Cam_CT, Camrelizumab + chemotherapy; Dur_CT, Durvalumab + chemotherapy; Ipi_CT, Ipilimumab + chemotherapy; Niv_CT, Nivolumab + chemotherapy; Niv_Ipi, Nivolumab + Ipilimumab ; Niv_Ipi_CT, Nivolumab + Ipilimumab + chemotherapy; Pem, Pembrolizumab; Pem_CT, Pembrolizumab + chemotherapy; Sin_CT, Sintilimab + chemotherapy; Sug_CT, Sugemalimab + chemotherapy; Tis_CT, Tislelizumab + chemotherapy; Tor_CT, Toripalimab + chemotherapy.

eFigure 1. Specific adverse event-based network maps

eFigure 2. Heterogeneity analyses

I^2 values < 25%, from 25% - 50%, and > 50% respectively represent low, medium and high heterogeneity.

eFigure 3. Inconsistency Analysis

P > 0.05 denotes the absence of significant inconsistency.

eFigure 4. Direct comparisons of treatments based on the risk of adverse events

eFigure 5. Safety profiles based on specific adverse events

eFigure 6. Specific adverse event-based ranking diagram

eFigure 7. Safety profiles based on specific grade 3 or higher adverse events

eFigure 8. Network meta-analysis sensitivity analysis

A-B, Only phase III clinical studies were included in this network meta-analysis; C-D, Only lung cancer patients were included in the network meta-analysis.

eFigure 9. Trace plot for the evaluation of model convergence

Overlapping MCMC chain area accounts for most of the chain fluctuation range, the fluctuation of an individual chain was not apparent, and density map distribution pattern was normal, consistent with satisfactory model convergence.

eFigure 10. Brooks-Gelman-Rubin diagnostic plots for the evaluation of model convergence

The median shrink factor and its 97.5% value rapidly converge to 1 and stabilize, consistent with a satisfactory level of model convergence.

eTable 1. PRISMA extension checklist for this network meta-analysis

Section/Topic	Item #	Checklist Item	Reported on Page #
**TITLE**			
Title	1	Identify the report as a systematic review *incorporating a network meta-analysis (or related form of meta-analysis).*	**1**
**ABSTRACT**			**2-3**
Structured summary	2	Provide a structured summary including, as applicable:<br>**Background:** main objectives<br>**Methods:** data sources; study eligibility criteria, participants, and interventions; study appraisal; and *synthesis methods, such as network meta-analysis.*<br>**Results:** number of studies and participants identified; summary estimates with corresponding confidence/credible intervals; *treatment rankings may also be discussed. Authors may choose to summarize pairwise comparisons against a chosen treatment included in their analyses for brevity.*<br>**Discussion/Conclusions:** limitations; conclusions and implications of findings.<br>**Other:** primary source of funding; systematic review registration number with registry name.	
**INTRODUCTION**			
Rationale	3	Describe the rationale for the review in the context of what is already known*, including mention of why a network meta-analysis has been conducted.*_	**4-5**
Objectives	4	Provide an explicit statement of questions being addressed, with reference to participants, interventions, comparisons, outcomes, and study design (PICOS).	**4-5**
**METHODS**			
Protocol and registration	5	Indicate whether a review protocol exists and if and where it can be accessed (e.g., Web address); and, if available, provide registration information, including registration number.	**5**

Eligibility criteria	6	Specify study characteristics (e.g., PICOS, length of follow-up) and report characteristics (e.g., years considered, language, publication status) used as criteria for eligibility, giving rationale. *Clearly describe eligible treatments included in the treatment network, and note whether any have been clustered or merged into the same node (with justification).*_	**6**
Information sources	7	Describe all information sources (e.g., databases with dates of coverage, contact with study authors to identify additional studies) in the search and date last searched.	**6**
Search	8	Present full electronic search strategy for at least one database, including any limits used, such that it could be repeated.	**Appendix page 7-8**
Study selection	9	State the process for selecting studies (i.e., screening, eligibility, included in systematic review, and, if applicable, included in the meta-analysis).	**6**
Data collection process	10	Describe method of data extraction from reports (e.g., piloted forms, independently, in duplicate) and any processes for obtaining and confirming data from investigators.	**6-8**
Data items	11	List and define all variables for which data were sought (e.g., PICOS, funding sources) and any assumptions and simplifications made.	**6-8**
**Geometry of the network**	**S1**	Describe methods used to explore the geometry of the treatment network under study and potential biases related to it. This should include how the evidence base has been graphically summarized for presentation, and what characteristics were compiled and used to describe the evidence base to readers.	**8**
Risk of bias within individual studies	12	Describe methods used for assessing risk of bias of individual studies (including specification of whether this was done at the study or outcome level), and how this information is to be used in any data synthesis.	**7-8**
Summary measures	13	State the principal summary measures (e.g., risk ratio, difference in means). *Also describe the use of additional summary measures assessed, such as treatment rankings and surface under the cumulative ranking curve (SUCRA) values, as well as modified approaches used to present summary findings from meta-analyses.*	**7-8**

Planned methods of analysis	14	Describe the methods of handling data and combining results of studies for each network meta-analysis. This should include, but not be limited to:<br>• *Handling of multi-arm trials;*<br>• *Selection of variance structure;*<br>• *Selection of prior distributions in Bayesian analyses; and*<br>• *Assessment of model fit.*	**7-8**
**Assessment of Inconsistency**	**S2**	Describe the statistical methods used to evaluate the agreement of direct and indirect evidence in the treatment network(s) studied. Describe efforts taken to address its presence when found.	**7-8**
Risk of bias across studies	15	Specify any assessment of risk of bias that may affect the cumulative evidence (e.g., publication bias, selective reporting within studies).	**7-8**
Additional analyses	16	Describe methods of additional analyses if done, indicating which were pre-specified. This may include, but not be limited to, the following:<br>• Sensitivity or subgroup analyses;<br>• Meta-regression analyses;<br>• *Alternative formulations of the treatment network; and*<br>• *Use of alternative prior distributions for Bayesian analyses (if applicable).*_	***8***
**RESULTS†**			
Study selection	17	Give numbers of studies screened, assessed for eligibility, and included in the review, with reasons for exclusions at each stage, ideally with a flow diagram.	**8**
**Presentation of network structure**	**S3**	Provide a network graph of the included studies to enable visualization of the geometry of the treatment network.	**8-9**
**Summary of network geometry**	**S4**	Provide a brief overview of characteristics of the treatment network. This may include commentary on the abundance of trials and randomized patients for the different interventions and pairwise comparisons in the network, gaps of evidence in the treatment network, and potential biases reflected by the	***9***

		network structure.	
Study characteristics	18	For each study, present characteristics for which data were extracted (e.g., study size, PICOS, follow-up period) and provide the citations.	**9**
Risk of bias within studies	19	Present data on risk of bias of each study and, if available, any outcome level assessment.	**9**
Results of individual studies	20	For all outcomes considered (benefits or harms), present, for each study: 1) simple summary data for each intervention group, and 2) effect estimates and confidence intervals. *Modified approaches may be needed to deal with information from larger networks.*	9
Synthesis of results	21	Present results of each meta-analysis done, including confidence/credible intervals. *In larger networks, authors may focus on comparisons versus a particular comparator (e.g. placebo or standard care), with full findings presented in an appendix. League tables and forest plots may be considered to summarize pairwise comparisons.* If additional summary measures were explored (such as treatment rankings), these should also be presented.	**9-13**
**Exploration for inconsistency**	**S5**	Describe results from investigations of inconsistency. This may include such information as measures of model fit to compare consistency and inconsistency models, *P* values from statistical tests, or summary of inconsistency estimates from different parts of the treatment network.	**9**
Risk of bias across studies	22	Present results of any assessment of risk of bias across studies for the evidence base being studied.	**9**
Results of additional analyses	23	Give results of additional analyses, if done (e.g., sensitivity or subgroup analyses, meta-regression analyses*, alternative network geometries studied, alternative choice of prior distributions for Bayesian analyses,* and so forth).	**14**
**DISCUSSION**			
Summary of evidence	24	Summarize the main findings, including the strength of evidence for each main outcome; consider their relevance to key groups (e.g., healthcare providers, users, and policy-makers).	**14-19**
Limitations	25	Discuss limitations at study and outcome level (e.g., risk of bias), and at review level (e.g., incomplete retrieval of identified research, reporting bias).	**19-20**

		Comment on the validity of the assumptions, such as transitivity and consistency. Comment on any concerns regarding network geometry (e.g., avoidance of certain comparisons).	
Conclusions	26	Provide a general interpretation of the results in the context of other evidence, and implications for future research.	**20**
**FUNDING**			
Funding	27	Describe sources of funding for the systematic review and other support (e.g., supply of data); role of funders for the systematic review. This should also include information regarding whether funding has been received from manufacturers of treatments in the network and/or whether some of the authors are content experts with professional conflicts of interest that could affect use of treatments in the network.	**21**

PICOS = population, intervention, comparators, outcomes, study design. *Italicized text denotes wording specific to network meta-analysis reporting added to guidance from the PRISMA statement. † Authors may utilize appendices to provide the full details pertaining to items included in this section.

eTable 2. Search strategy

**Search strategy for PubMed, EMBASE, and Cochrane Library databases**	
**PubMed**	((((((((((((((((((((((((((Immune Checkpoint Inhibitor[Title/Abstract]) OR (immune therapy[Title/Abstract])) OR (immunotherapy[Title/Abstract])) OR (nivolumab[Title/Abstract])) OR (cemiplimab[Title/Abstract])) OR (pembrolizumab[Title/Abstract])) OR (atezolizumab[Title/Abstract])) OR (durvalumab[Title/Abstract])) OR (tremelimumab[Title/Abstract])) OR (ipilimumab[Title/Abstract])) OR (avelumab[Title/Abstract])) OR (Tislelizumab[Title/Abstract])) OR (sugemalimab[Title/Abstract])) OR (Camrelizumab[Title/Abstract])) OR (sintilimab[Title/Abstract])) OR (Toripalimab[Title/Abstract])) OR (cytotoxic T-lymphocyte associated antigen-4[Title/Abstract])) OR (CTLA-4[Title/Abstract])) OR (programmed cell death protein-1[Title/Abstract])) OR (programmed cell death protein[Title/Abstract])) OR (PD-1[Title/Abstract])) OR (programmed cell death-Ligand 1[Title/Abstract])) OR (PD-L1[Title/Abstract])) AND ((((((((((cancer[Title/Abstract]) OR (carcinoma[Title/Abstract])) OR (neoplasm[Title/Abstract])) OR (leukemia[Title/Abstract])) OR (lymphoma[Title/Abstract])) OR (melanoma[Title/Abstract])) OR (malignancy[Title/Abstract])) OR (malignancies[Title/Abstract])) OR (tumor[Title/Abstract])) OR (tumors[Title/Abstract]))) AND (((((((((Randomized Controlled Trial[Title/Abstract] OR controlled clinical trial[Title/Abstract]) OR randomized[title/abstract]) OR randomised[title/abstract]) OR randomly[title/abstract]) OR trial[title/abstract]) OR phase[title/abstract]))))) AND (English[Language])) AND (((((((((compare[title/abstract] OR comparison[title/abstract]) OR comparative[title/abstract]) OR comparing[title/abstract]) OR versus[title/abstract]) OR vs[title/abstract])))))
**Web of science**	TS= (Immune Checkpoint Inhibitor OR immune therapy OR immunotherapy OR nivolumab OR cemiplimab OR pembrolizumab OR atezolizumab OR durvalumab OR tremelimumab OR ipilimumab OR avelumab OR Tislelizumab OR sugemalimab OR Camrelizumab OR sintilimab OR Toripalimab OR cytotoxic T-lymphocyte associated antigen-4 OR CTLA-4 OR programmed cell death protein-1 OR programmed cell death protein OR PD-1 OR programmed cell death-Ligand 1 OR PD-L1)<br>TS= (cancer OR carcinoma OR neoplasm OR leukemia OR lymphoma OR melanoma OR malignancy OR malignancies OR tumor OR tumors)<br>TS= (Randomized Controlled Trial OR controlled clinical trial OR randomized OR randomised OR randomly OR trial OR phase)<br>TS= (English)<br>TS= (compare OR comparison OR comparative OR comparing OR versus OR vs)

**Cochrane**	#1 ‘Immune Checkpoint Inhibitor’:ab,ti,kw OR ‘immune therapy’:ab,ti,kw OR ‘immunotherapy’:ab,ti,kw OR ‘nivolumab’:ab,ti,kw OR ‘cemiplimab’:ab,ti,kw OR ‘pembrolizumab’:ab,ti,kw OR ‘atezolizumab’:ab,ti,kw OR ‘durvalumab’:ab,ti,kw OR ‘tremelimumab’:ab,ti,kw OR ‘ipilimumab’:ab,ti,kw OR ‘avelumab’:ab,ti,kw OR ‘Tislelizumab’:ab,ti,kw OR ‘sugemalimab’:ab,ti,kw OR ‘Camrelizumab’:ab,ti,kw OR ‘sintilimab’:ab,ti,kw OR ‘Toripalimab’:ab,ti,kw OR ‘cytotoxic T-lymphocyte associated antigen-4’:ab,ti,kw OR ‘CTLA-4’:ab,ti,kw OR ‘programmed cell death protein-1’:ab,ti,kw OR ‘programmed cell death protein’:ab,ti,kw OR ‘PD-1’:ab,ti,kw OR ‘programmed cell death-Ligand 1’:ab,ti,kw OR ‘PD-L1’:ab,ti,kw<br>#2 ‘cancer’:ab,ti,kw OR ‘carcinoma’:ab,ti,kw OR ‘neoplasm’:ab,ti,kw OR ‘leukemia’:ab,ti,kw OR ‘lymphoma’:ab,ti,kw OR ‘melanoma’:ab,ti,kw OR ‘malignancy’:ab,ti,kw OR ‘malignancies’:ab,ti,kw OR ‘tumor’:ab,ti,kw OR ‘tumors’:ab,ti,kw<br>#3 ‘Randomized Controlled Trial’:ab,ti,kw OR ‘controlled clinical trial’:ab,ti,kw OR ‘randomized’:ab,ti,kw OR ‘randomised’:ab,ti,kw OR ‘randomly’:ab,ti,kw OR ‘trial’:ab,ti,kw OR ‘phase’:ab,ti,kw<br>#4 ‘English’:ab,ti,kw<br>#5 ‘compare’:ab,ti,kw OR ‘comparison’:ab,ti,kw OR ‘comparative’:ab,ti,kw OR ‘comparing’:ab,ti,kw OR ‘versus’:ab,ti,kw OR ‘vs’:ab,ti,kw<br>#1 and #2 and #3 and #4 and #5
**EMBASE**	#1 ‘Immune Checkpoint Inhibitor’:ab,ti OR ‘immune therapy’:ab,ti OR ‘immunotherapy’:ab,ti OR ‘nivolumab’:ab,ti OR ‘cemiplimab’:ab,ti OR ‘pembrolizumab’:ab,ti OR ‘atezolizumab’:ab,ti OR ‘durvalumab’:ab,ti OR ‘tremelimumab’:ab,ti OR ‘ipilimumab’:ab,ti OR ‘avelumab’:ab,ti OR ‘Tislelizumab’:ab,ti OR ‘sugemalimab’:ab,ti OR ‘Camrelizumab’:ab,ti OR ‘sintilimab’:ab,ti OR ‘Toripalimab’:ab,ti OR ‘cytotoxic T-lymphocyte associated antigen-4’:ab,ti OR ‘CTLA-4’:ab,ti OR ‘programmed cell death protein-1’:ab,ti OR ‘programmed cell death protein’:ab,ti OR ‘PD-1’:ab,ti OR ‘programmed cell death-Ligand 1’:ab,ti OR ‘PD-L1’:ab,ti<br>#2 ‘cancer’:ab,ti OR ‘carcinoma’:ab,ti OR ‘neoplasm’:ab,ti OR ‘leukemia’:ab,ti OR ‘lymphoma’:ab,ti OR ‘melanoma’:ab,ti OR ‘malignancy’:ab,ti OR ‘malignancies’:ab,ti OR ‘tumor’:ab,ti OR ‘tumors’:ab,ti<br>#3 ‘Randomized Controlled Trial’:ab,ti OR ‘controlled clinical trial’:ab,ti OR ‘randomized’:ab,ti OR ‘randomised’:ab,ti OR ‘randomly’:ab,ti OR ‘trial’:ab,ti OR ‘phase’:ab,ti<br>#4 ‘English’:ab,ti<br>#5 ‘compare’:ab,ti OR ‘comparison’:ab,ti OR ‘comparative’:ab,ti OR ‘comparing’:ab,ti OR ‘versus’:ab,ti OR ‘vs’:ab,ti

eTable 3. Network meta-analysis node-splitting analysis

	Direct effect		Indirect effect		
**Nodes**	**coefficient**	**SE**	**coefficient**	**SE**	**P**
**Any grade AEs**					
Ate, Ate_CT	2.84	0.91	2.38	1.72	0.831
Ate, CT	1.99	0.76	2.43	1.92	0.841
Ate_CT, CT	-0.65	0.35	-4.75	49.97	0.935
Ave_CT, CT	-2.59	1.16	2.80	439.83	0.99
CT, Cam_CT	1.64	0.69	-3.56	288.98	0.99
CT, Dur_CT	-0.09	0.59	-4.13	209.18	0.99
CT, Ipi_CT	-0.14	0.31	-4.12	73.40	0.96
CT, Niv_CT	0.87	0.62	-2.83	197.79	0.99
CT, Niv_Ipi	-0.89	0.57	-4.60	197.84	0.99
CT, Niv_Ipi_CT	0.47	0.57	-4.09	203.13	0.98
CT, Pem	-0.74	0.42	-0.14	0.99	0.59
CT, Pem_CT	0.25	0.26	-3.80	94.25	0.96
CT, Sin_CT	0.01	0.99	-4.13	551.10	0.99
CT, Sug_CT	1.42	0.88	-4.14	465.19	0.99
CT, Tis_CT	0.71	1.21	-4.46	587.34	0.99
CT, Tor_CT	0.02	1.51	-4.13	763.33	0.99
Pem, Pem_CT	0.80	0.39	1.40	1.03	0.59
**Grade 3 and above AEs**					
Ate, Ate_CT	2.32	0.40	2.79	0.84	0.62
Ate, CT	2.29	0.41	1.81	0.83	0.61
Ate_CT, CT	-0.21	0.19	-4.71	27.60	0.87
Ave_CT, CT	-0.21	0.38	4.39	138.26	0.97
CT, Cam_CT	0.34	0.25	-0.44	86.49	0.96
CT, Dur_CT	-0.04	0.38	-4.40	130.27	0.97
CT, Ipi_CT	0.37	0.21	-4.41	64.32	0.94
CT, Niv_CT	0.49	0.38	-4.13	130.26	0.97
CT, Niv_Ipi	-0.17	0.38	-4.80	130.25	0.97
CT, Niv_Ipi_CT	0.37	0.37	-4.40	127.91	0.97
CT, Pem	-1.57	0.23	-1.75	0.51	0.75
CT, Pem_CT	0.19	0.13	-4.03	44.82	0.93
CT, Sin_CT	0.19	0.30	-4.42	104.52	0.96
CT, Sug_CT	-0.01	0.39	-4.39	127.72	0.97
CT, Tis_CT	0.50	0.49	-4.39	178.26	0.97
CT, Tor_CT	-0.05	0.51	-4.40	204.39	0.98
Pem, Pem_CT	1.83	0.24	1.65	0.51	0.75

AE, Adverse effect; Ate, Atezolizumab; CT, Chemotherapy; Ate_CT, Atezolizumab + chemotherapy; Ave_CT, Avelumab + chemotherapy; Cam_CT, Camrelizumab + chemotherapy; Dur_CT, Durvalumab + chemotherapy; Ipi_CT, Ipilimumab + chemotherapy; Niv_CT, Nivolumab + chemotherapy; Niv_Ipi, Nivolumab + Ipilimumab ; Niv_Ipi_CT, Nivolumab + Ipilimumab + chemotherapy; Pem, Pembrolizumab; Pem_CT, Pembrolizumab + chemotherapy; Sin_CT, Sintilimab + chemotherapy; Sug_CT, Sugemalimab + chemotherapy; Tis_CT, Tislelizumab + chemotherapy; Tor_CT, Toripalimab + chemotherapy.

eFigure 1. Specific adverse event-based network maps

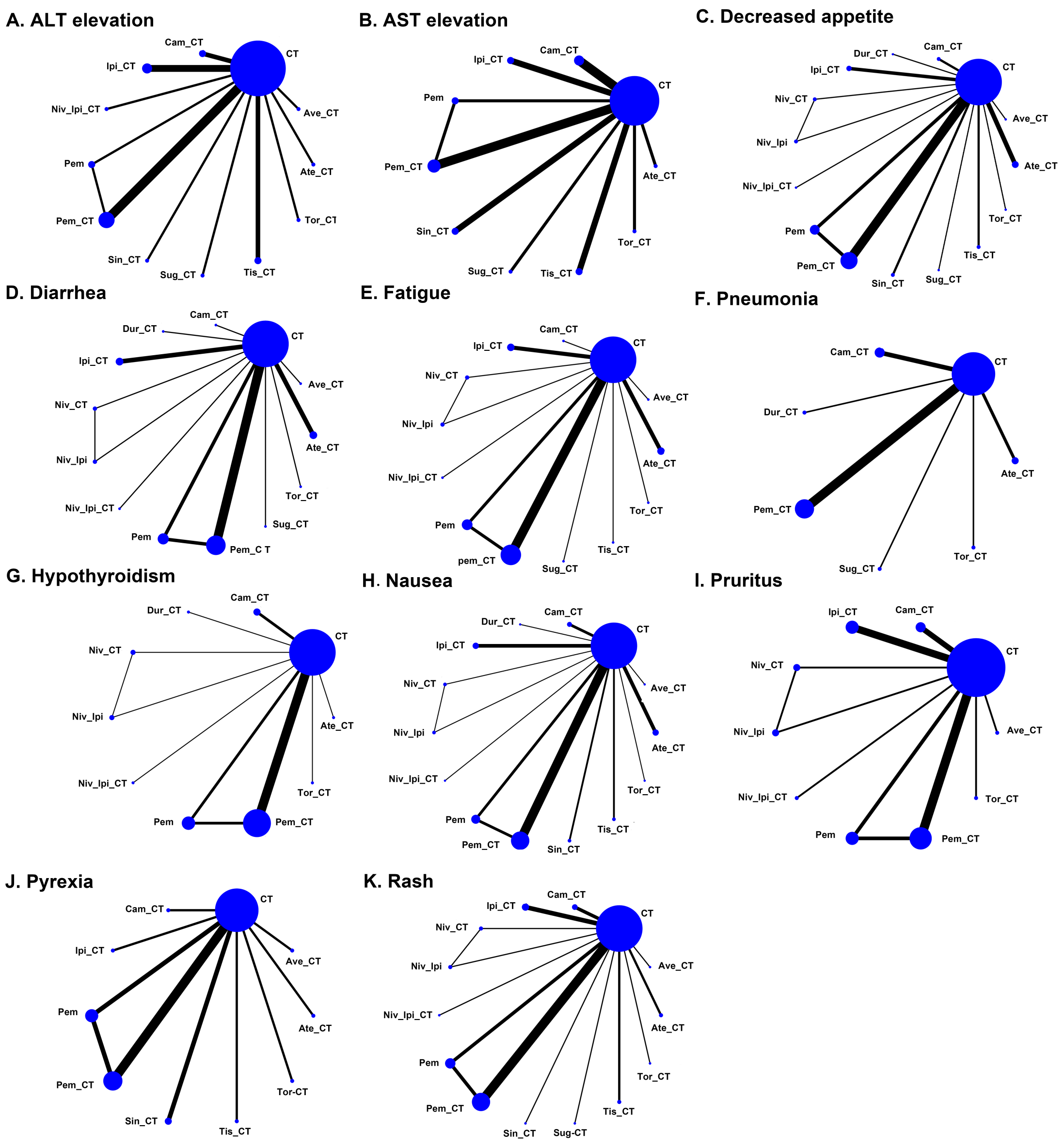

eFigure 2. Heterogeneity analyses

A. Any grade

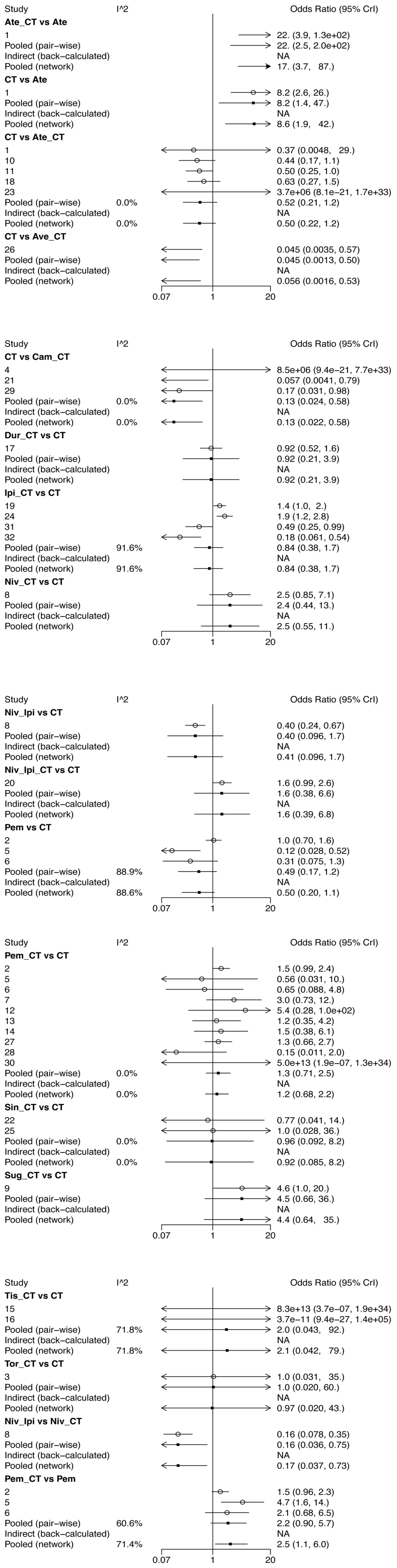

B. Grade 3 and above

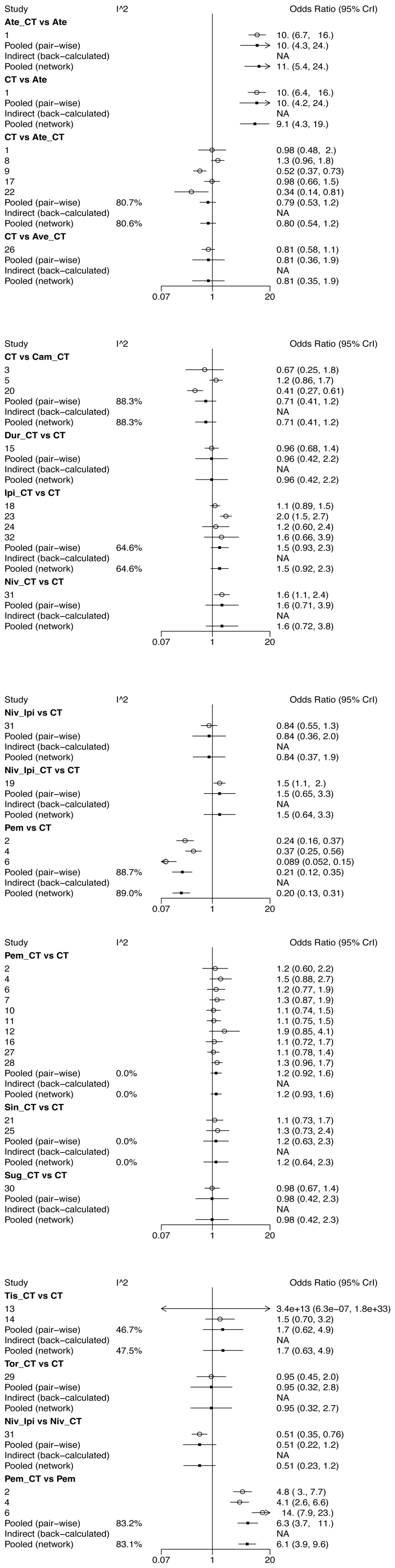

C. ALT elevation

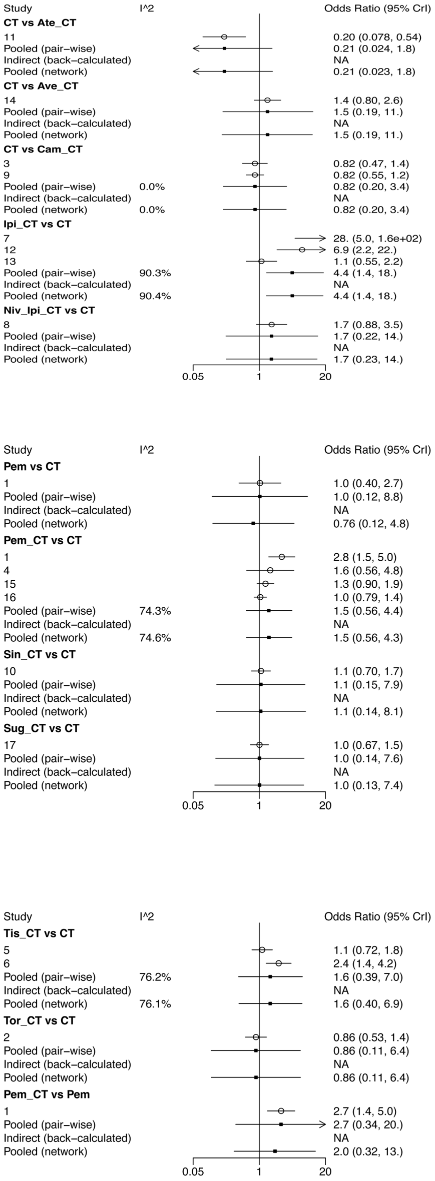

D. AST elevation

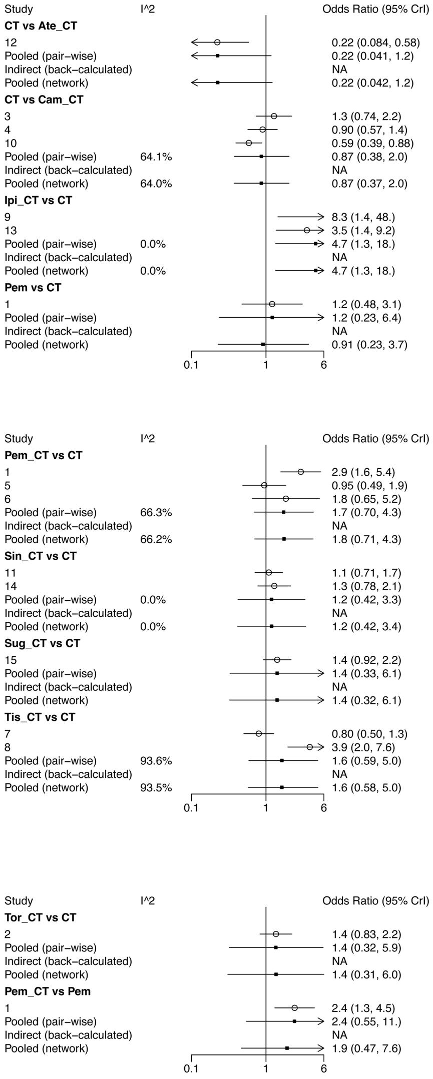

E. Decreased appetite

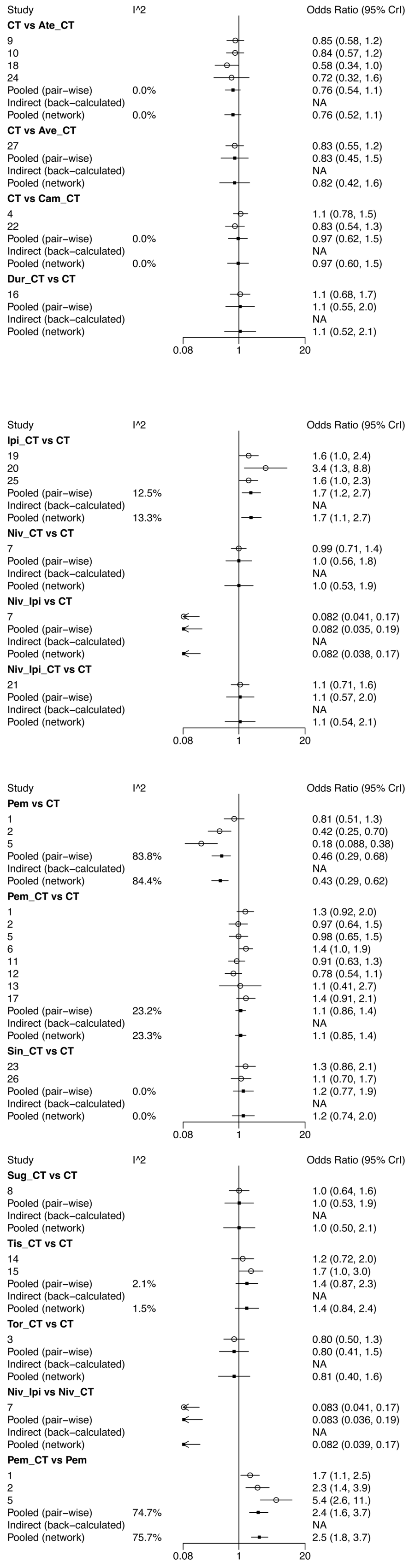

F. Diarrhea

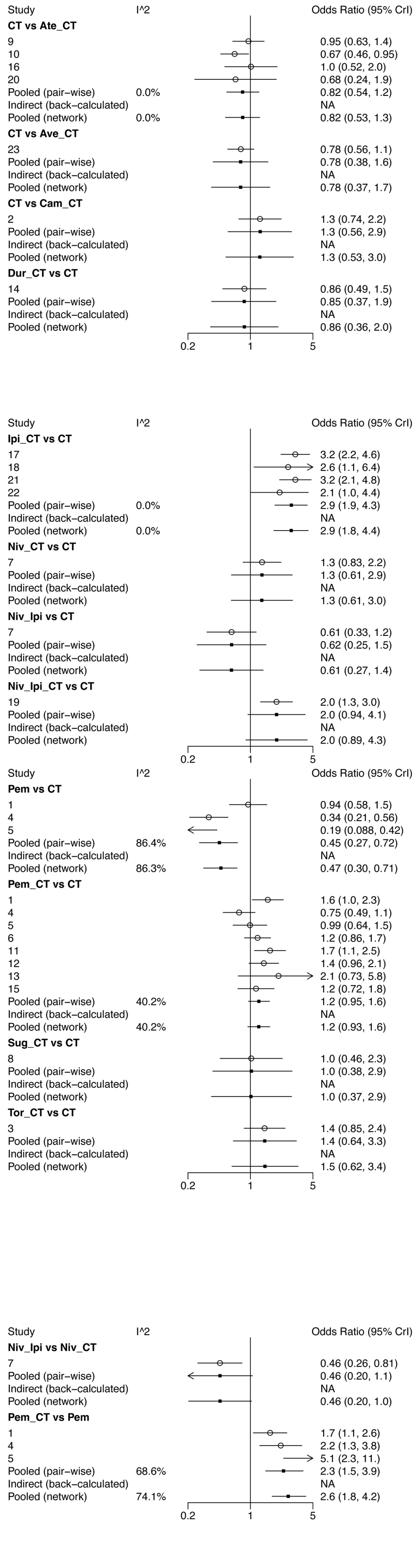

G. Fatigue

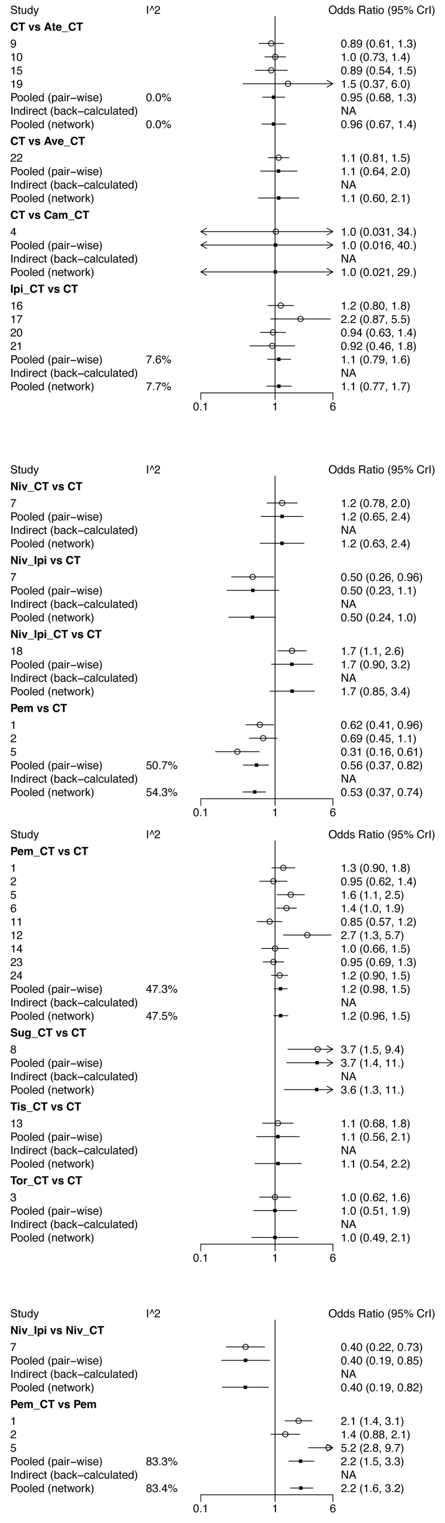

H. Nausea

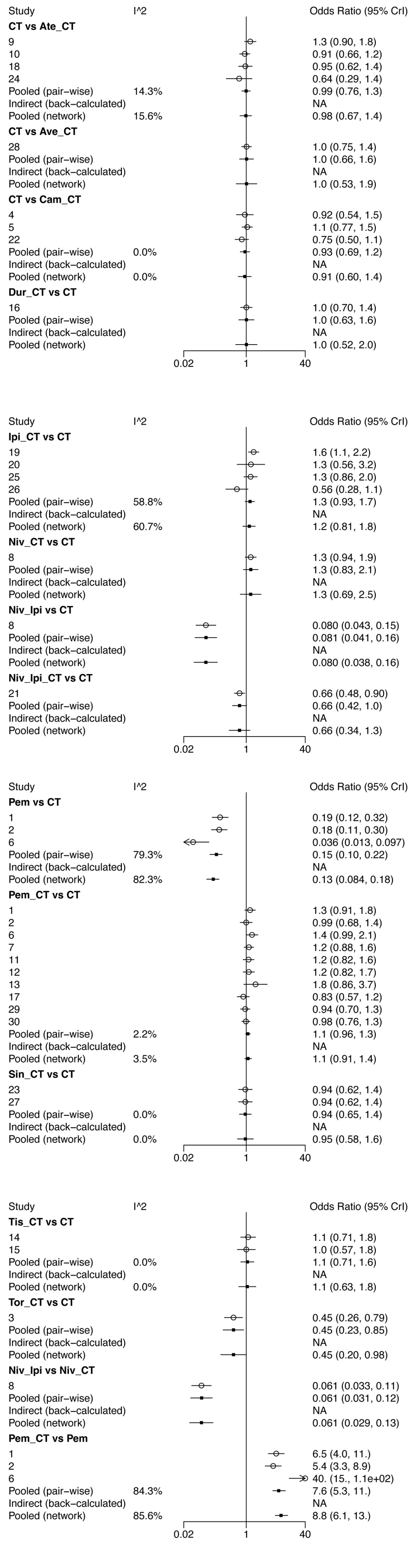

I. Hypothyroidism

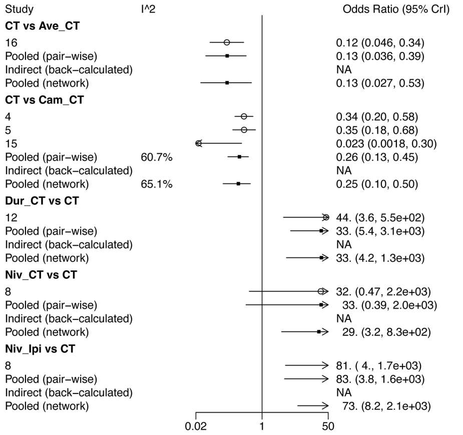

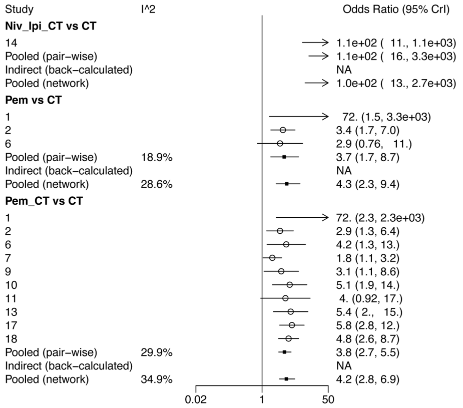

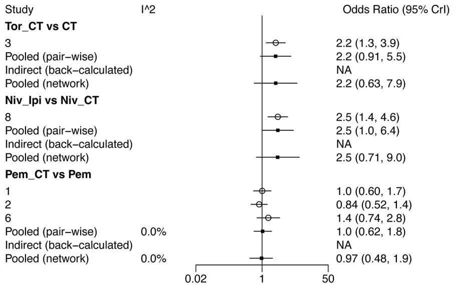

J. Pruritus

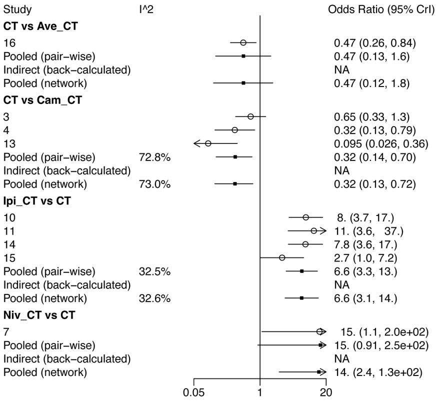

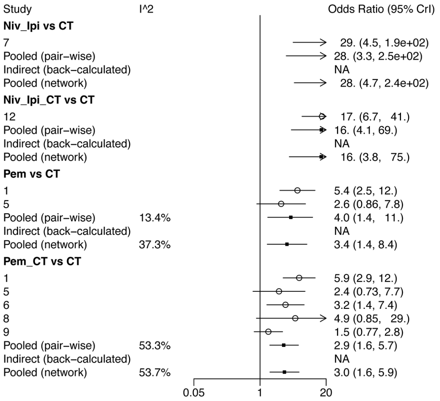

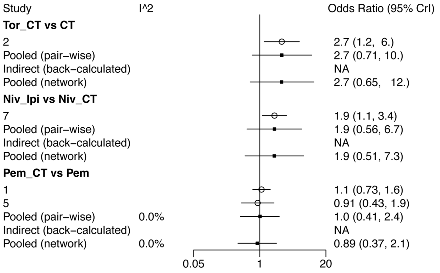

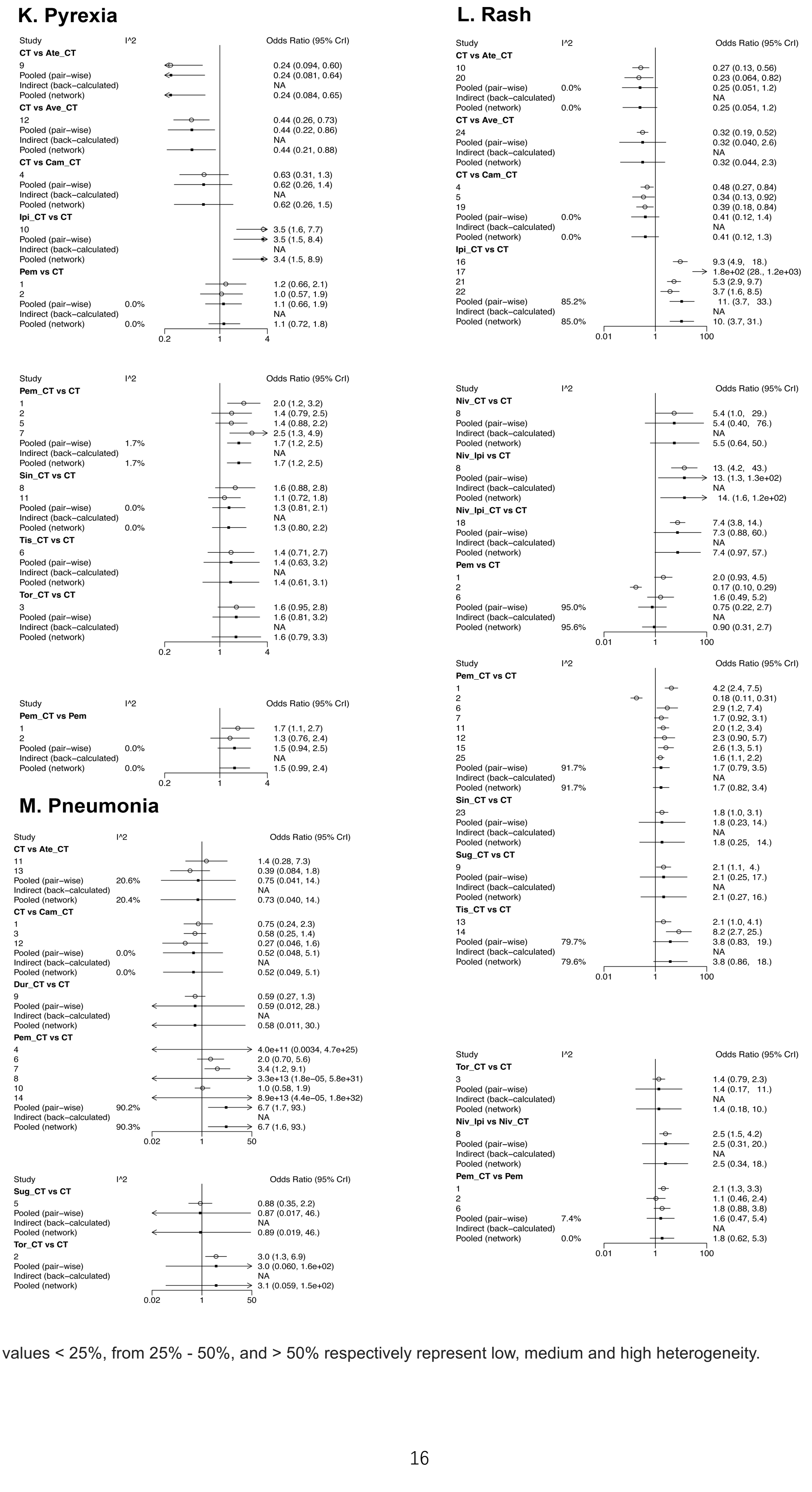

I^2 values < 25%, from 25% - 50%, and > 50% respectively represent low, medium and high heterogeneity.

eFigure 3. Inconsistency Analysis

A. Any grade

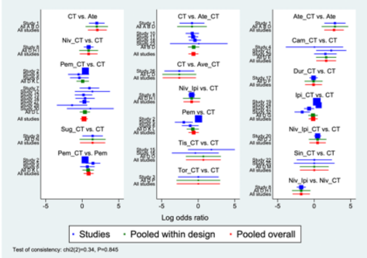

B. Grade 3 and above

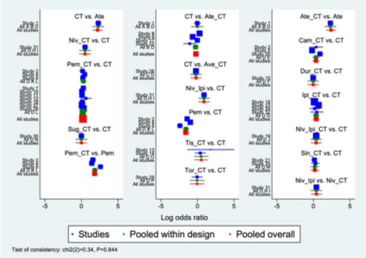

C. ALT elevation

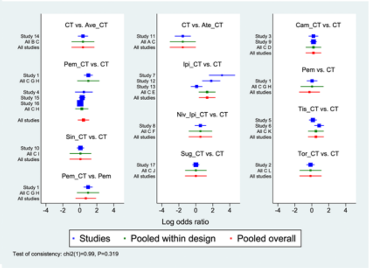

D. AST elevation

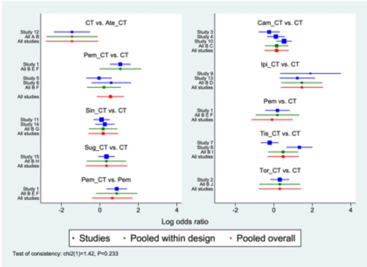

E. Decreased appetite

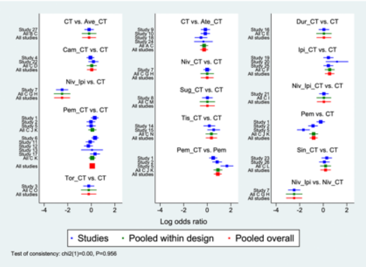

F. Diarrhea

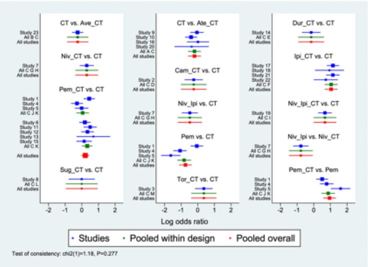

G. Nausea

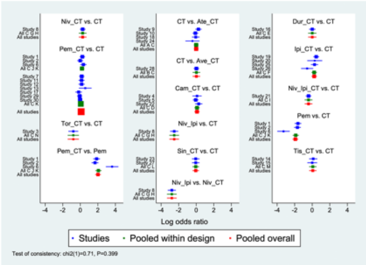

H. Fatigue

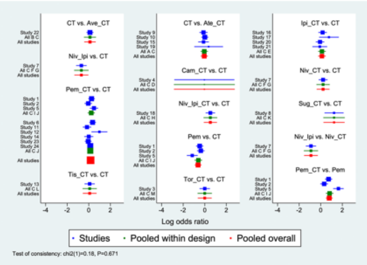

I. Pneumonia

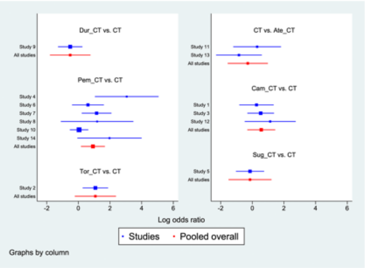

J. Hypothyroidism

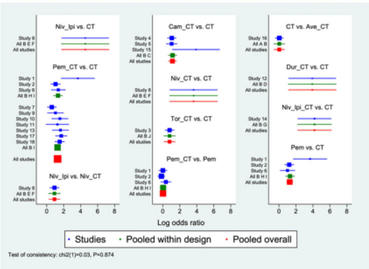

K. Pruritus

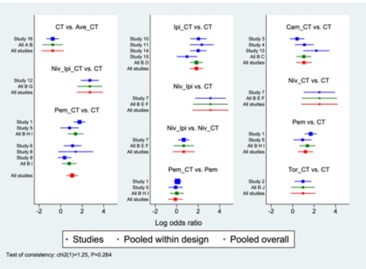

L. Rash

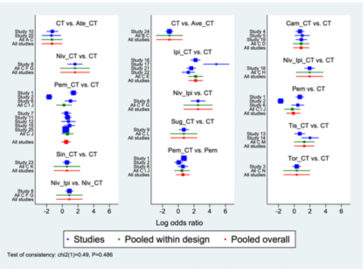

M. Pyrexia

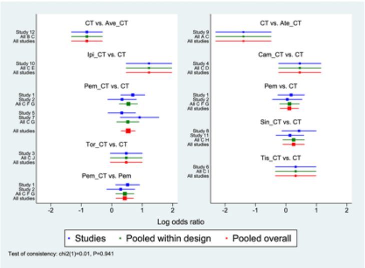

P > 0.05 denotes the absence of significant inconsistency.

eFigure 4. Direct comparisons of treatments based on the risk of adverse events

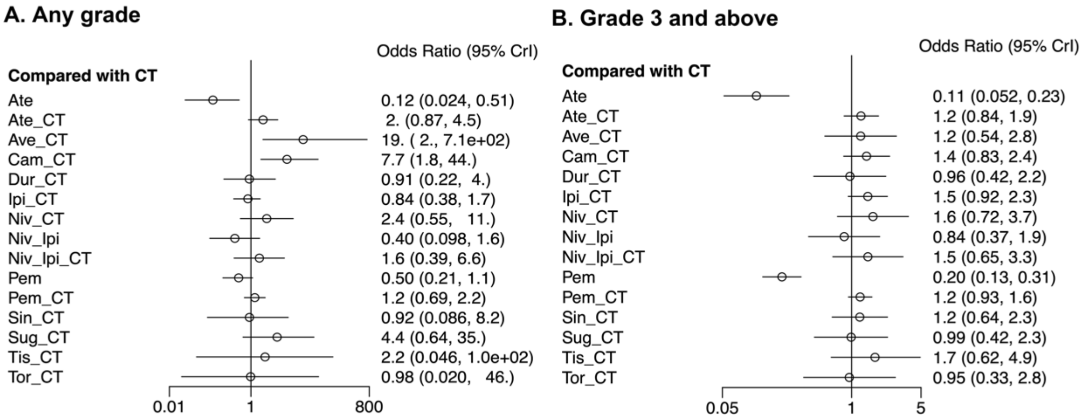

eFigure 5. Safety profiles based on specific adverse events

A. ALT elevated

Ate_CT	0.14 (0.01, 2.83)	0.25 (0.02, 3.3)	0.2 (0.02, 1.81)	0.91 (0.08, 12.77)	0.36 (0.02, 7.17)	0.16 (0.01, 2.77)	0.32 (0.03, 3.54)	0.22 (0.01, 4.27)	0.21 (0.01, 4.03)	0.34 (0.03, 4.51)	0.18 (0.01, 3.41)
6.99 (0.35, 135.76)	Ave_CT	1.75 (0.14, 20.81)	1.43 (0.18, 10.95)	6.37 (0.64, 77.2)	2.5 (0.14, 44.54)	1.09 (0.07, 17.31)	2.22 (0.23, 21.99)	1.56 (0.09, 27.22)	1.47 (0.08, 25.51)	2.36 (0.19, 28.84)	1.24 (0.07, 21.02)
4.02 (0.3, 53.38)	0.57 (0.05, 6.9)	Cam_CT	0.82 (0.2, 3.4)	3.62 (0.61, 27.62)	1.43 (0.12, 17.18)	0.62 (0.06, 6.52)	1.27 (0.22, 7.41)	0.89 (0.08, 10.56)	0.83 (0.07, 9.9)	1.35 (0.18, 10.3)	0.71 (0.06, 8.26)
4.89 (0.55, 41.7)	0.7 (0.09, 5.41)	1.22 (0.29, 5.08)	CT	**4.41 (1.38, 17.55)**	1.74 (0.22, 13.64)	0.76 (0.12, 4.93)	1.55 (0.56, 4.34)	1.08 (0.14, 7.98)	1.01 (0.14, 7.68)	1.64 (0.39, 6.9)	0.86 (0.12, 6.5)
1.1 (0.08, 12.33)	0.16 (0.01, 1.56)	0.28 (0.04, 1.65)	**0.23 (0.06, 0.73)**	Ipi_CT	0.39 (0.03, 3.97)	0.17 (0.02, 1.49)	0.35 (0.06, 1.63)	0.25 (0.02, 2.35)	0.23 (0.02, 2.18)	0.37 (0.05, 2.27)	0.19 (0.02, 1.89)
2.8 (0.14, 55.95)	0.4 (0.02, 7.13)	0.7 (0.06, 8.46)	0.57 (0.07, 4.46)	2.54 (0.25, 31.95)	Niv_Ipi_CT	0.44 (0.03, 7.22)	0.89 (0.09, 8.93)	0.62 (0.04, 11.23)	0.58 (0.03, 10.73)	0.94 (0.08, 11.33)	0.5 (0.03, 8.8)
6.4 (0.36, 107.07)	0.91 (0.06, 13.92)	1.6 (0.15, 15.88)	1.31 (0.2, 8.18)	5.79 (0.67, 60.31)	2.29 (0.14, 35.92)	Pem	2.04 (0.33, 12.69)	1.42 (0.09, 20.99)	1.34 (0.09, 20.18)	2.15 (0.21, 21.88)	1.14 (0.07, 17.09)
3.15 (0.28, 33.64)	0.45 (0.05, 4.32)	0.78 (0.14, 4.45)	0.65 (0.23, 1.77)	2.85 (0.61, 15.97)	1.12 (0.11, 10.8)	0.49 (0.08, 3.04)	Pem_CT	0.7 (0.07, 6.43)	0.66 (0.07, 6.16)	1.06 (0.18, 6.12)	0.56 (0.06, 5.14)
4.49 (0.23, 85.46)	0.64 (0.04, 11.2)	1.12 (0.09, 13.1)	0.92 (0.13, 6.95)	4.05 (0.42, 49.39)	1.6 (0.09, 28.21)	0.7 (0.05, 11.34)	1.43 (0.16, 14.05)	Sin_CT	0.94 (0.06, 16.27)	1.5 (0.13, 17.71)	0.8 (0.05, 13.39)
4.82 (0.25, 89.51)	0.68 (0.04, 12.02)	1.2 (0.1, 13.76)	0.99 (0.13, 7.12)	4.33 (0.46, 51.27)	1.72 (0.09, 30.31)	0.75 (0.05, 11.36)	1.52 (0.16, 14.43)	1.07 (0.06, 17.92)	Sug_CT	1.62 (0.14, 18.78)	0.85 (0.05, 14.61)
2.98 (0.22, 39.41)	0.42 (0.03, 5.17)	0.74 (0.1, 5.55)	0.61 (0.14, 2.55)	2.69 (0.44, 20.68)	1.06 (0.09, 12.91)	0.47 (0.05, 4.87)	0.95 (0.16, 5.56)	0.66 (0.06, 7.57)	0.62 (0.05, 7.26)	Tis_CT	0.53 (0.04, 6.24)
5.69 (0.29, 103.68)	0.81 (0.05, 14.08)	1.42 (0.12, 16.44)	1.16 (0.15, 8.69)	5.14 (0.53, 61.27)	2.02 (0.11, 35.47)	0.88 (0.06, 13.9)	1.79 (0.19, 17.51)	1.26 (0.07, 21.23)	1.18 (0.07, 20.38)	1.9 (0.16, 22.36)	Tor_CT

B. AST elevated

Ate_CT	0.26 (0.04, 1.71)	0.22 (0.04, 1.22)	1.04 (0.13, 9.31)	0.2 (0.02, 1.87)	0.39 (0.06, 2.61)	0.27 (0.04, 1.94)	0.32 (0.03, 2.97)	0.37 (0.05, 2.84)	0.3 (0.03, 2.89)
3.87 (0.58, 25.99)	Cam_CT	0.86 (0.37, 2.03)	4.02 (0.89, 20.35)	0.79 (0.15, 4.11)	1.52 (0.43, 5.25)	1.03 (0.27, 3.99)	1.22 (0.22, 6.84)	1.42 (0.38, 5.85)	1.17 (0.21, 6.68)
4.46 (0.82, 24.41)	1.16 (0.49, 2.73)	CT	**4.64 (1.34, 18.45)**	0.92 (0.22, 3.77)	1.76 (0.69, 4.36)	1.18 (0.42, 3.41)	1.41 (0.32, 6.26)	1.64 (0.58, 5)	1.36 (0.31, 6.06)
0.96 (0.11, 7.85)	0.25 (0.05, 1.12)	**0.22 (0.05, 0.75)**	Ipi_CT	0.2 (0.03, 1.27)	0.38 (0.07, 1.73)	0.25 (0.04, 1.29)	0.3 (0.04, 2.09)	0.35 (0.06, 1.89)	0.29 (0.04, 2.02)
4.88 (0.53, 44.51)	1.26 (0.24, 6.57)	1.09 (0.27, 4.51)	5.08 (0.79, 36.8)	Pem	1.92 (0.47, 7.64)	1.29 (0.22, 7.42)	1.54 (0.2, 12.09)	1.79 (0.32, 11.03)	1.48 (0.2, 11.64)
2.54 (0.38, 17.74)	0.66 (0.19, 2.32)	0.57 (0.23, 1.44)	2.64 (0.58, 14.13)	0.52 (0.13, 2.13)	Pem_CT	0.67 (0.17, 2.77)	0.8 (0.14, 4.73)	0.94 (0.24, 4.1)	0.77 (0.14, 4.56)
3.77 (0.52, 27.87)	0.98 (0.25, 3.72)	0.85 (0.29, 2.39)	3.93 (0.77, 22.32)	0.78 (0.13, 4.47)	1.48 (0.36, 5.9)	Sin_CT	1.19 (0.2, 7.24)	1.38 (0.32, 6.42)	1.15 (0.19, 7.03)
3.16 (0.34, 29.85)	0.82 (0.15, 4.54)	0.71 (0.16, 3.11)	3.3 (0.48, 25.47)	0.65 (0.08, 4.94)	1.25 (0.21, 6.94)	0.84 (0.14, 5.12)	Sug_CT	1.16 (0.19, 7.68)	0.96 (0.12, 8)
2.72 (0.35, 19.68)	0.7 (0.17, 2.66)	0.61 (0.2, 1.71)	2.82 (0.53, 15.93)	0.56 (0.09, 3.17)	1.07 (0.24, 4.2)	0.72 (0.16, 3.11)	0.86 (0.13, 5.21)	Tis_CT	0.83 (0.13, 4.97)
3.29 (0.35, 31.76)	0.85 (0.15, 4.69)	0.74 (0.16, 3.23)	3.42 (0.5, 26.01)	0.68 (0.09, 5.12)	1.3 (0.22, 7.3)	0.87 (0.14, 5.35)	1.04 (0.12, 8.51)	1.21 (0.2, 7.79)	Tor_CT

C. Decreased appetite

Ate_CT	0.92 (0.42, 1.98)	0.79 (0.43, 1.44)	0.76 (0.52, 1.09)	0.81 (0.36, 1.78)	1.31 (0.75, 2.39)	0.75 (0.36, 1.56)	**0.06 (0.03, 0.14)**	0.8 (0.37, 1.72)	**0.33 (0.19, 0.54)**	0.82 (0.53, 1.28)	0.93 (0.49, 1.7)	0.77 (0.35, 1.71)	1.09 (0.57, 2.05)	0.61 (0.27, 1.37)
1.09 (0.51, 2.37)	Ave_CT	0.86 (0.38, 1.96)	0.82 (0.42, 1.62)	0.87 (0.33, 2.32)	1.43 (0.65, 3.29)	0.82 (0.32, 2.07)	**0.07 (0.02, 0.19)**	0.87 (0.34, 2.26)	**0.36 (0.16, 0.76)**	0.9 (0.44, 1.83)	1.01 (0.43, 2.33)	0.84 (0.32, 2.23)	1.18 (0.5, 2.77)	0.66 (0.25, 1.78)
1.27 (0.7, 2.31)	1.16 (0.51, 2.64)	Cam_CT	0.96 (0.6, 1.53)	1.02 (0.43, 2.38)	1.67 (0.88, 3.23)	0.96 (0.43, 2.09)	**0.08 (0.03, 0.19)**	1.01 (0.44, 2.31)	**0.41 (0.22, 0.74)**	1.04 (0.61, 1.75)	1.17 (0.59, 2.3)	0.98 (0.42, 2.29)	1.37 (0.68, 2.78)	0.77 (0.33, 1.82)
1.32 (0.91, 1.92)	1.21 (0.62, 2.38)	1.04 (0.65, 1.67)	CT	1.06 (0.52, 2.16)	**1.73 (1.13, 2.77)**	0.99 (0.52, 1.87)	**0.08 (0.04, 0.17)**	1.06 (0.54, 2.08)	**0.43 (0.29, 0.61)**	1.09 (0.85, 1.38)	1.22 (0.74, 2.01)	1.02 (0.5, 2.07)	1.43 (0.84, 2.42)	0.8 (0.39, 1.65)
1.24 (0.56, 2.78)	1.14 (0.43, 3.04)	0.98 (0.42, 2.3)	0.94 (0.46, 1.91)	Dur_CT	1.63 (0.72, 3.83)	0.93 (0.36, 2.43)	**0.08 (0.03, 0.22)**	0.99 (0.37, 2.66)	**0.41 (0.18, 0.89)**	1.02 (0.48, 2.16)	1.15 (0.48, 2.74)	0.96 (0.35, 2.62)	1.35 (0.56, 3.26)	0.76 (0.28, 2.1)
0.76 (0.42, 1.33)	0.7 (0.3, 1.54)	0.6 (0.31, 1.13)	**0.58 (0.36, 0.89)**	0.61 (0.26, 1.39)	Ipi_CT	0.57 (0.26, 1.23)	**0.05 (0.02, 0.11)**	0.61 (0.26, 1.34)	**0.25 (0.13, 0.43)**	0.63 (0.37, 1.02)	0.7 (0.35, 1.35)	0.59 (0.25, 1.34)	0.82 (0.41, 1.62)	0.46 (0.19, 1.07)
1.33 (0.64, 2.77)	1.22 (0.48, 3.11)	1.05 (0.48, 2.31)	1.01 (0.53, 1.91)	1.07 (0.41, 2.75)	1.74 (0.82, 3.87)	Niv_CT	**0.08 (0.04, 0.17)**	1.06 (0.42, 2.69)	**0.43 (0.2, 0.88)**	1.09 (0.55, 2.15)	1.23 (0.55, 2.73)	1.03 (0.39, 2.65)	1.44 (0.63, 3.28)	0.83 (0.31, 2.1)
**16.01 (7.01, 38.02)**	**14.71 (5.39, 41.24)**	**12.63 (5.25, 31.32)**	**12.15 (5.77, 26.24)**	**12.9 (4.6, 36.71)**	**21.05 (8.99, 52.35)**	**12.06 (5.72, 26.02)**	Niv_Ipi	**12.82 (4.69, 35.71)**	**5.24 (2.24, 12.03)**	**13.18 (6, 29.43)**	**14.87 (5.98, 36.81)**	**12.41 (4.4, 35.28)**	**17.37 (6.96, 44.48)**	**9.73 (3.45, 28.12)**
1.25 (0.58, 2.69)	1.15 (0.44, 2.96)	0.99 (0.43, 2.25)	0.95 (0.48, 1.85)	1.01 (0.38, 2.68)	1.64 (0.75, 3.78)	0.94 (0.37, 2.37)	**0.08 (0.03, 0.21)**	Niv_Ipi_CT	**0.41 (0.18, 0.87)**	1.03 (0.5, 2.1)	1.16 (0.5, 2.67)	0.96 (0.36, 2.56)	1.36 (0.57, 3.17)	0.76 (0.28, 2.04)
**3.06 (1.84, 5.28)**	**2.81 (1.32, 6.22)**	**2.41 (1.35, 4.5)**	**2.32 (1.63, 3.43)**	**2.46 (1.12, 5.6)**	**4.03 (2.32, 7.5)**	**2.3 (1.13, 4.89)**	**0.19 (0.08, 0.45)**	**2.45 (1.15, 5.41)**	Pem	**2.51 (1.77, 3.72)**	**2.84 (1.55, 5.38)**	**2.36 (1.08, 5.35)**	**3.32 (1.77, 6.4)**	1.86 (0.85, 4.27)
1.22 (0.78, 1.9)	1.12 (0.55, 2.3)	0.96 (0.57, 1.63)	0.92 (0.72, 1.17)	0.98 (0.46, 2.06)	1.6 (0.98, 2.71)	0.91 (0.46, 1.81)	**0.08 (0.03, 0.17)**	0.97 (0.48, 2.01)	**0.4 (0.27, 0.57)**	Pem_CT	1.13 (0.65, 1.96)	0.94 (0.44, 2)	1.32 (0.74, 2.37)	0.74 (0.35, 1.59)
1.08 (0.59, 2.02)	0.99 (0.43, 2.3)	0.85 (0.44, 1.7)	0.82 (0.5, 1.35)	0.87 (0.37, 2.08)	1.42 (0.74, 2.83)	0.81 (0.37, 1.83)	**0.07 (0.03, 0.17)**	0.86 (0.37, 2)	**0.35 (0.19, 0.65)**	0.89 (0.51, 1.55)	Sin_CT	0.83 (0.35, 1.98)	1.17 (0.57, 2.43)	0.66 (0.27, 1.58)
1.3 (0.58, 2.89)	1.19 (0.45, 3.15)	1.02 (0.44, 2.4)	0.98 (0.48, 2)	1.04 (0.38, 2.83)	1.7 (0.75, 4.03)	0.98 (0.38, 2.53)	**0.08 (0.03, 0.23)**	1.04 (0.39, 2.76)	**0.42 (0.19, 0.92)**	1.07 (0.5, 2.25)	1.2 (0.5, 2.84)	Sug_CT	1.41 (0.58, 3.4)	0.79 (0.29, 2.17)
0.92 (0.49, 1.77)	0.85 (0.36, 2)	0.73 (0.36, 1.47)	0.7 (0.41, 1.19)	0.74 (0.31, 1.79)	1.21 (0.62, 2.45)	0.69 (0.3, 1.59)	**0.06 (0.02, 0.14)**	0.74 (0.32, 1.75)	**0.3 (0.16, 0.57)**	0.76 (0.42, 1.35)	0.86 (0.41, 1.74)	0.71 (0.29, 1.72)	Tis_CT	0.56 (0.23, 1.37)
1.64 (0.73, 3.68)	1.51 (0.56, 4.06)	1.29 (0.55, 3.07)	1.24 (0.61, 2.55)	1.32 (0.48, 3.62)	2.16 (0.94, 5.14)	1.24 (0.48, 3.21)	**0.1 (0.04, 0.29)**	1.31 (0.49, 3.53)	0.54 (0.23, 1.18)	1.35 (0.63, 2.88)	1.52 (0.63, 3.65)	1.27 (0.46, 3.46)	1.78 (0.73, 4.34)	Tor_CT

D. Diarrhea

Ate_CT	1.05 (0.44, 2.53)	0.64 (0.25, 1.67)	0.82 (0.53, 1.27)	0.7 (0.26, 1.85)	2.35 (1.26, 4.32)	1.1 (0.45, 2.73)	0.51 (0.2, 1.29)	1.6 (0.67, 3.94)	0.38 (0.2, 0.7)	1.02 (0.61, 1.73)	0.83 (0.28, 2.61)	1.19 (0.45, 3.18)
0.95 (0.4, 2.27)	Ave_CT	0.6 (0.19, 1.91)	0.78 (0.37, 1.65)	0.66 (0.21, 2.08)	2.23 (0.93, 5.3)	1.05 (0.36, 3.14)	0.48 (0.16, 1.47)	1.52 (0.52, 4.51)	0.36 (0.15, 0.86)	0.96 (0.44, 2.18)	0.79 (0.22, 2.9)	1.13 (0.36, 3.5)
1.57 (0.6, 4.08)	1.65 (0.52, 5.18)	Cam_CT	1.29 (0.54, 3.02)	1.1 (0.32, 3.73)	3.69 (1.4, 9.58)	1.74 (0.54, 5.53)	0.79 (0.24, 2.58)	2.53 (0.79, 8.07)	0.6 (0.23, 1.55)	1.6 (0.64, 3.95)	1.31 (0.34, 5.08)	1.86 (0.55, 6.5)
1.22 (0.79, 1.9)	1.29 (0.6, 2.72)	0.78 (0.33, 1.85)	CT	0.85 (0.35, 2.02)	2.86 (1.84, 4.41)	1.35 (0.62, 2.96)	0.62 (0.27, 1.41)	1.96 (0.9, 4.31)	0.47 (0.3, 0.71)	1.24 (0.94, 1.66)	1.01 (0.37, 2.93)	1.45 (0.62, 3.52)
1.43 (0.54, 3.84)	1.51 (0.48, 4.78)	0.91 (0.27, 3.16)	1.17 (0.5, 2.84)	Dur_CT	3.37 (1.26, 8.91)	1.58 (0.49, 5.13)	0.72 (0.22, 2.42)	2.3 (0.71, 7.55)	0.55 (0.21, 1.44)	1.46 (0.59, 3.69)	1.19 (0.31, 4.65)	1.69 (0.51, 6)
**0.43 (0.23, 0.79)**	0.45 (0.19, 1.08)	**0.27 (0.1, 0.72)**	**0.35 (0.23, 0.54)**	**0.3 (0.11, 0.79)**	Ipi_CT	0.47 (0.19, 1.17)	0.22 (0.09, 0.55)	0.68 (0.28, 1.7)	0.16 (0.09, 0.3)	0.43 (0.26, 0.74)	0.35 (0.12, 1.12)	0.5 (0.2, 1.37)
0.91 (0.37, 2.21)	0.95 (0.32, 2.82)	0.58 (0.18, 1.86)	0.74 (0.34, 1.62)	0.63 (0.19, 2.04)	2.13 (0.86, 5.16)	Niv_CT	0.46 (0.2, 1.03)	1.46 (0.48, 4.42)	0.35 (0.14, 0.83)	0.92 (0.4, 2.11)	0.75 (0.21, 2.81)	1.07 (0.34, 3.54)
1.97 (0.78, 5)	2.08 (0.68, 6.31)	1.26 (0.39, 4.11)	1.62 (0.71, 3.67)	1.38 (0.41, 4.55)	**4.63 (1.82, 11.67)**	2.18 (0.97, 4.93)	Niv_Ipi	3.17 (1.03, 9.87)	0.76 (0.3, 1.88)	2 (0.85, 4.79)	1.64 (0.44, 6.24)	2.35 (0.71, 7.52)
0.63 (0.25, 1.5)	0.66 (0.22, 1.93)	0.4 (0.12, 1.26)	0.51 (0.23, 1.11)	0.43 (0.13, 1.4)	1.46 (0.59, 3.53)	0.69 (0.23, 2.1)	0.32 (0.1, 0.97)	Niv_Ipi_CT	0.24 (0.1, 0.57)	0.63 (0.28, 1.46)	0.52 (0.14, 1.92)	0.74 (0.23, 2.32)
**2.61 (1.44, 4.88)**	**2.74 (1.17, 6.64)**	1.66 (0.65, 4.39)	**2.13 (1.42, 3.32)**	1.82 (0.69, 4.85)	**6.11 (3.35, 11.33)**	**2.88 (1.2, 7.16)**	1.32 (0.53, 3.37)	**4.17 (1.75, 10.37)**	Pem	2.64 (1.76, 4.15)	2.16 (0.72, 6.85)	3.1 (1.21, 8.35)
0.98 (0.58, 1.65)	1.04 (0.46, 2.29)	0.63 (0.25, 1.55)	0.81 (0.6, 1.06)	0.69 (0.27, 1.69)	**2.31 (1.35, 3.84)**	1.09 (0.47, 2.49)	0.5 (0.21, 1.18)	1.58 (0.69, 3.63)	**0.38 (0.24, 0.57)**	Pem_CT	0.81 (0.28, 2.43)	1.17 (0.47, 2.93)
1.21 (0.38, 3.63)	1.27 (0.34, 4.46)	0.77 (0.2, 2.92)	0.99 (0.34, 2.74)	0.84 (0.22, 3.2)	2.84 (0.89, 8.47)	1.33 (0.36, 4.84)	0.61 (0.16, 2.29)	1.94 (0.52, 6.97)	0.46 (0.15, 1.38)	1.23 (0.41, 3.52)	Sug_CT	1.43 (0.37, 5.4)
0.84 (0.31, 2.2)	0.89 (0.29, 2.79)	0.54 (0.15, 1.82)	0.69 (0.28, 1.62)	0.59 (0.17, 1.97)	1.98 (0.73, 5.09)	0.93 (0.28, 2.98)	0.43 (0.13, 1.4)	1.36 (0.43, 4.31)	**0.32 (0.12, 0.83)**	0.86 (0.34, 2.12)	0.7 (0.19, 2.73)	Tor_CT

E. Fatigue

Ate_CT	0.86 (0.41, 1.81)	0.91 (0.03, 30.88)	0.96 (0.66, 1.39)	1.08 (0.64, 1.87)	1.2 (0.55, 2.62)	0.48 (0.21, 1.08)	1.62 (0.73, 3.61)	0.51 (0.3, 0.85)	1.14 (0.75, 1.78)	3.49 (1.19, 11.7)	1.04 (0.46, 2.35)	0.95 (0.42, 2.18)
1.16 (0.55, 2.44)	Ave_CT	1.06 (0.03, 36.83)	1.12 (0.59, 2.13)	1.26 (0.6, 2.69)	1.39 (0.54, 3.58)	0.56 (0.21, 1.46)	1.89 (0.73, 4.87)	0.59 (0.28, 1.22)	1.33 (0.68, 2.65)	4.06 (1.24, 14.81)	1.21 (0.46, 3.19)	1.11 (0.42, 2.92)
1.1 (0.03, 38.89)	0.94 (0.03, 35.71)	Cam_CT	1.06 (0.03, 37.56)	1.2 (0.03, 43.32)	1.31 (0.04, 49.84)	0.52 (0.01, 20.23)	1.8 (0.05, 66.74)	0.56 (0.02, 20.09)	1.27 (0.04, 44.93)	3.91 (0.1, 165.05)	1.15 (0.03, 44.28)	1.06 (0.03, 38.68)
1.04 (0.72, 1.51)	0.89 (0.47, 1.7)	0.94 (0.03, 31.84)	CT	1.13 (0.77, 1.68)	1.25 (0.62, 2.48)	0.5 (0.24, 1.03)	1.69 (0.84, 3.43)	0.53 (0.37, 0.75)	1.19 (0.96, 1.5)	3.62 (1.33, 11.44)	1.08 (0.52, 2.22)	0.99 (0.48, 2.06)
0.93 (0.53, 1.57)	0.8 (0.37, 1.67)	0.83 (0.02, 29.23)	0.89 (0.6, 1.3)	Ipi_CT	1.11 (0.49, 2.41)	0.44 (0.19, 0.99)	1.5 (0.66, 3.32)	0.47 (0.27, 0.78)	1.06 (0.67, 1.64)	3.22 (1.09, 10.56)	0.96 (0.42, 2.16)	0.89 (0.38, 1.99)
0.84 (0.38, 1.82)	0.72 (0.28, 1.84)	0.76 (0.02, 26.51)	0.8 (0.4, 1.61)	0.9 (0.41, 2.03)	Niv_CT	0.4 (0.19, 0.82)	1.36 (0.51, 3.61)	0.43 (0.19, 0.92)	0.95 (0.47, 2)	2.92 (0.86, 11.13)	0.87 (0.32, 2.35)	0.8 (0.29, 2.17)
2.1 (0.92, 4.76)	1.8 (0.68, 4.78)	1.92 (0.05, 67.41)	2.02 (0.98, 4.2)	**2.27 (1.01, 5.29)**	**2.51 (1.22, 5.22)**	Niv_Ipi	3.41 (1.23, 9.48)	1.07 (0.47, 2.41)	2.4 (1.13, 5.2)	7.3 (2.13, 28.76)	2.19 (0.78, 6.07)	2 (0.71, 5.65)
0.62 (0.28, 1.36)	0.53 (0.21, 1.37)	0.55 (0.01, 20.11)	0.59 (0.29, 1.19)	0.66 (0.3, 1.51)	0.74 (0.28, 1.97)	**0.29 (0.11, 0.81)**	Niv_Ipi_CT	0.31 (0.14, 0.68)	0.7 (0.34, 1.49)	2.15 (0.62, 8.12)	0.64 (0.23, 1.76)	0.59 (0.21, 1.63)
**1.96 (1.18, 3.31)**	1.68 (0.82, 3.54)	1.77 (0.05, 60.63)	**1.88 (1.33, 2.71)**	**2.12 (1.28, 3.67)**	**2.34 (1.09, 5.14)**	0.93 (0.42, 2.11)	**3.18 (1.46, 7.09)**	Pem	2.24 (1.6, 3.26)	6.85 (2.38, 22.43)	2.04 (0.92, 4.62)	1.87 (0.83, 4.27)
0.87 (0.56, 1.34)	0.75 (0.38, 1.46)	0.79 (0.02, 27)	0.84 (0.67, 1.04)	0.95 (0.61, 1.49)	1.05 (0.5, 2.14)	**0.42 (0.19, 0.88)**	1.42 (0.67, 2.98)	**0.45 (0.31, 0.63)**	Pem_CT	3.05 (1.09, 9.77)	0.91 (0.42, 1.92)	0.84 (0.38, 1.78)
**0.29 (0.09, 0.84)**	**0.25 (0.07, 0.8)**	0.26 (0.01, 9.62)	**0.28 (0.09, 0.75)**	**0.31 (0.09, 0.92)**	0.34 (0.09, 1.16)	**0.14 (0.03, 0.47)**	0.47 (0.12, 1.6)	**0.15 (0.04, 0.42)**	**0.33 (0.1, 0.92)**	Sug_CT	0.3 (0.08, 1.03)	0.27 (0.07, 0.94)
0.96 (0.43, 2.16)	0.83 (0.31, 2.17)	0.87 (0.02, 30.54)	0.92 (0.45, 1.91)	1.04 (0.46, 2.39)	1.15 (0.43, 3.12)	0.46 (0.16, 1.29)	1.56 (0.57, 4.29)	0.49 (0.22, 1.09)	1.1 (0.52, 2.36)	3.36 (0.97, 12.81)	Tis_CT	0.92 (0.33, 2.58)
1.05 (0.46, 2.38)	0.9 (0.34, 2.39)	0.94 (0.03, 34.32)	1.01 (0.49, 2.1)	1.13 (0.5, 2.61)	1.26 (0.46, 3.45)	0.5 (0.18, 1.4)	1.7 (0.61, 4.75)	0.53 (0.23, 1.2)	1.2 (0.56, 2.6)	**3.67 (1.06, 13.86)**	1.09 (0.39, 3.06)	Tor_CT

F. Hypothyroidism

Ave_CT	0.52 (0.1, 2.93)	0.13 (0.03, 0.53)	4.85 (0.35, 197.3)	3.31 (0.24, 185.69)	8.35 (0.63, 467.42)	12.63 (0.9, 554.85)	0.55 (0.11, 2.8)	0.53 (0.11, 2.46)	0.28 (0.04, 1.89)
1.91 (0.34, 10.08)	Cam_CT	0.24 (0.1, 0.49)	9.01 (0.91, 313.97)	6.2 (0.63, 298.74)	15.73 (1.64, 745.07)	23.6 (2.33, 844.88)	1.04 (0.35, 2.88)	1.02 (0.39, 2.38)	0.55 (0.11, 2.19)
**7.84 (1.88, 36.3)**	**4.1 (2.02, 10.16)**	CT	37.28 (4.62, 1241.87)	25.59 (3.16, 1157.62)	64.83 (8.27, 2918.74)	97.1 (11.95, 3305.94)	4.29 (2.26, 9.13)	4.16 (2.79, 6.76)	2.23 (0.64, 7.81)
0.21 (0.01, 2.85)	0.11 (0, 1.09)	**0.03 (0, 0.22)**	Dur_CT	0.69 (0.01, 49.76)	1.75 (0.03, 125.06)	2.58 (0.04, 159.45)	0.11 (0, 1.08)	0.11 (0, 0.97)	0.06 (0, 0.7)
0.3 (0.01, 4.14)	0.16 (0, 1.6)	**0.04 (0, 0.32)**	1.45 (0.02, 81.72)	Niv_CT	2.53 (0.73, 9.09)	3.74 (0.05, 232.56)	0.17 (0, 1.57)	0.16 (0, 1.42)	0.08 (0, 1.01)
0.12 (0, 1.59)	**0.06 (0, 0.61)**	**0.02 (0, 0.12)**	0.57 (0.01, 31.66)	0.4 (0.11, 1.38)	Niv_Ipi	1.47 (0.02, 92.05)	0.07 (0, 0.6)	0.06 (0, 0.54)	0.03 (0, 0.39)
0.08 (0, 1.11)	**0.04 (0, 0.43)**	**0.01 (0, 0.08)**	0.39 (0.01, 24.68)	0.27 (0, 19.69)	0.68 (0.01, 49.47)	Niv_Ipi_CT	0.04 (0, 0.42)	0.04 (0, 0.38)	0.02 (0, 0.27)
1.83 (0.36, 9.38)	0.96 (0.35, 2.83)	**0.23 (0.11, 0.44)**	8.71 (0.92, 303.42)	5.95 (0.63, 279.37)	**15.06 (1.67, 693.22)**	**22.62 (2.37, 795.2)**	Pem	0.97 (0.49, 1.85)	0.52 (0.12, 2.05)
1.88 (0.41, 9)	0.98 (0.42, 2.58)	**0.24 (0.15, 0.36)**	**8.9 (1.03, 304.07)**	6.1 (0.7, 279.96)	**15.51 (1.85, 694.27)**	**23.22 (2.65, 792.87)**	1.03 (0.54, 2.04)	Pem_CT	0.54 (0.14, 1.93)
3.52 (0.53, 25.09)	1.83 (0.46, 8.93)	0.45 (0.13, 1.55)	**17.2 (1.43, 680.94)**	11.77 (0.99, 610.81)	**30 (2.59, 1510.82)**	**44.58 (3.72, 1866.76)**	1.92 (0.49, 8.45)	1.87 (0.52, 7.32)	Tor_CT

G. Nausea

Ate_CT	0.96 (0.45, 1.96)	1.07 (0.62, 1.82)	0.98 (0.68, 1.39)	0.98 (0.46, 2.07)	1.19 (0.68, 1.94)	1.28 (0.6, 2.68)	0.08 (0.03, 0.17)	0.65 (0.3, 1.33)	0.12 (0.07, 0.2)	1.09 (0.72, 1.64)	0.92 (0.5, 1.68)	1.04 (0.54, 1.97)	0.44 (0.18, 1.03)
1.04 (0.51, 2.2)	Ave_CT	1.12 (0.53, 2.38)	1.02 (0.54, 1.94)	1.03 (0.41, 2.6)	1.24 (0.58, 2.56)	1.35 (0.54, 3.37)	0.08 (0.03, 0.21)	0.67 (0.27, 1.66)	0.13 (0.06, 0.27)	1.14 (0.59, 2.25)	0.96 (0.43, 2.19)	1.09 (0.48, 2.52)	0.46 (0.17, 1.25)
0.93 (0.55, 1.6)	0.89 (0.42, 1.89)	Cam_CT	0.91 (0.61, 1.36)	0.92 (0.42, 1.98)	1.1 (0.62, 1.89)	1.2 (0.56, 2.56)	0.07 (0.03, 0.16)	0.6 (0.28, 1.28)	0.12 (0.06, 0.2)	1.01 (0.65, 1.6)	0.86 (0.46, 1.63)	0.97 (0.5, 1.91)	0.41 (0.17, 0.98)
1.02 (0.72, 1.48)	0.98 (0.52, 1.85)	1.1 (0.74, 1.65)	CT	1.01 (0.52, 1.96)	1.21 (0.81, 1.75)	1.32 (0.69, 2.53)	0.08 (0.04, 0.16)	0.66 (0.34, 1.26)	0.13 (0.08, 0.18)	1.11 (0.91, 1.38)	0.94 (0.58, 1.56)	1.07 (0.62, 1.82)	0.45 (0.2, 0.98)
1.02 (0.48, 2.19)	0.98 (0.39, 2.45)	1.09 (0.51, 2.39)	0.99 (0.51, 1.93)	Dur_CT	1.2 (0.55, 2.54)	1.31 (0.52, 3.31)	0.08 (0.03, 0.21)	0.65 (0.26, 1.65)	0.13 (0.06, 0.26)	1.11 (0.55, 2.23)	0.94 (0.41, 2.17)	1.06 (0.45, 2.49)	0.44 (0.16, 1.25)
0.84 (0.51, 1.47)	0.81 (0.39, 1.73)	0.91 (0.53, 1.62)	0.82 (0.57, 1.23)	0.83 (0.39, 1.82)	Ipi_CT	1.08 (0.52, 2.35)	0.07 (0.03, 0.15)	0.54 (0.26, 1.18)	0.11 (0.06, 0.18)	0.92 (0.61, 1.45)	0.78 (0.43, 1.49)	0.88 (0.47, 1.73)	0.37 (0.15, 0.9)
0.78 (0.37, 1.66)	0.74 (0.3, 1.86)	0.83 (0.39, 1.8)	0.76 (0.4, 1.46)	0.76 (0.3, 1.94)	0.93 (0.43, 1.92)	Niv_CT	0.06 (0.03, 0.12)	0.5 (0.2, 1.26)	0.1 (0.04, 0.2)	0.85 (0.43, 1.69)	0.72 (0.32, 1.64)	0.81 (0.35, 1.87)	0.34 (0.12, 0.95)
**12.87 (5.8, 29.5)**	**12.25 (4.69, 32.55)**	**13.83 (6.09, 31.74)**	**12.56 (6.11, 26.15)**	**12.64 (4.72, 34.01)**	**15.26 (6.62, 34.31)**	**16.52 (8.04, 34.49)**	Niv_Ipi	8.29 (3.11, 22.11)	1.6 (0.69, 3.56)	14 (6.66, 30.17)	11.87 (4.95, 28.91)	13.36 (5.5, 33.22)	5.62 (1.93, 16.6)
1.55 (0.75, 3.33)	1.49 (0.6, 3.7)	1.67 (0.78, 3.61)	1.52 (0.79, 2.93)	1.53 (0.61, 3.88)	1.85 (0.85, 3.83)	2 (0.8, 5.07)	**0.12 (0.05, 0.32)**	Niv_Ipi_CT	0.19 (0.09, 0.4)	1.69 (0.86, 3.39)	1.44 (0.64, 3.28)	1.62 (0.7, 3.78)	0.68 (0.25, 1.9)
**8.03 (4.95, 14.1)**	**7.69 (3.77, 16.67)**	**8.63 (5.12, 15.48)**	**7.84 (5.52, 11.83)**	**7.92 (3.79, 17.56)**	**9.5 (5.61, 16.36)**	**10.32 (5, 22.75)**	0.63 (0.28, 1.45)	**5.16 (2.5, 11.3)**	Pem	8.75 (6.17, 13.25)	7.45 (4.09, 14.32)	8.39 (4.45, 16.66)	3.52 (1.49, 8.59)
0.92 (0.61, 1.4)	0.88 (0.44, 1.71)	0.99 (0.63, 1.54)	0.9 (0.72, 1.1)	0.9 (0.45, 1.81)	1.09 (0.69, 1.64)	1.18 (0.59, 2.33)	**0.07 (0.03, 0.15)**	0.59 (0.3, 1.17)	**0.11 (0.08, 0.16)**	Pem_CT	0.85 (0.49, 1.45)	0.96 (0.54, 1.7)	0.4 (0.18, 0.9)
1.08 (0.59, 2.01)	1.04 (0.46, 2.33)	1.16 (0.61, 2.19)	1.06 (0.64, 1.73)	1.06 (0.46, 2.43)	1.28 (0.67, 2.35)	1.39 (0.61, 3.15)	**0.08 (0.03, 0.2)**	0.7 (0.3, 1.57)	**0.13 (0.07, 0.24)**	1.18 (0.69, 2.02)	Sin_CT	1.13 (0.54, 2.34)	0.47 (0.19, 1.19)
0.96 (0.51, 1.84)	0.92 (0.4, 2.1)	1.03 (0.52, 2.01)	0.94 (0.55, 1.6)	0.94 (0.4, 2.22)	1.14 (0.58, 2.15)	1.23 (0.54, 2.86)	**0.07 (0.03, 0.18)**	0.62 (0.26, 1.43)	**0.12 (0.06, 0.22)**	1.04 (0.59, 1.86)	0.89 (0.43, 1.85)	Tis_CT	0.42 (0.16, 1.09)
2.28 (0.97, 5.49)	2.18 (0.8, 6.02)	**2.45 (1.02, 5.99)**	**2.24 (1.02, 4.92)**	2.25 (0.8, 6.3)	**2.71 (1.11, 6.46)**	**2.93 (1.06, 8.12)**	**0.18 (0.06, 0.52)**	1.47 (0.53, 4.06)	**0.28 (0.12, 0.67)**	**2.48 (1.11, 5.65)**	2.11 (0.84, 5.39)	2.38 (0.92, 6.23)	Tor_CT

H. Pneumonia

Ate_CT	1.44 (0.04, 58.98)	0.75 (0.04, 13.17)	0.44 (0, 55.59)	4.91 (0.27, 268.78)	0.65 (0.01, 86.14)	2.23 (0.02, 287.56)
0.7 (0.02, 25.73)	Cam_CT	0.52 (0.05, 5.03)	0.3 (0, 28.72)	3.33 (0.28, 118.4)	0.45 (0, 40.69)	1.56 (0.02, 136.39)
1.34 (0.08, 23.75)	1.94 (0.2, 20.02)	CT	0.58 (0.01, 29)	6.54 (1.6, 80.83)	0.88 (0.02, 42.14)	3.02 (0.06, 140.85)
2.3 (0.02, 274.59)	3.31 (0.03, 305.35)	1.71 (0.03, 80.95)	Dur_CT	10.95 (0.25, 1485.25)	1.5 (0.01, 364.1)	5.12 (0.02, 1171.8)
0.2 (0, 3.77)	0.3 (0.01, 3.55)	**0.15 (0.01, 0.63)**	0.09 (0, 4.06)	Pem_CT	0.14 (0, 6.01)	0.47 (0, 19.57)
1.54 (0.01, 192.95)	2.2 (0.02, 204.37)	1.14 (0.02, 54.74)	0.67 (0, 165.11)	7.33 (0.17, 1023.13)	Sug_CT	3.41 (0.01, 819.38)
0.45 (0, 55.26)	0.64 (0.01, 59.76)	0.33 (0.01, 15.75)	0.2 (0, 48.78)	2.13 (0.05, 299)	0.29 (0, 71.05)	Tor_CT

I. Pruritus

Ave_CT	1.46 (0.31, 7.39)	0.47 (0.12, 1.76)	3.13 (0.66, 14.43)	7.28 (0.76, 115.33)	14.02 (1.49, 217.06)	7.57 (1.05, 58.21)	1.57 (0.31, 7.9)	1.41 (0.31, 6.28)	1.26 (0.18, 9.11)
0.69 (0.14, 3.23)	Cam_CT	0.32 (0.13, 0.73)	2.14 (0.65, 6.36)	4.93 (0.65, 62.76)	9.51 (1.28, 119.96)	5.21 (0.92, 28.36)	1.08 (0.3, 3.62)	0.97 (0.31, 2.72)	0.86 (0.16, 4.45)
2.14 (0.57, 8.15)	**3.11 (1.38, 7.69)**	CT	6.66 (3.17, 14.08)	15.32 (2.5, 176.65)	29.46 (5, 337.83)	16.14 (3.79, 75)	3.36 (1.38, 8.29)	3.01 (1.56, 5.86)	2.68 (0.64, 11.49)
0.32 (0.07, 1.5)	0.47 (0.16, 1.54)	**0.15 (0.07, 0.32)**	Ipi_CT	2.31 (0.33, 30.1)	4.44 (0.65, 57.47)	2.43 (0.48, 13.37)	0.51 (0.16, 1.62)	0.45 (0.17, 1.23)	0.4 (0.08, 2.09)
0.14 (0.01, 1.32)	0.2 (0.02, 1.54)	**0.07 (0.01, 0.4)**	0.43 (0.03, 3.07)	Niv_CT	1.93 (0.51, 7.22)	1.05 (0.06, 11.27)	0.22 (0.02, 1.66)	0.2 (0.02, 1.36)	0.17 (0.01, 1.8)
**0.07 (0, 0.67)**	**0.11 (0.01, 0.78)**	**0.03 (0, 0.2)**	0.23 (0.02, 1.53)	0.52 (0.14, 1.96)	Niv_Ipi	0.55 (0.03, 5.58)	0.11 (0.01, 0.83)	0.1 (0.01, 0.68)	0.09 (0.01, 0.9)
**0.13 (0.02, 0.95)**	0.19 (0.04, 1.08)	**0.06 (0.01, 0.26)**	0.41 (0.07, 2.08)	0.95 (0.09, 16.12)	1.83 (0.18, 31.27)	Niv_Ipi_CT	0.21 (0.04, 1.16)	0.19 (0.03, 0.91)	0.16 (0.02, 1.28)
0.64 (0.13, 3.19)	0.93 (0.28, 3.33)	**0.3 (0.12, 0.72)**	1.98 (0.62, 6.28)	4.58 (0.6, 61.42)	**8.82 (1.2, 116.8)**	4.84 (0.86, 28.54)	Pem	0.9 (0.37, 2.13)	0.8 (0.15, 4.35)
0.71 (0.16, 3.19)	1.03 (0.37, 3.19)	**0.33 (0.17, 0.64)**	2.21 (0.81, 5.96)	5.09 (0.74, 64.5)	**9.79 (1.46, 122.89)**	**5.38 (1.1, 28.73)**	1.11 (0.47, 2.67)	Pem_CT	0.89 (0.18, 4.38)
0.8 (0.11, 5.67)	1.16 (0.22, 6.45)	0.37 (0.09, 1.57)	2.49 (0.48, 12.31)	5.81 (0.55, 96.79)	**11.23 (1.11, 183.85)**	6.06 (0.78, 48.72)	1.25 (0.23, 6.84)	1.12 (0.23, 5.48)	Tor_CT

J. Pyrexia

Ate_CT	0.55 (0.15, 1.84)	0.38 (0.1, 1.44)	0.24 (0.08, 0.66)	0.83 (0.21, 3.23)	0.27 (0.08, 0.81)	0.41 (0.13, 1.22)	0.31 (0.1, 0.96)	0.33 (0.09, 1.22)	0.39 (0.11, 1.35)
1.82 (0.54, 6.51)	Ave_CT	0.69 (0.23, 2.09)	0.44 (0.21, 0.88)	1.51 (0.49, 4.98)	0.5 (0.21, 1.13)	0.75 (0.34, 1.66)	0.57 (0.24, 1.35)	0.6 (0.21, 1.81)	0.7 (0.26, 1.92)
2.64 (0.69, 10.21)	1.45 (0.48, 4.29)	Cam_CT	0.64 (0.27, 1.46)	2.19 (0.65, 8.08)	0.72 (0.27, 1.8)	1.1 (0.43, 2.69)	0.83 (0.31, 2.19)	0.88 (0.26, 2.79)	1.02 (0.33, 3.06)
**4.18 (1.5, 12.11)**	**2.29 (1.13, 4.65)**	1.56 (0.68, 3.76)	CT	3.46 (1.45, 9.04)	1.13 (0.72, 1.74)	1.73 (1.24, 2.42)	1.3 (0.8, 2.14)	1.38 (0.6, 3.18)	1.6 (0.79, 3.28)
1.2 (0.31, 4.83)	0.66 (0.2, 2.03)	0.46 (0.12, 1.54)	**0.29 (0.11, 0.69)**	Ipi_CT	0.33 (0.11, 0.85)	0.5 (0.18, 1.29)	0.38 (0.13, 1.02)	0.4 (0.11, 1.29)	0.47 (0.14, 1.41)
**3.71 (1.23, 11.77)**	2.02 (0.89, 4.72)	1.38 (0.55, 3.71)	0.89 (0.57, 1.38)	**3.07 (1.17, 8.72)**	Pem	1.53 (1, 2.37)	1.15 (0.6, 2.24)	1.23 (0.48, 3.1)	1.42 (0.62, 3.28)
2.43 (0.82, 7.45)	1.33 (0.6, 2.91)	0.91 (0.37, 2.31)	**0.58 (0.41, 0.81)**	2 (0.78, 5.54)	0.65 (0.42, 1)	Pem_CT	0.75 (0.42, 1.37)	0.8 (0.33, 1.94)	0.93 (0.42, 2.04)
**3.2 (1.05, 10.24)**	1.76 (0.74, 4.11)	1.21 (0.46, 3.28)	0.77 (0.47, 1.25)	2.65 (0.98, 7.61)	0.87 (0.45, 1.66)	1.33 (0.73, 2.39)	Sin_CT	1.06 (0.4, 2.73)	1.24 (0.52, 2.91)
3.05 (0.82, 11.49)	1.66 (0.55, 4.85)	1.14 (0.36, 3.79)	0.73 (0.31, 1.66)	2.51 (0.78, 8.8)	0.81 (0.32, 2.07)	1.25 (0.52, 3.07)	0.94 (0.37, 2.49)	Tis_CT	1.16 (0.39, 3.46)
2.6 (0.74, 9.37)	1.43 (0.52, 3.86)	0.98 (0.33, 3.04)	0.63 (0.31, 1.27)	2.14 (0.71, 7.15)	0.71 (0.3, 1.62)	1.08 (0.49, 2.36)	0.81 (0.34, 1.94)	0.86 (0.29, 2.54)	Tor_CT

K. Rash

Ate_CT	0.79 (0.06, 9.5)	0.61 (0.09, 4.36)	0.25 (0.05, 1.16)	2.64 (0.42, 17.34)	1.35 (0.1, 19.24)	3.34 (0.24, 46.65)	1.85 (0.15, 23.67)	0.23 (0.04, 1.5)	0.42 (0.08, 2.28)	0.46 (0.04, 5.59)	0.53 (0.04, 6.43)	0.95 (0.11, 8.19)	0.35 (0.03, 4.31)
1.26 (0.11, 15.85)	Ave_CT	0.78 (0.08, 7.97)	0.32 (0.04, 2.32)	3.33 (0.36, 32.47)	1.7 (0.09, 32.62)	4.24 (0.23, 80.68)	2.35 (0.13, 41.54)	0.29 (0.03, 2.78)	0.53 (0.06, 4.39)	0.58 (0.03, 9.66)	0.67 (0.04, 11.5)	1.2 (0.1, 14.6)	0.44 (0.03, 7.43)
1.63 (0.23, 11.34)	1.28 (0.13, 13.26)	Cam_CT	0.41 (0.12, 1.37)	4.28 (0.88, 21.93)	2.18 (0.18, 27.17)	5.39 (0.48, 66.75)	2.99 (0.28, 32.5)	0.37 (0.07, 1.87)	0.68 (0.17, 2.76)	0.74 (0.07, 7.53)	0.85 (0.08, 8.9)	1.54 (0.23, 10.59)	0.56 (0.05, 5.78)
3.97 (0.86, 18.54)	3.14 (0.43, 22.93)	2.45 (0.73, 8.28)	CT	10.48 (3.74, 31.09)	5.33 (0.62, 48.51)	13.25 (1.62, 118.6)	7.37 (0.95, 57.5)	0.9 (0.32, 2.69)	1.66 (0.81, 3.42)	1.82 (0.25, 13.22)	2.09 (0.27, 16.01)	3.77 (0.86, 17.29)	1.37 (0.18, 10.19)
0.38 (0.06, 2.39)	0.3 (0.03, 2.76)	0.23 (0.05, 1.14)	**0.1 (0.03, 0.27)**	Ipi_CT	0.51 (0.04, 5.6)	1.26 (0.11, 13.76)	0.7 (0.07, 6.92)	0.09 (0.02, 0.38)	0.16 (0.04, 0.55)	0.17 (0.02, 1.61)	0.2 (0.02, 1.9)	0.36 (0.06, 2.22)	0.13 (0.01, 1.22)
0.74 (0.05, 10.47)	0.59 (0.03, 10.71)	0.46 (0.04, 5.42)	0.19 (0.02, 1.62)	1.96 (0.18, 22.33)	Niv_CT	2.48 (0.34, 18.55)	1.38 (0.07, 26.92)	0.17 (0.01, 1.89)	0.31 (0.03, 3.04)	0.34 (0.02, 6.43)	0.39 (0.02, 7.32)	0.71 (0.05, 9.97)	0.26 (0.01, 4.97)
0.3 (0.02, 4.1)	0.24 (0.01, 4.28)	0.19 (0.01, 2.1)	**0.08 (0.01, 0.62)**	0.79 (0.07, 8.71)	0.4 (0.05, 2.91)	Niv_Ipi	0.56 (0.03, 10.61)	0.07 (0.01, 0.75)	0.13 (0.01, 1.17)	0.14 (0.01, 2.51)	0.16 (0.01, 2.92)	0.29 (0.02, 3.85)	0.1 (0.01, 1.93)
0.54 (0.04, 6.86)	0.43 (0.02, 7.56)	0.33 (0.03, 3.62)	0.14 (0.02, 1.06)	1.42 (0.14, 14.64)	0.73 (0.04, 14.47)	1.8 (0.09, 35.81)	Niv_Ipi_CT	0.12 (0.01, 1.25)	0.23 (0.03, 2)	0.24 (0.01, 4.3)	0.28 (0.02, 5.09)	0.51 (0.04, 6.58)	0.19 (0.01, 3.25)
4.39 (0.67, 28.16)	3.47 (0.36, 32.77)	2.71 (0.54, 13.47)	1.11 (0.37, 3.17)	**11.55 (2.62, 52.61)**	5.89 (0.53, 67.7)	**14.62 (1.33, 165.47)**	8.15 (0.8, 82.54)	Pem	1.84 (0.63, 5.35)	2.01 (0.2, 19.29)	2.31 (0.23, 22.41)	4.19 (0.66, 26.06)	1.52 (0.16, 14.64)
2.39 (0.44, 12.85)	1.88 (0.23, 15.58)	1.47 (0.36, 5.99)	0.6 (0.29, 1.23)	**6.3 (1.81, 23.13)**	3.2 (0.33, 32.48)	7.97 (0.85, 79.06)	4.44 (0.5, 38.38)	0.54 (0.19, 1.6)	Pem_CT	1.09 (0.13, 9.08)	1.25 (0.14, 10.71)	2.27 (0.44, 11.93)	0.83 (0.1, 6.86)
2.18 (0.18, 27.67)	1.73 (0.1, 29.24)	1.35 (0.13, 13.93)	0.55 (0.08, 4.08)	5.77 (0.62, 57.38)	2.96 (0.16, 57.12)	7.31 (0.4, 143.2)	4.1 (0.23, 71.01)	0.5 (0.05, 4.96)	0.92 (0.11, 7.71)	Sin_CT	1.15 (0.07, 19.99)	2.09 (0.17, 25.93)	0.76 (0.05, 12.82)
1.9 (0.16, 24.47)	1.5 (0.09, 25.56)	1.17 (0.11, 12.61)	0.48 (0.06, 3.67)	4.99 (0.53, 51.65)	2.57 (0.14, 51.23)	6.4 (0.34, 124.09)	3.53 (0.2, 64.37)	0.43 (0.04, 4.4)	0.8 (0.09, 6.93)	0.87 (0.05, 14.98)	Sug_CT	1.81 (0.15, 22.63)	0.66 (0.04, 11.54)
1.05 (0.12, 8.8)	0.83 (0.07, 9.8)	0.65 (0.09, 4.41)	0.27 (0.06, 1.17)	2.78 (0.45, 17.51)	1.41 (0.1, 20.05)	3.5 (0.26, 48.46)	1.95 (0.15, 24.91)	0.24 (0.04, 1.51)	0.44 (0.08, 2.29)	0.48 (0.04, 5.84)	0.55 (0.04, 6.81)	Tis_CT	0.36 (0.03, 4.44)
2.89 (0.23, 36.31)	2.28 (0.13, 38.24)	1.79 (0.17, 18.62)	0.73 (0.1, 5.41)	7.65 (0.82, 76.29)	3.91 (0.2, 74.31)	9.66 (0.52, 185.35)	5.35 (0.31, 92.87)	0.66 (0.07, 6.44)	1.21 (0.15, 10.24)	1.32 (0.08, 21.86)	1.52 (0.09, 26.64)	2.74 (0.23, 34.36)	Tor_CT

eFigure 6. Specific adverse event-based ranking diagram

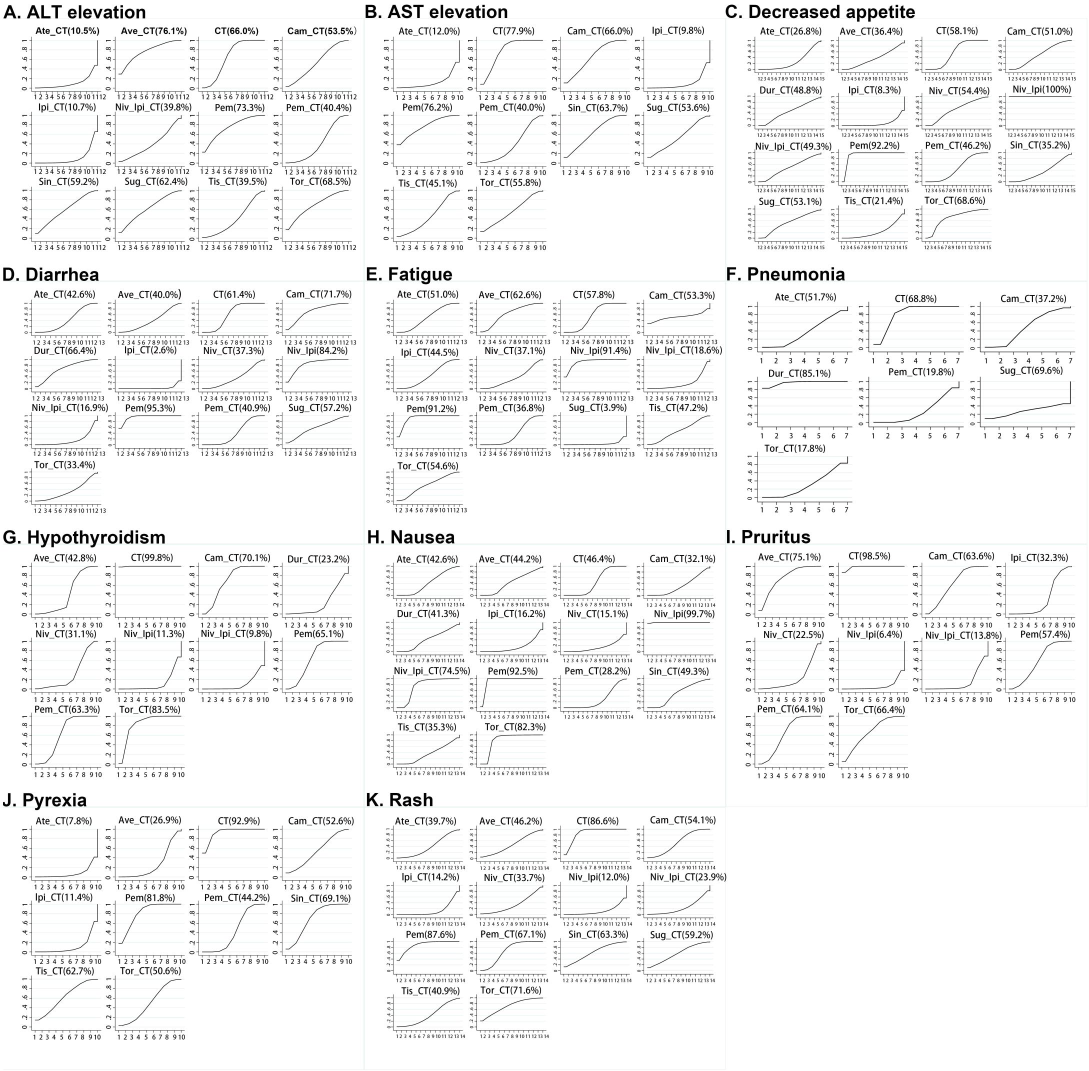

eFigure 7. Safety profiles based on specific grade 3 or higher adverse events

A. ALT elevated

Ate_CT	7.11 (0.05, 1659.52)	2.06 (0.02, 152.34)	0.95 (0.02, 50.47)	8.29 (0.11, 572.69)	2.71 (0.02, 272.98)	0.68 (0.01, 66.35)	1.79 (0.03, 109.18)	0.21 (0, 17.58)	0.74 (0.01, 84.1)	1.58 (0.02, 122.25)	0.89 (0, 233.31)
0.14 (0, 18.28)	Ave_CT	0.29 (0, 7.17)	0.14 (0, 2.02)	1.19 (0.02, 28.14)	0.37 (0, 15.7)	0.1 (0, 3.44)	0.27 (0, 4.75)	0.03 (0, 1.02)	0.1 (0, 4.65)	0.23 (0, 6.39)	0.11 (0, 15.13)
0.49 (0.01, 40.48)	3.41 (0.14, 219.56)	Cam_CT	0.46 (0.08, 2.47)	4.04 (0.38, 39.6)	1.3 (0.07, 28.17)	0.34 (0.02, 5.74)	0.87 (0.12, 6.63)	0.11 (0.01, 1.63)	0.35 (0.02, 8.83)	0.77 (0.07, 9.57)	0.42 (0.01, 28.61)
1.05 (0.02, 60.08)	7.06 (0.49, 318.92)	2.15 (0.4, 12.15)	CT	8.65 (1.81, 43.6)	2.75 (0.26, 37.81)	0.75 (0.06, 7.09)	1.88 (0.66, 5.67)	0.23 (0.02, 1.92)	0.76 (0.06, 11.81)	1.64 (0.31, 10.94)	0.9 (0.02, 47.73)
0.12 (0, 9.39)	0.84 (0.04, 53.05)	0.25 (0.03, 2.64)	**0.12 (0.02, 0.55)**	Ipi_CT	0.32 (0.02, 6.82)	0.08 (0, 1.43)	0.22 (0.03, 1.52)	0.53 (0, 1.09)	0.09 (0, 2.09)	0.19 (0.02, 2.36)	0.1 (0, 7.44)
0.37 (0, 42.44)	2.7 (0.06, 227.22)	0.77 (0.04, 14.44)	0.36 (0.03, 3.8)	3.14 (0.15, 51.69)	Niv_Ipi_CT	0.26 (0.01, 7.1)	0.69 (0.04, 9.22)	0.08 (0, 1.98)	0.27 (0.01, 9.65)	0.6 (0.03, 12.46)	0.32 (0, 33.84)
1.48 (0.02, 159.68)	10.43 (0.29, 967.66)	2.98 (0.17, 57.52)	1.34 (0.14, 16.32)	11.87 (0.7, 214.94)	3.91 (0.14, 130.76)	Pem	2.52 (0.28, 29.23)	0.32 (0.01, 8.07)	1.06 (0.04, 40.77)	2.27 (0.13, 49.09)	1.24 (0.01, 124.85)
0.56 (0.01, 35.89)	3.77 (0.21, 209.72)	1.15 (0.15, 8.47)	0.53 (0.18, 1.52)	4.58 (0.66, 31.86)	1.45 (0.11, 24.33)	0.4 (0.03, 3.63)	Pem_CT	0.12 (0.01, 1.29)	0.4 (0.03, 7.65)	0.87 (0.12, 7.58)	0.48 (0.01, 29.15)
4.68 (0.06, 503.31)	33.1 (0.98, 2658.31)	9.51 (0.61, 181.62)	4.31 (0.52, 49.19)	18.16 (0.53, 570.01)	12.4 (0.51, 402.71)	3.17 (0.12, 85.17)	8.25 (0.77, 115.13)	Sin_CT	3.46 (0.12, 126.82)	7.31 (0.47, 147.9)	3.96 (0.04, 402.62)
1.36 (0.01, 172.82)	9.78 (0.22, 928.64)	2.88 (0.11, 59.83)	1.32 (0.08, 15.84)	11.4 (0.48, 206.88)	3.66 (0.1, 130.32)	0.94 (0.02, 27.05)	2.49 (0.13, 37.2)	0.29 (0.01, 8.1)	Sug_CT	2.2 (0.09, 49.68)	1.2 (0.01, 118.67)
0.63 (0.01, 52.24)	4.43 (0.16, 291.93)	1.31 (0.1, 13.95)	0.61 (0.09, 3.27)	5.29 (0.42, 50.85)	1.68 (0.08, 35.18)	0.44 (0.02, 7.64)	1.15 (0.13, 8.48)	0.14 (0.01, 2.15)	0.46 (0.02, 11.11)	Tis_CT	0.53 (0.01, 42.23)
1.13 (0, 323.25)	8.81 (0.07, 2026.23)	2.38 (0.03, 182.71)	1.12 (0.02, 62.32)	9.72 (0.13, 726.6)	3.14 (0.03, 387.83)	0.8 (0.01, 79.5)	2.1 (0.03, 131.82)	0.25 (0, 22.49)	0.83 (0.01, 98.5)	1.88 (0.02, 143.92)	Tor_CT

B. AST elevated

Ate_CT	5.97 (0.14, 496.27)	2.27 (0.08, 137.81)	9.33 (0.21, 729.91)	1 (0.01, 164.08)	6.69 (0.14, 546.82)	1.65 (0.02, 211.26)	0.44 (0, 93.13)	4.67 (0.08, 519.36)	2.25 (0.02, 367.29)
0.17 (0, 7.29)	Cam_CT	0.39 (0.06, 2.04)	1.59 (0.13, 16.13)	0.17 (0, 5.86)	1.11 (0.08, 12.7)	0.28 (0.01, 6.98)	0.08 (0, 3.3)	0.78 (0.04, 15.98)	0.38 (0.01, 12.9)
0.44 (0.01, 12.72)	2.53 (0.49, 15.97)	CT	4.03 (0.74, 21.97)	0.45 (0.01, 10.01)	2.81 (0.48, 18.13)	0.7 (0.04, 11.45)	0.2 (0, 5.65)	1.99 (0.2, 25.42)	0.96 (0.04, 22.57)
0.11 (0, 4.83)	0.63 (0.06, 7.95)	0.25 (0.05, 1.36)	Ipi_CT	0.11 (0, 3.88)	0.69 (0.06, 8.67)	0.17 (0.01, 4.59)	0.05 (0, 2.26)	0.49 (0.03, 10.53)	0.23 (0.01, 8.92)
1 (0.01, 164.55)	5.86 (0.17, 413.95)	2.22 (0.1, 104.82)	9.19 (0.26, 580.81)	Pem	6.23 (0.34, 269.67)	1.63 (0.02, 170.96)	0.44 (0, 73.69)	4.61 (0.09, 439.57)	2.22 (0.03, 297.93)
0.15 (0, 7.19)	0.9 (0.08, 12.11)	0.36 (0.06, 2.09)	1.44 (0.12, 16.21)	0.16 (0, 2.92)	Pem_CT	0.24 (0.01, 6.85)	0.07 (0, 3.26)	0.71 (0.04, 15.98)	0.34 (0.01, 12.76)
0.61 (0, 50.47)	3.61 (0.14, 104.72)	1.43 (0.09, 22.82)	5.82 (0.22, 141.54)	0.61 (0.01, 42.92)	4.1 (0.15, 110.99)	Sin_CT	0.28 (0, 21.79)	2.87 (0.08, 124.55)	1.36 (0.02, 84.47)
2.27 (0.01, 424.24)	13.21 (0.3, 998.07)	5.05 (0.18, 264.18)	20.9 (0.44, 1446.29)	2.29 (0.01, 378.55)	14.6 (0.31, 1150.67)	3.62 (0.05, 464.23)	Sug_CT	10.53 (0.17, 1207.87)	5.09 (0.05, 719.09)
0.21 (0, 13.27)	1.28 (0.06, 24.61)	0.5 (0.04, 4.97)	2.03 (0.09, 34.2)	0.22 (0, 11.06)	1.41 (0.06, 26.78)	0.35 (0.01, 12.5)	0.09 (0, 5.82)	Tis_CT	0.48 (0.01, 24.18)
0.44 (0, 43.24)	2.67 (0.08, 97.86)	1.05 (0.04, 22.79)	4.27 (0.11, 140.03)	0.45 (0, 36.49)	2.96 (0.08, 106.21)	0.73 (0.01, 46.75)	0.2 (0, 18.71)	2.1 (0.04, 110.72)	Tor_CT

C. Decreased appetite

Ate_CT	1.29 (0.02, 69.91)	0.9 (0.15, 4.65)	1.32 (0.41, 3.7)	1.33 (0.07, 20.94)	3.47 (0.63, 18.44)	1.93 (0.24, 13.41)	0.65 (0.07, 4.99)	1.3 (0.12, 12.06)	0.53 (0.12, 2.21)	1.1 (0.28, 3.64)	0.6 (0.04, 6.56)	0.65 (0.01, 34.57)	1.88 (0.18, 23.99)	1.26 (0.02, 62.26)
0.77 (0.01, 42.36)	Ave_CT	0.69 (0.01, 39.43)	1 (0.02, 48.21)	1.01 (0.01, 103.98)	2.66 (0.05, 153.03)	1.43 (0.02, 92.67)	0.49 (0.01, 34.2)	0.98 (0.01, 76.3)	0.41 (0.01, 21.91)	0.83 (0.02, 41.95)	0.46 (0, 37.88)	0.48 (0, 127.17)	1.47 (0.02, 125.6)	0.95 (0, 206.17)
1.11 (0.22, 6.61)	1.44 (0.03, 84.29)	Cam_CT	1.45 (0.4, 5.57)	1.49 (0.08, 26.48)	3.85 (0.65, 24.85)	2.13 (0.26, 17.77)	0.72 (0.08, 6.57)	1.43 (0.13, 15.92)	0.59 (0.12, 3.07)	1.22 (0.28, 5.3)	0.66 (0.04, 8.61)	0.75 (0.01, 41.2)	2.1 (0.18, 28.48)	1.41 (0.02, 76.8)
0.76 (0.27, 2.43)	1 (0.02, 47.7)	0.69 (0.18, 2.49)	CT	1.01 (0.07, 13.66)	2.6 (0.76, 10.11)	1.45 (0.28, 7.83)	0.5 (0.08, 2.96)	0.98 (0.13, 7.25)	0.4 (0.15, 1.11)	0.83 (0.43, 1.61)	0.45 (0.04, 4.11)	0.49 (0.01, 23.59)	1.44 (0.18, 13.57)	0.96 (0.02, 43.04)
0.75 (0.05, 13.68)	0.99 (0.01, 104.61)	0.67 (0.04, 13.13)	0.99 (0.07, 13.98)	Dur_CT	2.65 (0.15, 53.11)	1.42 (0.07, 32.2)	0.49 (0.02, 11.87)	0.97 (0.04, 27.39)	0.4 (0.03, 6.94)	0.82 (0.06, 12.62)	0.45 (0.01, 13.31)	0.49 (0, 49.04)	1.43 (0.05, 43.37)	0.96 (0.01, 100.78)
0.29 (0.05, 1.58)	0.38 (0.01, 21.85)	0.26 (0.04, 1.55)	0.39 (0.1, 1.31)	0.38 (0.02, 6.71)	Ipi_CT	0.55 (0.07, 4.42)	0.19 (0.02, 1.62)	0.37 (0.03, 3.9)	**0.15 (0.03, 0.76)**	0.32 (0.07, 1.29)	0.17 (0.01, 2.17)	0.18 (0, 10.57)	0.55 (0.05, 6.84)	0.36 (0, 20.21)
0.52 (0.07, 4.12)	0.7 (0.01, 44.77)	0.47 (0.06, 3.88)	0.69 (0.13, 3.63)	0.7 (0.03, 14.67)	1.8 (0.23, 15.37)	Niv_CT	0.34 (0.06, 1.99)	0.67 (0.05, 8.95)	0.28 (0.04, 1.96)	0.57 (0.09, 3.39)	0.31 (0.02, 4.9)	0.34 (0, 22.53)	1 (0.07, 15.77)	0.66 (0.01, 40.96)
1.53 (0.2, 13.61)	2.06 (0.03, 142.34)	1.39 (0.15, 12.8)	2.01 (0.34, 12.52)	2.06 (0.08, 46.88)	5.29 (0.62, 51.45)	2.92 (0.5, 17.79)	Niv_Ipi	1.97 (0.13, 29.74)	0.82 (0.11, 6.55)	1.68 (0.24, 11.57)	0.93 (0.05, 15.41)	0.99 (0.01, 69.07)	2.93 (0.19, 52.22)	1.95 (0.02, 125.43)
0.77 (0.08, 8.31)	1.02 (0.01, 83.38)	0.7 (0.06, 7.84)	1.02 (0.14, 7.92)	1.03 (0.04, 27.91)	2.72 (0.26, 30.81)	1.49 (0.11, 21.02)	0.51 (0.03, 7.8)	Niv_Ipi_CT	0.41 (0.04, 4.08)	0.85 (0.1, 7.29)	0.46 (0.02, 9.28)	0.51 (0.01, 37.44)	1.49 (0.08, 29.73)	1 (0.01, 72.69)
1.87 (0.45, 8.59)	2.46 (0.05, 131.22)	1.7 (0.33, 8.44)	2.48 (0.9, 6.66)	2.51 (0.14, 39.73)	**6.47 (1.32, 34.14)**	3.57 (0.51, 24.8)	1.22 (0.15, 9.38)	2.42 (0.25, 22.44)	Pem	2.05 (0.74, 5.52)	1.11 (0.08, 13.2)	1.24 (0.02, 62.18)	3.57 (0.35, 41.86)	2.39 (0.04, 121.69)
0.91 (0.27, 3.53)	1.21 (0.02, 60.45)	0.82 (0.19, 3.53)	1.2 (0.62, 2.35)	1.22 (0.08, 17.5)	3.15 (0.78, 14.24)	1.75 (0.29, 10.82)	0.6 (0.09, 4.15)	1.18 (0.14, 9.88)	0.49 (0.18, 1.35)	Pem_CT	0.55 (0.04, 5.42)	0.6 (0.01, 29.62)	1.74 (0.2, 17.92)	1.16 (0.02, 55.08)
1.67 (0.15, 25.18)	2.19 (0.03, 213.79)	1.51 (0.12, 23.61)	2.2 (0.24, 25.04)	2.24 (0.08, 76.51)	5.83 (0.46, 89.49)	3.22 (0.2, 61.59)	1.07 (0.06, 22.14)	2.16 (0.11, 49.17)	0.9 (0.08, 12.25)	1.83 (0.18, 22.54)	Sin_CT	1.1 (0.01, 84.76)	3.27 (0.15, 79.99)	2.16 (0.02, 166.34)
1.54 (0.03, 99.25)	2.08 (0.01, 483.09)	1.34 (0.02, 96.13)	2.02 (0.04, 112.37)	2.03 (0.02, 214.71)	5.48 (0.09, 337.38)	2.91 (0.04, 216.69)	1.01 (0.01, 76.71)	1.96 (0.03, 170.11)	0.81 (0.02, 50.2)	1.67 (0.03, 96.83)	0.91 (0.01, 88.07)	Sug_CT	3.08 (0.04, 270.25)	1.99 (0.01, 496.27)
0.53 (0.04, 5.71)	0.68 (0.01, 57.23)	0.48 (0.04, 5.59)	0.7 (0.07, 5.54)	0.7 (0.02, 19.82)	1.83 (0.15, 21.93)	1 (0.06, 14.27)	0.34 (0.02, 5.37)	0.67 (0.03, 12.03)	0.28 (0.02, 2.84)	0.58 (0.06, 5.08)	0.31 (0.01, 6.87)	0.32 (0, 25.17)	Tis_CT	0.66 (0.01, 46.99)
0.79 (0.02, 55.01)	1.05 (0, 261.93)	0.71 (0.01, 49.15)	1.04 (0.02, 61.34)	1.04 (0.01, 140)	2.77 (0.05, 202.32)	1.5 (0.02, 134.22)	0.51 (0.01, 43.44)	1 (0.01, 95.18)	0.42 (0.01, 27.5)	0.86 (0.02, 53.81)	0.46 (0.01, 42.79)	0.5 (0, 146.76)	1.52 (0.02, 165.33)	Tor_CT

D. Diarrhea

Ate_CT	1.25 (0.02, 69.9)	0.93 (0.16, 4.49)	1.31 (0.42, 3.58)	1.26 (0.07, 21.6)	3.42 (0.67, 18.51)	1.92 (0.25, 12.7)	0.65 (0.07, 4.75)	1.27 (0.13, 11.56)	0.53 (0.12, 2.15)	1.09 (0.29, 3.53)	0.59 (0.04, 7.35)	0.67 (0.01, 40.69)	1.87 (0.17, 23.61)	1.23 (0.02, 50.02)
0.8 (0.01, 41.03)	Ave_CT	0.73 (0.01, 39.15)	1.03 (0.02, 51.52)	1.03 (0.01, 89.84)	2.77 (0.05, 133.1)	1.5 (0.02, 91.05)	0.5 (0.01, 32.91)	1.02 (0.01, 64.93)	0.42 (0.01, 21.16)	0.84 (0.02, 42.47)	0.47 (0, 39.03)	0.53 (0, 113.63)	1.45 (0.02, 102.08)	0.98 (0, 158.77)
1.07 (0.22, 6.36)	1.38 (0.03, 85.26)	Cam_CT	1.4 (0.4, 5.26)	1.38 (0.07, 26.71)	3.72 (0.66, 24.91)	2.07 (0.27, 17.3)	0.7 (0.08, 6.34)	1.38 (0.13, 15.72)	0.58 (0.12, 3.03)	1.16 (0.28, 5.07)	0.64 (0.04, 8.46)	0.73 (0.01, 49.95)	1.99 (0.18, 28.33)	1.34 (0.02, 61.93)
0.76 (0.28, 2.39)	0.97 (0.02, 48.55)	0.71 (0.19, 2.48)	CT	0.98 (0.06, 13.96)	2.61 (0.8, 10.36)	1.46 (0.29, 7.68)	0.5 (0.08, 2.85)	0.98 (0.13, 7.3)	0.41 (0.15, 1.11)	0.83 (0.43, 1.6)	0.45 (0.04, 4.77)	0.52 (0.01, 28.34)	1.41 (0.18, 13.91)	0.95 (0.02, 34.12)
0.79 (0.05, 15.17)	0.97 (0.01, 110.86)	0.72 (0.04, 13.92)	1.02 (0.07, 15.47)	Dur_CT	2.73 (0.14, 57.55)	1.49 (0.07, 34.43)	0.51 (0.02, 12.64)	0.99 (0.03, 29.18)	0.42 (0.02, 7.28)	0.85 (0.05, 13.84)	0.46 (0.01, 15.55)	0.55 (0, 59.18)	1.47 (0.05, 48.98)	0.99 (0.01, 81.36)
0.29 (0.05, 1.49)	0.36 (0.01, 20.85)	0.27 (0.04, 1.51)	0.38 (0.1, 1.24)	0.37 (0.02, 6.94)	Ipi_CT	0.56 (0.06, 4.16)	0.19 (0.02, 1.54)	0.37 (0.03, 3.78)	**0.16 (0.03, 0.72)**	0.32 (0.07, 1.22)	0.17 (0.01, 2.15)	0.19 (0, 12.17)	0.54 (0.05, 7.42)	0.36 (0.01, 15.37)
0.52 (0.08, 4.02)	0.66 (0.01, 44.16)	0.48 (0.06, 3.73)	0.69 (0.13, 3.5)	0.67 (0.03, 15.37)	1.79 (0.24, 15.47)	Niv_CT	0.34 (0.06, 1.93)	0.66 (0.05, 9.04)	0.28 (0.04, 1.96)	0.56 (0.1, 3.33)	0.3 (0.02, 5.09)	0.35 (0, 25.48)	0.98 (0.07, 16.32)	0.65 (0.01, 33.26)
1.54 (0.21, 13.48)	1.99 (0.03, 140.59)	1.44 (0.16, 12.64)	2.02 (0.35, 12.36)	1.97 (0.08, 49.93)	5.3 (0.65, 52.22)	2.97 (0.52, 17.55)	Niv_Ipi	1.97 (0.14, 30.44)	0.83 (0.11, 6.62)	1.67 (0.26, 11.53)	0.9 (0.05, 19.25)	1.04 (0.01, 83.41)	2.88 (0.19, 53.43)	1.91 (0.03, 105.26)
0.79 (0.09, 7.89)	0.98 (0.02, 77.03)	0.72 (0.06, 7.69)	1.02 (0.14, 7.46)	1.01 (0.03, 28.69)	2.72 (0.26, 29.85)	1.51 (0.11, 19.92)	0.51 (0.03, 7.16)	Niv_Ipi_CT	0.42 (0.05, 3.88)	0.85 (0.1, 6.94)	0.46 (0.02, 8.93)	0.53 (0.01, 40.56)	1.45 (0.08, 29.29)	0.95 (0.01, 59.54)
1.87 (0.46, 8.45)	2.36 (0.05, 130.39)	1.73 (0.33, 8.36)	2.43 (0.9, 6.51)	2.37 (0.14, 41.07)	**6.37 (1.39, 34.61)**	3.59 (0.51, 24.12)	1.21 (0.15, 8.94)	2.38 (0.26, 22.03)	Pem	2.02 (0.75, 5.43)	1.11 (0.08, 13.73)	1.26 (0.02, 74.4)	3.5 (0.34, 41.51)	2.29 (0.04, 94.32)
0.92 (0.28, 3.5)	1.18 (0.02, 60.77)	0.86 (0.2, 3.53)	1.21 (0.63, 2.34)	1.18 (0.07, 18.36)	3.17 (0.82, 14.25)	1.78 (0.3, 10.52)	0.6 (0.09, 3.92)	1.18 (0.14, 9.74)	0.5 (0.18, 1.34)	Pem_CT	0.54 (0.05, 6.27)	0.63 (0.01, 35.53)	1.72 (0.19, 18.98)	1.14 (0.02, 44.92)
1.68 (0.14, 24.27)	2.12 (0.03, 210.22)	1.55 (0.12, 22.99)	2.23 (0.21, 24.15)	2.19 (0.06, 75.32)	5.78 (0.47, 92.37)	3.28 (0.2, 57.24)	1.11 (0.05, 21.38)	2.15 (0.11, 46.99)	0.9 (0.07, 11.88)	1.84 (0.16, 21.56)	Sin_CT	1.14 (0.01, 115.09)	3.15 (0.15, 86.28)	2.07 (0.02, 157.91)
1.5 (0.02, 92.41)	1.88 (0.01, 442.2)	1.38 (0.02, 85.17)	1.94 (0.04, 101.89)	1.83 (0.02, 230.59)	5.22 (0.08, 349.55)	2.86 (0.04, 200.97)	0.96 (0.01, 74.31)	1.87 (0.02, 156.75)	0.79 (0.01, 47.65)	1.6 (0.03, 90.61)	0.87 (0.01, 82.07)	Sug_CT	2.78 (0.03, 265.24)	1.78 (0.01, 369.85)
0.54 (0.04, 5.92)	0.69 (0.01, 55.41)	0.5 (0.04, 5.58)	0.71 (0.07, 5.66)	0.68 (0.02, 19.19)	1.84 (0.13, 21.89)	1.02 (0.06, 14.83)	0.35 (0.02, 5.31)	0.69 (0.03, 12.45)	0.29 (0.02, 2.92)	0.58 (0.05, 5.14)	0.32 (0.01, 6.73)	0.36 (0, 32.34)	Tis_CT	0.67 (0.01, 42.53)
0.81 (0.02, 45.8)	1.02 (0.01, 248.55)	0.74 (0.02, 43.05)	1.05 (0.03, 50.62)	1.01 (0.01, 109)	2.79 (0.07, 172.56)	1.54 (0.03, 102.64)	0.52 (0.01, 36.53)	1.05 (0.02, 79.48)	0.44 (0.01, 23.22)	0.88 (0.02, 44.36)	0.48 (0.01, 55.27)	0.56 (0, 144.56)	1.49 (0.02, 129.37)	Tor_CT

E. Fatigue

Ate_CT	2.33 (0.54, 9.77)	1.12 (0.03, 35.63)	1.08 (0.53, 2.07)	1.52 (0.23, 10.32)	2.07 (0.78, 5.32)	0.63 (0.15, 2.47)	0.32 (0.07, 1.46)	4.75 (0.8, 40.31)	0.43 (0.16, 1.08)	1.44 (0.66, 3.1)	2.08 (0.18, 80.74)	0.78 (0.09, 8.4)	0.61 (0.06, 5.51)
0.43 (0.1, 1.87)	Ave_CT	0.5 (0.01, 19.6)	0.47 (0.13, 1.63)	0.65 (0.08, 5.62)	0.89 (0.2, 3.69)	0.27 (0.05, 1.56)	**0.14 (0.02, 0.89)**	2.1 (0.26, 23.12)	**0.18 (0.04, 0.77)**	0.62 (0.16, 2.3)	0.92 (0.07, 48.08)	0.34 (0.03, 4.66)	0.27 (0.02, 3.08)
0.9 (0.03, 30.41)	2 (0.05, 79.29)	Cam_CT	0.94 (0.03, 29.71)	1.35 (0.03, 62.83)	1.87 (0.06, 60.79)	0.56 (0.02, 22.17)	0.29 (0.01, 11.79)	4.16 (0.1, 258.71)	0.38 (0.01, 12.44)	1.26 (0.04, 40.81)	2.08 (0.03, 295.59)	0.73 (0.01, 44.74)	0.51 (0.01, 31.41)
0.93 (0.48, 1.9)	2.15 (0.62, 7.93)	1.06 (0.03, 32.72)	CT	1.37 (0.26, 8.6)	1.92 (1, 3.83)	0.59 (0.18, 1.97)	0.3 (0.07, 1.15)	4.44 (0.88, 33.93)	**0.4 (0.2, 0.77)**	1.34 (0.95, 1.94)	1.94 (0.19, 71.28)	0.74 (0.1, 7.05)	0.57 (0.06, 4.7)
0.66 (0.1, 4.41)	1.55 (0.18, 12.46)	0.74 (0.02, 34.08)	0.73 (0.12, 3.89)	Dur_CT	1.4 (0.2, 8.68)	0.41 (0.05, 3.53)	0.21 (0.02, 1.95)	3.29 (0.28, 41.57)	0.29 (0.04, 1.78)	0.97 (0.15, 5.48)	1.37 (0.08, 68.08)	0.52 (0.04, 8.78)	0.4 (0.03, 6.17)
0.48 (0.19, 1.28)	1.12 (0.27, 4.95)	0.53 (0.02, 18)	0.52 (0.26, 1)	0.71 (0.12, 4.95)	Ipi_CT	0.31 (0.08, 1.19)	**0.16 (0.03, 0.69)**	2.32 (0.4, 19.01)	**0.21 (0.08, 0.52)**	0.7 (0.33, 1.46)	1.02 (0.09, 38.36)	0.38 (0.05, 3.88)	0.3 (0.03, 2.76)
1.58 (0.41, 6.47)	3.68 (0.64, 20.09)	1.78 (0.05, 66.12)	1.7 (0.51, 5.56)	2.43 (0.28, 21.01)	3.24 (0.84, 12.82)	Niv_CT	0.52 (0.11, 2.06)	7.6 (0.94, 76.92)	0.67 (0.17, 2.57)	2.26 (0.66, 7.9)	3.41 (0.24, 141.52)	1.27 (0.12, 15.61)	0.97 (0.08, 10.81)
3.08 (0.68, 15.03)	**7.01 (1.12, 46.21)**	3.42 (0.08, 135.21)	3.29 (0.87, 14.35)	4.66 (0.51, 45.88)	**6.25 (1.45, 30.49)**	1.94 (0.49, 8.72)	Niv_Ipi	**14.75 (1.58, 156.16)**	1.31 (0.29, 6.3)	**4.43 (1.09, 19.49)**	6.54 (0.42, 296.25)	2.51 (0.22, 32.67)	1.83 (0.15, 23.89)
0.21 (0.02, 1.25)	0.48 (0.04, 3.81)	0.24 (0, 10.14)	0.23 (0.03, 1.14)	0.3 (0.02, 3.54)	0.43 (0.05, 2.52)	0.13 (0.01, 1.06)	**0.07 (0.01, 0.63)**	Niv_Ipi_CT	**0.09 (0.01, 0.52)**	0.3 (0.04, 1.58)	0.45 (0.02, 22.35)	0.16 (0.01, 2.75)	0.12 (0.01, 1.89)
2.32 (0.93, 6.37)	**5.44 (1.29, 22.6)**	2.64 (0.08, 84.87)	**2.52 (1.3, 4.95)**	3.42 (0.56, 24.45)	**4.81 (1.92, 12.82)**	1.49 (0.39, 5.93)	0.76 (0.16, 3.44)	**11.22 (1.92, 94.57)**	Pem	**3.34 (1.79, 6.63)**	4.92 (0.44, 217.43)	1.89 (0.21, 19.5)	1.42 (0.15, 13.12)
0.69 (0.32, 1.52)	1.6 (0.43, 6.22)	0.79 (0.02, 24.52)	0.75 (0.52, 1.05)	1.03 (0.18, 6.5)	1.43 (0.69, 3.05)	0.44 (0.13, 1.52)	**0.23 (0.05, 0.92)**	3.29 (0.63, 25.89)	**0.3 (0.15, 0.56)**	Pem_CT	1.44 (0.14, 55.47)	0.55 (0.07, 5.36)	0.42 (0.05, 3.62)
0.48 (0.01, 5.57)	1.08 (0.02, 15.21)	0.48 (0, 34.23)	0.52 (0.01, 5.24)	0.73 (0.01, 12.56)	0.98 (0.03, 11.07)	0.29 (0.01, 4.25)	0.15 (0, 2.4)	2.25 (0.04, 46.4)	0.2 (0, 2.26)	0.69 (0.02, 7.14)	Sug_CT	0.37 (0.01, 10.33)	0.28 (0, 6.96)
1.28 (0.12, 10.71)	2.9 (0.21, 31.87)	1.38 (0.02, 70.64)	1.35 (0.14, 10.46)	1.93 (0.11, 26.67)	2.61 (0.26, 21.77)	0.79 (0.06, 8.36)	0.4 (0.03, 4.47)	6.06 (0.36, 109.47)	0.53 (0.05, 4.66)	1.81 (0.19, 14.2)	2.69 (0.1, 175.46)	Tis_CT	0.75 (0.03, 13.71)
1.63 (0.18, 16.57)	3.74 (0.32, 46.15)	1.96 (0.03, 103.38)	1.74 (0.21, 15.42)	2.5 (0.16, 38.88)	3.38 (0.36, 32.63)	1.04 (0.09, 12.16)	0.55 (0.04, 6.77)	8.21 (0.53, 148.48)	0.71 (0.08, 6.68)	2.35 (0.28, 21.26)	3.56 (0.14, 211.16)	1.34 (0.07, 30.91)	Tor_CT

F. Hypothyroidism

Ate_CT	1.16 (0.01, 144.25)	1.05 (0.02, 66.45)	1.06 (0.03, 30.78)	1.07 (0.01, 137.51)	1.19 (0.01, 296.49)	1.16 (0, 404.17)	1.03 (0.01, 150.38)	1.21 (0.02, 51.69)	1.42 (0.03, 43.98)	0.6 (0, 81.31)	0.54 (0, 69.95)	0.99 (0.01, 122.76)
0.86 (0.01, 180.97)	Ave_CT	0.92 (0.02, 43)	0.92 (0.03, 27.42)	0.94 (0, 125.33)	0.99 (0.01, 305.99)	1 (0, 290.25)	0.94 (0.01, 122.53)	1.01 (0.02, 43.25)	1.16 (0.03, 41.04)	0.46 (0, 66.48)	0.42 (0, 53.55)	0.87 (0.01, 98.24)
0.95 (0.02, 56.13)	1.09 (0.02, 59.84)	Cam_CT	1.02 (0.14, 5.91)	1.1 (0.01, 51.53)	1.11 (0.02, 114.1)	1.11 (0.01, 131.83)	0.9 (0.02, 67.95)	1.12 (0.07, 11.66)	1.28 (0.16, 9.69)	0.55 (0.01, 31.75)	0.52 (0.01, 21.78)	0.92 (0.02, 44.32)
0.94 (0.03, 36.19)	1.08 (0.04, 39.51)	0.98 (0.17, 7.34)	CT	1.05 (0.02, 37.33)	1.06 (0.04, 78.77)	1.11 (0.01, 100.23)	0.96 (0.03, 43.42)	1.07 (0.19, 5.39)	1.27 (0.53, 3.18)	0.55 (0.01, 20.39)	0.49 (0.01, 15.32)	0.9 (0.03, 29.87)
0.94 (0.01, 146.42)	1.07 (0.01, 266.29)	0.91 (0.02, 83.3)	0.95 (0.03, 50.37)	Dur_CT	1.06 (0.01, 328.98)	1.08 (0, 287.76)	1 (0.01, 161.08)	1.01 (0.02, 61.38)	1.21 (0.03, 83.22)	0.5 (0, 102.34)	0.46 (0, 85.98)	0.92 (0.01, 124.71)
0.84 (0, 132.68)	1.01 (0, 127.04)	0.9 (0.01, 40.69)	0.95 (0.01, 25.97)	0.94 (0, 123.63)	Niv_CT	1.01 (0.01, 32.9)	0.86 (0, 152.15)	1.01 (0.01, 39.63)	1.2 (0.02, 38.29)	0.45 (0, 77.52)	0.44 (0, 56.88)	0.81 (0, 94.5)
0.86 (0, 248.42)	1 (0, 204.35)	0.9 (0.01, 87.01)	0.9 (0.01, 69.82)	0.93 (0, 209.08)	0.99 (0.03, 81.85)	Niv_Ipi	0.85 (0.01, 215.31)	0.99 (0.01, 99.92)	1.16 (0.01, 84.4)	0.45 (0, 128.15)	0.46 (0, 93.38)	0.83 (0, 169.88)
0.97 (0.01, 176.18)	1.07 (0.01, 190.3)	1.11 (0.01, 53.3)	1.04 (0.02, 38.57)	1 (0.01, 163.53)	1.16 (0.01, 202.92)	1.18 (0, 197.39)	Niv_Ipi_CT	1.11 (0.02, 51.94)	1.3 (0.03, 52.13)	0.53 (0, 88.37)	0.47 (0, 81.71)	0.95 (0.01, 96.56)
0.83 (0.02, 53.74)	0.99 (0.02, 57.89)	0.9 (0.09, 13.43)	0.93 (0.19, 5.25)	0.99 (0.02, 40.72)	0.99 (0.03, 84.66)	1.01 (0.01, 94.26)	0.9 (0.02, 46.17)	Pem	1.2 (0.25, 6.47)	0.5 (0.01, 26.28)	0.45 (0.01, 22.23)	0.89 (0.02, 30.28)
0.7 (0.02, 30.36)	0.86 (0.02, 34.3)	0.78 (0.1, 6.17)	0.79 (0.31, 1.9)	0.82 (0.01, 32.47)	0.83 (0.03, 60.3)	0.86 (0.01, 82.76)	0.77 (0.02, 35.93)	0.84 (0.15, 4.02)	Pem_CT	0.43 (0.01, 18.08)	0.38 (0.01, 13.63)	0.69 (0.02, 25.35)
1.67 (0.01, 444.93)	2.18 (0.02, 273.6)	1.81 (0.03, 145.57)	1.83 (0.05, 103.12)	2 (0.01, 391.61)	2.21 (0.01, 525.56)	2.2 (0.01, 542.48)	1.88 (0.01, 302.57)	1.99 (0.04, 111.05)	2.34 (0.06, 120.43)	Sin_CT	0.9 (0, 130.42)	1.73 (0.01, 226.02)
1.86 (0.01, 549.01)	2.38 (0.02, 378.28)	1.93 (0.05, 171.92)	2.05 (0.07, 93.87)	2.17 (0.01, 460.61)	2.28 (0.02, 965.37)	2.16 (0.01, 829.66)	2.14 (0.01, 403.45)	2.24 (0.04, 122.87)	2.65 (0.07, 126.89)	1.12 (0.01, 251.91)	Sug_CT	1.84 (0.01, 449.65)
1.01 (0.01, 182.7)	1.15 (0.01, 196.65)	1.09 (0.02, 49.26)	1.11 (0.03, 36.33)	1.09 (0.01, 180.74)	1.24 (0.01, 210.75)	1.2 (0.01, 222.63)	1.06 (0.01, 128.62)	1.13 (0.03, 52.78)	1.45 (0.04, 49.41)	0.58 (0, 80.68)	0.54 (0, 67.18)	Tor_CT

G. Nausea

Ate_CT	1.29 (0.28, 6.35)	1.43 (0.41, 5.74)	1.06 (0.46, 2.44)	0.16 (0.01, 1.5)	1.28 (0.35, 4.81)	1.47 (0.38, 6.01)	**0.09 (0, 0.75)**	1.81 (0.3, 12.61)	**0.11 (0.03, 0.4)**	1.14 (0.46, 2.79)	2.55 (0.38, 22.41)	0.13 (0, 1.72)	0.28 (0.01, 4.04)	0.47 (0.04, 3.83)
0.77 (0.16, 3.61)	Ave_CT	1.11 (0.22, 5.91)	0.83 (0.21, 3.03)	0.13 (0, 1.44)	1 (0.18, 5.16)	1.14 (0.19, 6.69)	**0.07 (0, 0.78)**	1.39 (0.17, 12.63)	**0.09 (0.02, 0.43)**	0.89 (0.22, 3.42)	1.98 (0.23, 21.22)	0.1 (0, 1.63)	0.21 (0, 4.03)	0.37 (0.02, 3.69)
0.7 (0.17, 2.46)	0.9 (0.17, 4.58)	Cam_CT	0.73 (0.25, 2.01)	0.12 (0, 1.12)	0.89 (0.19, 3.77)	1.02 (0.21, 4.59)	**0.06 (0, 0.55)**	1.23 (0.19, 9.35)	**0.08 (0.02, 0.34)**	0.79 (0.25, 2.29)	1.74 (0.24, 18.14)	0.09 (0, 1.4)	0.19 (0, 2.91)	0.32 (0.03, 2.85)
0.94 (0.41, 2.17)	1.21 (0.33, 4.77)	1.36 (0.5, 3.99)	CT	0.16 (0.01, 1.21)	1.2 (0.44, 3.39)	1.4 (0.45, 4.29)	**0.09 (0, 0.58)**	1.66 (0.35, 9.87)	**0.1 (0.03, 0.27)**	1.08 (0.75, 1.55)	2.35 (0.46, 17.32)	0.13 (0, 1.42)	0.27 (0.01, 3.34)	0.44 (0.05, 2.9)
6.13 (0.67, 160.98)	7.87 (0.7, 224.76)	8.67 (0.89, 245.28)	6.42 (0.83, 149.32)	Dur_CT	7.92 (0.72, 221.69)	9.21 (0.86, 266.83)	0.55 (0.01, 21.42)	11.31 (0.83, 415.79)	0.68 (0.06, 17.26)	6.98 (0.84, 164.66)	15.62 (0.97, 645.16)	0.86 (0.02, 46.7)	1.85 (0.03, 94.56)	2.84 (0.15, 129.27)
0.78 (0.21, 2.86)	1 (0.19, 5.43)	1.13 (0.27, 5.23)	0.83 (0.3, 2.27)	0.13 (0, 1.38)	Ipi_CT	1.16 (0.26, 5.31)	**0.07 (0, 0.64)**	1.4 (0.22, 10.54)	**0.09 (0.02, 0.37)**	0.91 (0.3, 2.62)	1.94 (0.29, 18.72)	0.11 (0, 1.68)	0.22 (0.01, 3.36)	0.37 (0.03, 2.96)
0.68 (0.17, 2.64)	0.88 (0.15, 5.33)	0.98 (0.22, 4.68)	0.72 (0.23, 2.21)	0.11 (0, 1.16)	0.86 (0.19, 3.87)	Niv_CT	**0.06 (0, 0.41)**	1.21 (0.18, 10.04)	**0.07 (0.02, 0.32)**	0.78 (0.24, 2.45)	1.76 (0.22, 16.33)	0.09 (0, 1.28)	0.19 (0, 3.02)	0.32 (0.03, 2.94)
**10.91 (1.33, 278.55)**	**14.19 (1.29, 436.41)**	**16.39 (1.82, 351.55)**	**11.33 (1.71, 250.9)**	1.82 (0.05, 88.63)	**14.43 (1.57, 310.63)**	**15.94 (2.43, 364.14)**	Niv_Ipi	**21.12 (1.58, 576.36)**	1.24 (0.13, 27.26)	**12.44 (1.82, 256.38)**	**29.99 (2.05, 931.68)**	1.54 (0.03, 56.53)	3.18 (0.05, 191.62)	5.37 (0.31, 188.13)
0.55 (0.08, 3.33)	0.72 (0.08, 5.76)	0.81 (0.11, 5.2)	0.6 (0.1, 2.86)	0.09 (0, 1.21)	0.71 (0.09, 4.58)	0.83 (0.1, 5.58)	**0.05 (0, 0.63)**	Niv_Ipi_CT	**0.06 (0.01, 0.39)**	0.64 (0.1, 3.16)	1.42 (0.11, 17.69)	0.07 (0, 1.64)	0.15 (0, 2.96)	0.26 (0.02, 2.87)
**8.87 (2.49, 36.89)**	**11.58 (2.31, 66.27)**	**13.24 (2.95, 59.06)**	**9.54 (3.68, 29.02)**	1.47 (0.06, 17.55)	**11.58 (2.71, 50.41)**	**13.49 (3.11, 63.67)**	0.81 (0.04, 7.41)	**16.37 (2.55, 133.04)**	Pem	**10.22 (3.95, 31.8)**	**23.43 (3.61, 197.21)**	1.24 (0.03, 16.89)	2.6 (0.06, 38.2)	4.28 (0.4, 35.78)
0.88 (0.36, 2.16)	1.12 (0.29, 4.62)	1.27 (0.44, 4.03)	0.93 (0.65, 1.34)	0.14 (0.01, 1.2)	1.1 (0.38, 3.37)	1.29 (0.41, 4.21)	**0.08 (0, 0.55)**	1.56 (0.32, 9.7)	**0.1 (0.03, 0.25)**	Pem_CT	2.2 (0.4, 17.16)	0.12 (0, 1.36)	0.25 (0.01, 3.2)	0.41 (0.04, 2.73)
0.39 (0.04, 2.64)	0.5 (0.05, 4.41)	0.58 (0.06, 4.14)	0.43 (0.06, 2.17)	0.26 (0, 1.14)	0.52 (0.05, 3.47)	0.57 (0.06, 4.49)	**0.03 (0, 0.49)**	0.71 (0.06, 8.82)	**0.04 (0.01, 0.28)**	0.46 (0.06, 2.48)	Sin_CT	0.05 (0, 1.03)	0.12 (0, 2.41)	0.18 (0.01, 2.28)
7.51 (0.58, 243.76)	9.59 (0.61, 356.52)	11.04 (0.72, 465.9)	7.77 (0.7, 236.06)	1.16 (0.02, 54.72)	9.47 (0.6, 347.84)	11.18 (0.78, 411.04)	0.65 (0.02, 33.29)	14.15 (0.61, 619.07)	0.8 (0.06, 30.85)	8.38 (0.74, 266.73)	18.84 (0.97, 993.13)	Sug_CT	1.96 (0.03, 199.04)	3.5 (0.12, 190.18)
3.56 (0.25, 157.35)	4.7 (0.25, 213.31)	5.27 (0.34, 227.24)	3.73 (0.3, 150.72)	0.54 (0.01, 34.02)	4.58 (0.3, 197.56)	5.27 (0.33, 245.14)	0.31 (0.01, 20.02)	6.78 (0.34, 362.99)	0.39 (0.03, 18.17)	4 (0.31, 163.78)	8.38 (0.42, 645.62)	0.51 (0.01, 36.14)	Tis_CT	1.73 (0.07, 89.92)
2.11 (0.26, 23.52)	2.72 (0.27, 47.12)	3.1 (0.35, 34.81)	2.27 (0.35, 21.65)	0.35 (0.01, 6.79)	2.67 (0.34, 31.08)	3.15 (0.34, 38.01)	0.19 (0.01, 3.27)	3.87 (0.35, 63.86)	0.23 (0.03, 2.51)	2.44 (0.37, 22.87)	5.53 (0.44, 109.11)	0.29 (0.01, 8.42)	0.58 (0.01, 15.33)	Tor_CT

H. Pneumonia

Ate_CT	1.21 (0.33, 4.47)	0.56 (0.22, 1.28)	0.3 (0.06, 1.49)	1.14 (0.39, 3.45)	0.64 (0.04, 19.53)	0.27 (0.05, 1.39)	1.94 (0.39, 9.04)
0.83 (0.22, 3)	Cam_CT	0.47 (0.17, 1.16)	0.25 (0.05, 1.17)	0.96 (0.3, 2.97)	0.54 (0.03, 18.43)	0.23 (0.04, 1.2)	1.6 (0.31, 7.93)
1.77 (0.78, 4.58)	2.11 (0.87, 5.86)	CT	0.53 (0.13, 2.08)	**2.05 (1.11, 4.08)**	1.16 (0.08, 32.28)	0.49 (0.12, 1.97)	3.43 (0.94, 13.68)
3.37 (0.67, 17.46)	3.96 (0.85, 22.07)	1.9 (0.48, 7.61)	Dur_CT	3.92 (0.86, 18.69)	2.14 (0.1, 88.17)	0.91 (0.13, 6.72)	6.56 (1, 46.71)
0.88 (0.29, 2.59)	1.04 (0.34, 3.31)	**0.49 (0.24, 0.9)**	0.25 (0.05, 1.16)	Pem_CT	0.56 (0.03, 18.91)	0.24 (0.05, 1.06)	1.66 (0.38, 7.55)
1.56 (0.05, 27.75)	1.86 (0.05, 33.92)	0.87 (0.03, 13.27)	0.47 (0.01, 9.88)	1.79 (0.05, 30.59)	Sin_CT	0.41 (0.01, 10.28)	2.96 (0.08, 62.01)
3.71 (0.72, 18.87)	4.35 (0.84, 23.89)	2.05 (0.51, 8.42)	1.1 (0.15, 7.59)	4.2 (0.95, 20.03)	2.42 (0.1, 82.4)	Sug_CT	7.02 (1.09, 46.97)
0.51 (0.11, 2.55)	0.62 (0.13, 3.19)	0.29 (0.07, 1.06)	0.15 (0.02, 1.02)	0.6 (0.13, 2.64)	0.34 (0.02, 12.69)	0.14 (0.02, 1.02)	Tor_CT

I. Pruritus

Ave_CT	1.3 (0.02, 72.18)	0.91 (0.02, 37.82)	1.76 (0.03, 85.46)	0.81 (0, 128.33)	4.99 (0.06, 572.15)	4.92 (0.05, 700.85)	0.75 (0.01, 39.34)	1.71 (0.03, 76.01)	0.45 (0, 71)	0.89 (0, 146.89)
0.77 (0.01, 49.78)	Cam_CT	0.73 (0.12, 3.64)	1.37 (0.16, 10.8)	0.62 (0.01, 45.81)	3.76 (0.18, 147.48)	3.82 (0.2, 138.87)	0.57 (0.04, 6.89)	1.35 (0.15, 10.51)	0.35 (0.01, 15.84)	0.66 (0.01, 45.93)
1.1 (0.03, 49.22)	1.38 (0.27, 8.32)	CT	1.89 (0.58, 7.28)	0.89 (0.02, 45.97)	4.91 (0.5, 149.92)	5.08 (0.51, 118.09)	0.81 (0.09, 4.97)	1.85 (0.54, 6.88)	0.5 (0.02, 16.58)	0.93 (0.02, 46.4)
0.57 (0.01, 29.25)	0.73 (0.09, 6.32)	0.53 (0.14, 1.71)	Ipi_CT	0.48 (0.01, 31.65)	2.6 (0.18, 102.24)	2.66 (0.18, 75.41)	0.41 (0.04, 3.72)	0.98 (0.16, 5.45)	0.26 (0.01, 10.3)	0.48 (0.01, 28.74)
1.24 (0.01, 274.16)	1.6 (0.02, 122.87)	1.12 (0.02, 65.45)	2.09 (0.03, 165.59)	Niv_CT	5.43 (0.49, 236.75)	6.4 (0.07, 853.86)	0.9 (0.01, 67.38)	2.15 (0.04, 137.71)	0.52 (0, 120.11)	1.04 (0.01, 205.44)
0.2 (0, 15.86)	0.27 (0.01, 5.56)	0.2 (0.01, 2.02)	0.38 (0.01, 5.57)	0.18 (0, 2.03)	Niv_Ipi	1.04 (0.02, 45.8)	0.15 (0, 3.15)	0.36 (0.01, 5.44)	0.09 (0, 6.24)	0.17 (0, 14.52)
0.2 (0, 19.81)	0.26 (0.01, 5.11)	0.2 (0.01, 1.98)	0.38 (0.01, 5.61)	0.16 (0, 14.84)	0.96 (0.02, 56.83)	Niv_Ipi_CT	0.15 (0, 3.05)	0.35 (0.01, 5.38)	0.09 (0, 6.4)	0.16 (0, 16.28)
1.34 (0.03, 106.39)	1.75 (0.15, 26.74)	1.23 (0.2, 10.76)	2.42 (0.27, 28.43)	1.11 (0.01, 88.84)	6.56 (0.32, 321.07)	6.87 (0.33, 273.57)	Pem	2.32 (0.41, 18.79)	0.61 (0.01, 44.72)	1.2 (0.02, 93.58)
0.58 (0.01, 28.82)	0.74 (0.1, 6.88)	0.54 (0.15, 1.85)	1.02 (0.18, 6.27)	0.46 (0.01, 24.48)	2.77 (0.18, 95.54)	2.83 (0.19, 79.82)	0.43 (0.05, 2.44)	Pem_CT	0.26 (0.01, 10.25)	0.5 (0.01, 27.52)
2.23 (0.01, 370.37)	2.89 (0.06, 141.47)	2.01 (0.06, 65.37)	3.82 (0.1, 162.92)	1.91 (0.01, 228.12)	11.15 (0.16, 993.61)	11.08 (0.16, 961.6)	1.65 (0.02, 82.88)	3.83 (0.1, 157.45)	Sin_CT	1.9 (0.01, 303.55)
1.13 (0.01, 230.22)	1.51 (0.02, 81.64)	1.08 (0.02, 42.43)	2.07 (0.03, 96.24)	0.96 (0, 142.09)	5.82 (0.07, 653.24)	6.16 (0.06, 585.5)	0.83 (0.01, 43.86)	1.98 (0.04, 90.13)	0.53 (0, 78.77)	Tor_CT

J. Pyrexia

Ate_CT	0.93 (0.01, 95.26)	0.33 (0, 51.54)	0.38 (0.01, 6.53)	0.84 (0.01, 73.08)	0.24 (0, 7.8)	0.97 (0.02, 23.44)	0.63 (0.01, 22.62)	0.38 (0, 36.46)	0.9 (0.01, 79.44)
1.08 (0.01, 95.81)	Ave_CT	0.37 (0, 49.04)	0.42 (0.01, 7.12)	0.91 (0.01, 76.35)	0.26 (0, 8.53)	1.07 (0.02, 26.42)	0.67 (0.01, 26.26)	0.4 (0, 46.12)	0.98 (0.01, 94.8)
3.01 (0.02, 465.96)	2.68 (0.02, 521.07)	Cam_CT	1.09 (0.02, 44.92)	2.4 (0.02, 427.85)	0.73 (0.01, 47.89)	2.84 (0.04, 155.56)	1.83 (0.02, 132.55)	1.1 (0, 220.54)	2.56 (0.02, 531.68)
2.61 (0.15, 87.77)	2.39 (0.14, 98.35)	0.92 (0.02, 59.84)	CT	2.16 (0.12, 77.95)	0.67 (0.07, 4.68)	2.59 (0.68, 11.47)	1.65 (0.21, 16.03)	1.02 (0.02, 44.23)	2.28 (0.14, 92.58)
1.18 (0.01, 119.15)	1.1 (0.01, 122.36)	0.42 (0, 59.8)	0.46 (0.01, 8.14)	Ipi_CT	0.28 (0.01, 9.11)	1.19 (0.03, 28.79)	0.75 (0.01, 29.59)	0.44 (0, 59.97)	1.05 (0.01, 117.97)
4.24 (0.13, 250.3)	3.81 (0.12, 250.76)	1.37 (0.02, 165.95)	1.5 (0.21, 14.36)	3.53 (0.11, 186.66)	Pem	3.88 (0.65, 32.06)	2.55 (0.14, 59.52)	1.54 (0.02, 107.87)	3.64 (0.12, 238.15)
1.03 (0.04, 46.23)	0.93 (0.04, 46.28)	0.35 (0.01, 28.12)	0.39 (0.09, 1.48)	0.84 (0.03, 34.91)	0.26 (0.03, 1.55)	Pem_CT	0.65 (0.05, 9.31)	0.39 (0.01, 19.86)	0.89 (0.04, 45.02)
1.59 (0.04, 91.48)	1.48 (0.04, 93.49)	0.55 (0.01, 61.64)	0.61 (0.06, 4.73)	1.33 (0.03, 78.19)	0.39 (0.02, 6.91)	1.54 (0.11, 18.9)	Sin_CT	0.62 (0.01, 38.84)	1.42 (0.04, 92.96)
2.66 (0.03, 429.97)	2.52 (0.02, 386.04)	0.91 (0, 249.63)	0.98 (0.02, 42.32)	2.26 (0.02, 351.27)	0.65 (0.01, 42.72)	2.54 (0.05, 141.23)	1.61 (0.03, 124.19)	Tis_CT	2.47 (0.02, 414.41)
1.11 (0.01, 84.26)	1.02 (0.01, 103.69)	0.39 (0, 54.74)	0.44 (0.01, 7.12)	0.95 (0.01, 83.45)	0.27 (0, 8.46)	1.12 (0.02, 28.15)	0.7 (0.01, 26.96)	0.4 (0, 48.84)	Tor_CT

K. Rash

Ate_CT	2.67 (0.14, 67.64)	1.12 (0.11, 13.33)	0.73 (0.15, 3.7)	4.06 (0.5, 37.88)	0.72 (0.01, 56.72)	7.34 (0.28, 388.64)	6.51 (0.24, 439.4)	0.25 (0.03, 2.66)	0.74 (0.12, 5.18)	0.31 (0.02, 3.83)	0.92 (0.02, 61.39)	1.52 (0.1, 28.37)	1.25 (0.07, 25.72)
0.37 (0.01, 7.36)	Ave_CT	0.42 (0.02, 9.77)	0.28 (0.02, 3.41)	1.51 (0.07, 28.88)	0.26 (0, 31.95)	2.83 (0.05, 225.82)	2.47 (0.04, 247.25)	0.09 (0, 2.03)	0.28 (0.01, 4.38)	0.12 (0, 2.73)	0.34 (0, 33.4)	0.56 (0.02, 19.84)	0.47 (0.01, 17.26)
0.89 (0.08, 9.51)	2.38 (0.1, 64.03)	Cam_CT	0.65 (0.1, 3.75)	3.61 (0.35, 36.98)	0.63 (0.01, 55.6)	6.49 (0.22, 377.87)	5.79 (0.18, 395.1)	0.22 (0.02, 2.54)	0.66 (0.08, 5.24)	0.27 (0.02, 3.83)	0.8 (0.02, 60.12)	1.33 (0.07, 27.91)	1.11 (0.05, 24.98)
1.38 (0.27, 6.82)	3.61 (0.29, 60.67)	1.54 (0.27, 10.14)	CT	**5.53 (1.4, 25.33)**	0.99 (0.02, 58.77)	9.93 (0.59, 378.94)	8.68 (0.5, 442.81)	0.35 (0.06, 1.79)	1.01 (0.39, 2.94)	0.43 (0.05, 2.87)	1.24 (0.05, 61.58)	2.07 (0.22, 24.48)	1.71 (0.15, 22.14)
0.25 (0.03, 2.02)	0.66 (0.03, 14.86)	0.28 (0.03, 2.82)	**0.18 (0.04, 0.71)**	Ipi_CT	0.17 (0, 13.15)	1.82 (0.07, 87.55)	1.59 (0.06, 96.56)	**0.06 (0.01, 0.54)**	0.18 (0.03, 1.04)	0.08 (0.01, 0.81)	0.22 (0.01, 13.26)	0.37 (0.02, 6.21)	0.31 (0.02, 5.64)
1.4 (0.02, 103.64)	3.79 (0.03, 481.81)	1.58 (0.02, 129.66)	1.01 (0.02, 56.36)	5.76 (0.08, 393.44)	Niv_CT	9.94 (0.58, 414.94)	9.54 (0.06, 2066.71)	0.35 (0, 27.53)	1.03 (0.02, 66.24)	0.43 (0, 36.92)	1.33 (0.01, 320.3)	2.14 (0.02, 211.78)	1.73 (0.02, 207.71)
0.14 (0, 3.59)	0.35 (0, 20.4)	0.15 (0, 4.57)	0.1 (0, 1.7)	0.55 (0.01, 13.72)	0.1 (0, 1.72)	Niv_Ipi	0.88 (0.01, 106.36)	0.03 (0, 0.98)	0.1 (0, 2.15)	0.04 (0, 1.32)	0.12 (0, 15.65)	0.21 (0, 9)	0.17 (0, 8.03)
0.15 (0, 4.13)	0.4 (0, 23.27)	0.17 (0, 5.56)	0.12 (0, 1.99)	0.63 (0.01, 15.89)	0.1 (0, 15.6)	1.14 (0.01, 113.84)	Niv_Ipi_CT	0.04 (0, 1.15)	0.12 (0, 2.49)	0.05 (0, 1.5)	0.14 (0, 17.87)	0.23 (0, 10.57)	0.19 (0, 9.3)
4.06 (0.38, 39.56)	10.77 (0.49, 272.23)	4.58 (0.39, 52.53)	2.9 (0.56, 15.53)	**16.4 (1.86, 149.37)**	2.87 (0.04, 221.79)	29.71 (1.03, 1555.48)	26.26 (0.87, 1740.22)	Pem	2.97 (0.58, 16.28)	1.27 (0.08, 15.47)	3.74 (0.09, 244.4)	6.15 (0.35, 115.7)	5.15 (0.25, 99.47)
1.36 (0.19, 8.42)	3.58 (0.23, 68.88)	1.53 (0.19, 12.24)	0.99 (0.34, 2.55)	5.49 (0.96, 32.24)	0.97 (0.02, 61.66)	9.83 (0.46, 410.36)	8.54 (0.4, 484.95)	0.34 (0.06, 1.72)	Pem_CT	0.42 (0.04, 3.52)	1.23 (0.04, 67.72)	2.04 (0.17, 28.37)	1.69 (0.11, 25.09)
3.19 (0.26, 44.08)	8.67 (0.37, 274.51)	3.66 (0.26, 57.94)	2.3 (0.35, 18.44)	13.01 (0.98, 168.08)	2.32 (0.03, 224.46)	24.09 (0.76, 1558.66)	21.38 (0.67, 1594.81)	0.79 (0.06, 12.01)	2.37 (0.28, 24.75)	Sin_CT	2.98 (0.06, 241.51)	4.91 (0.25, 120.37)	4.04 (0.18, 108.08)
1.09 (0.02, 42.49)	2.98 (0.03, 208.49)	1.24 (0.02, 53.45)	0.8 (0.02, 21.4)	4.48 (0.08, 167.88)	0.75 (0, 149.57)	8.05 (0.06, 1109.1)	7.3 (0.06, 1157.88)	0.27 (0, 11.73)	0.81 (0.01, 25.99)	0.34 (0, 15.4)	Sug_CT	1.65 (0.02, 100)	1.35 (0.01, 87.28)
0.66 (0.04, 10.51)	1.77 (0.05, 63.32)	0.75 (0.04, 14.26)	0.48 (0.04, 4.57)	2.7 (0.16, 42.41)	0.47 (0, 46.59)	4.87 (0.11, 340.44)	4.32 (0.09, 372.9)	0.16 (0.01, 2.84)	0.49 (0.04, 6.01)	0.2 (0.01, 3.93)	0.61 (0.01, 57.03)	Tis_CT	0.81 (0.03, 25.12)
0.8 (0.04, 14.88)	2.13 (0.06, 87.41)	0.9 (0.04, 19.94)	0.58 (0.05, 6.73)	3.25 (0.18, 58.12)	0.58 (0, 66.54)	5.97 (0.12, 461.42)	5.23 (0.11, 501.26)	0.19 (0.01, 4.07)	0.59 (0.04, 8.84)	0.25 (0.01, 5.5)	0.74 (0.01, 71.12)	1.23 (0.04, 38.47)	Tor_CT

eFigure 8. Network meta-analysis sensitivity analysis

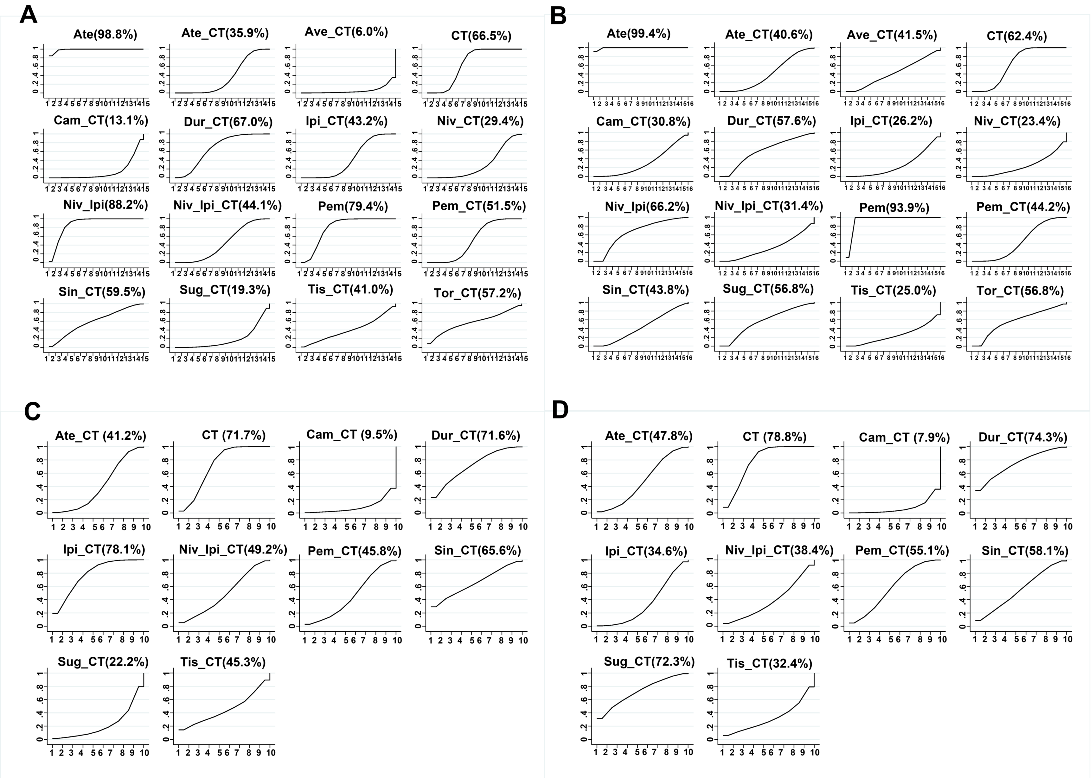

A-B, Only phase III clinical studies were included in this network meta-analysis; C-D, Only lung cancer patients were included in the network meta-analysis.

eFigure 9. Trace plot for the evaluation of model convergence

A. Any grade

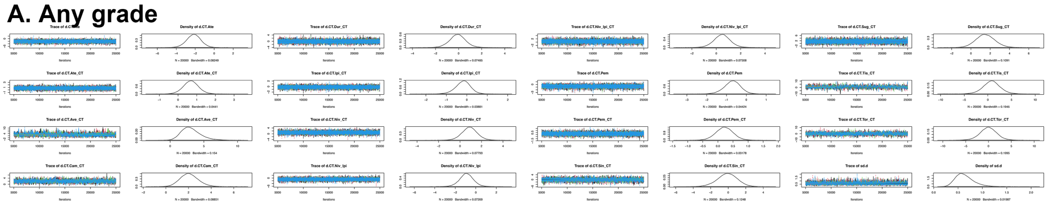

B. Grade 3 and above

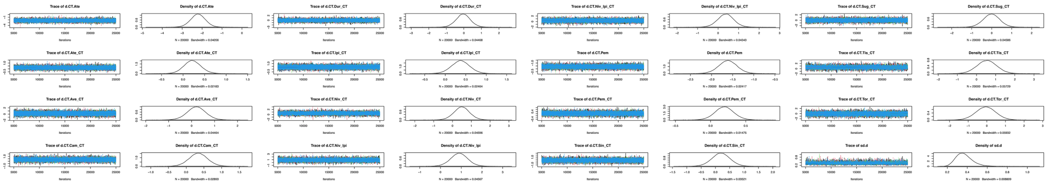

C. ALT elevation

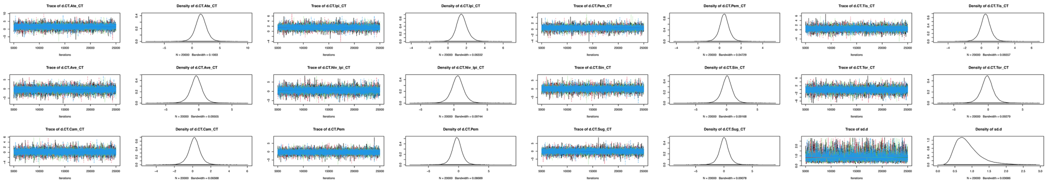

D. AST elevation

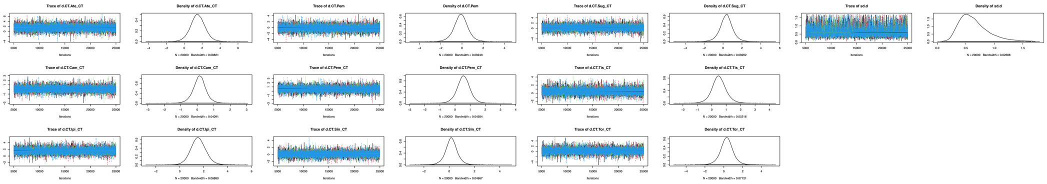

E. Decreased appetite

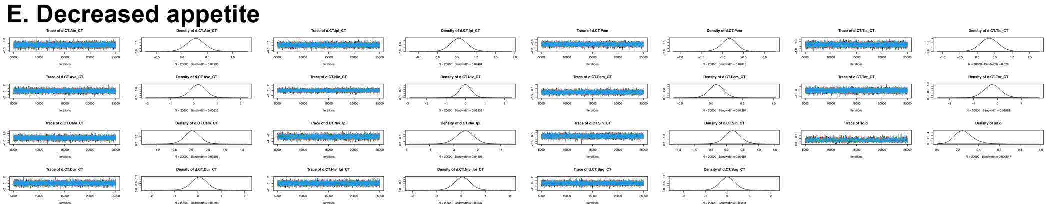

F. Diarrhea

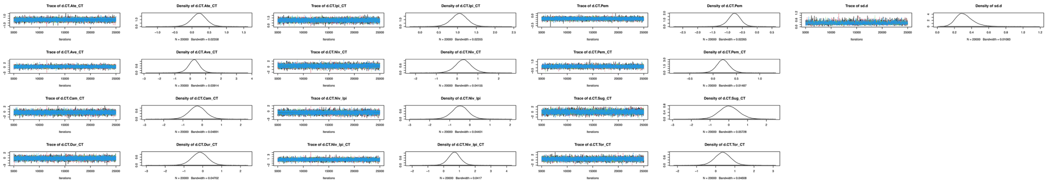

G. Nausea

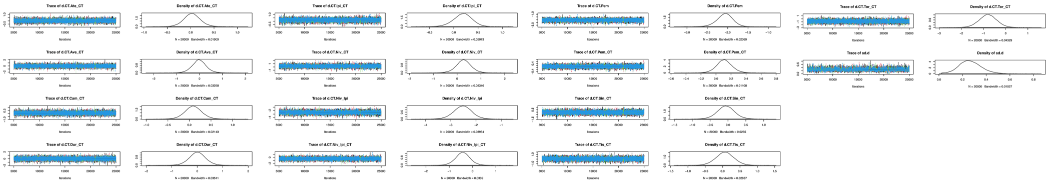

H. Fatigue

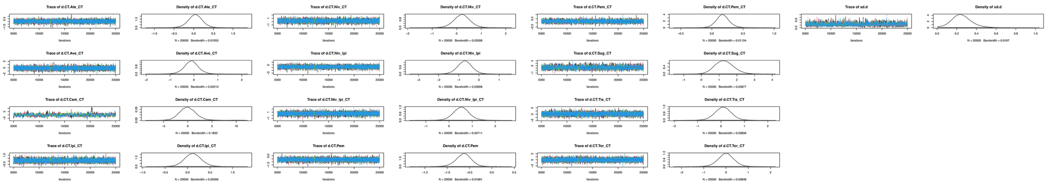

I. Pneumonia

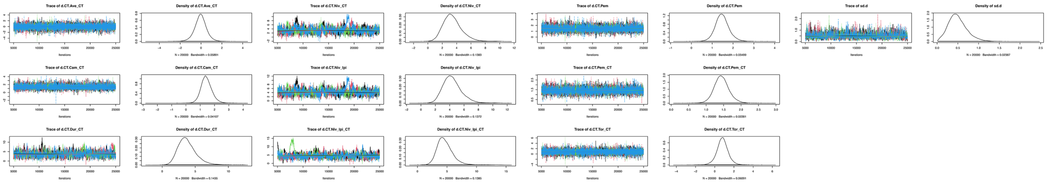

J. Hypothyroidism

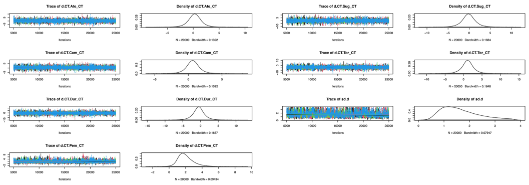

K. Pruritus

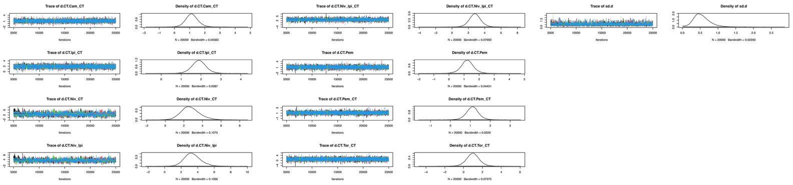

L. Rash

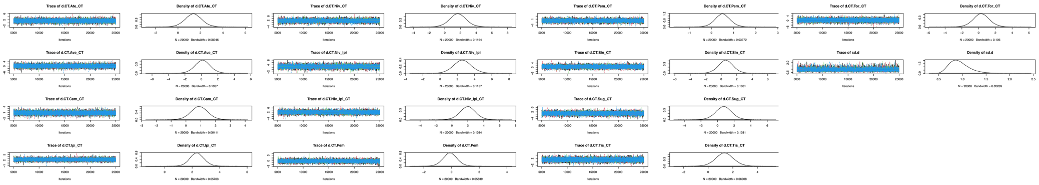

M. Pyrexia

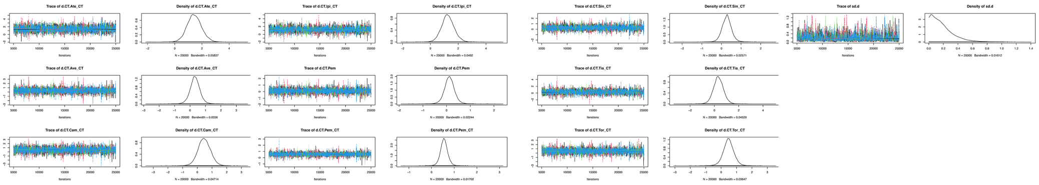

Overlapping MCMC chain area accounts for most of the chain fluctuation range, the fluctuation of an individual chain was not apparent, and density map distribution pattern was normal, consistent with satisfactory model convergence.

eFigure 10 Brooks-Gelman-Rubin diagnostic plots for the evaluation of model convergence

A. Any grade

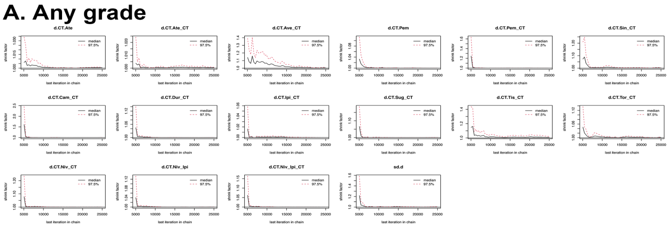

B. Grade 3 and above

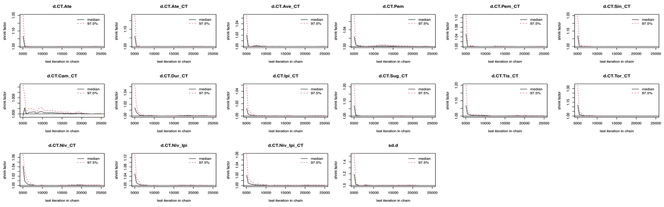

C. ALT elevation

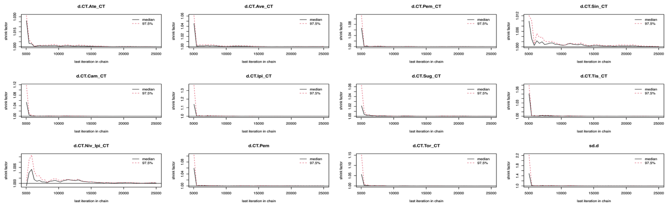

D. AST elevation

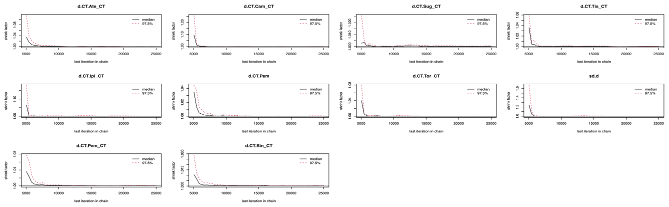

E. Decreased appetite

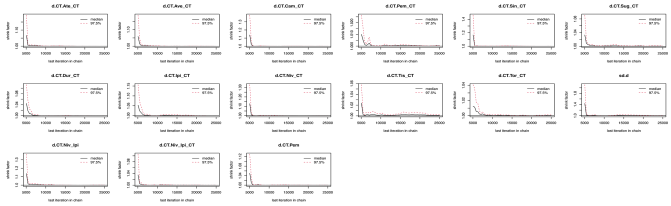

F. Diarrhea

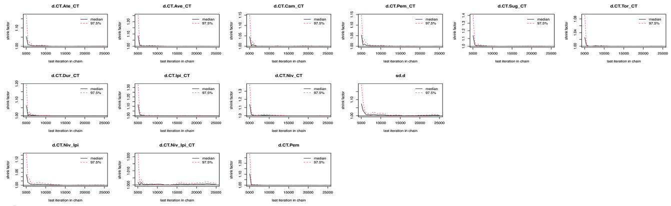

G. Nausea

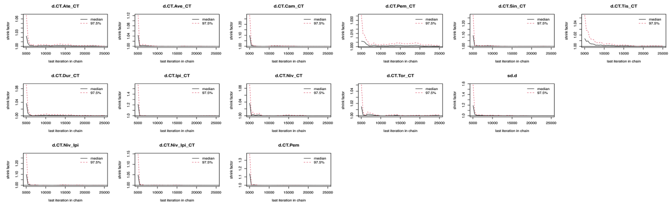

H. Fatigue

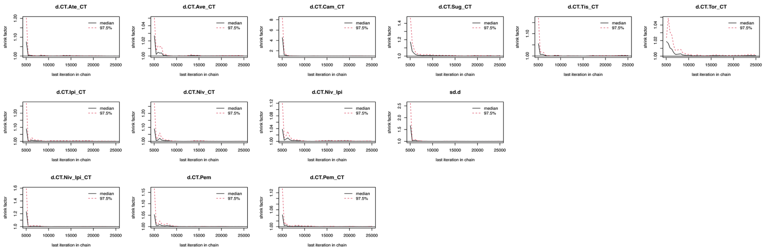

I. Pneumonia

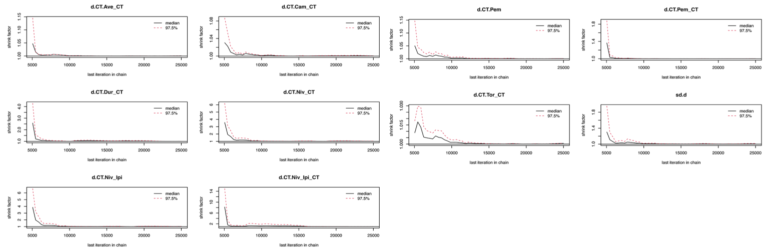

J. Hypothyroidism

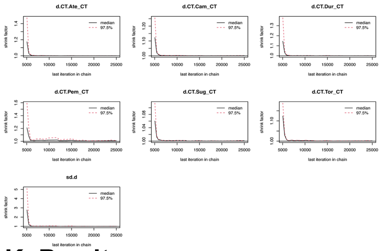

K. Pruritus

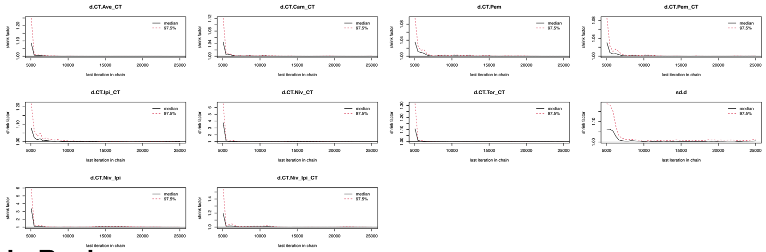

L. Rash

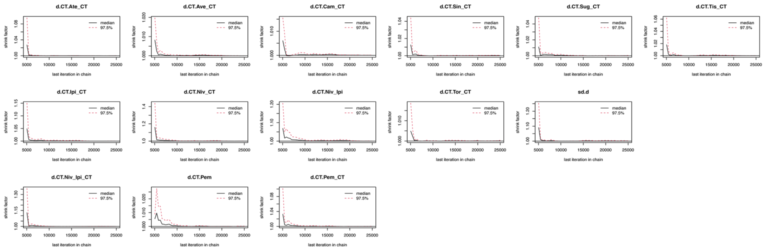

M. Pyrexia

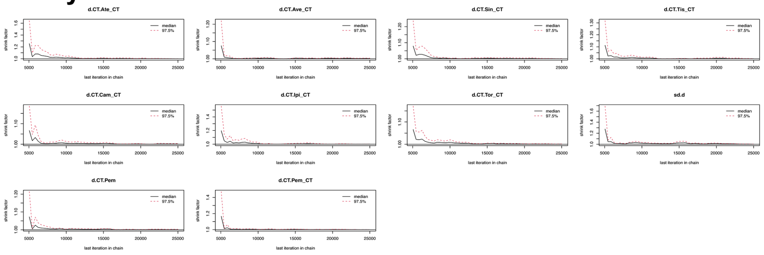

The median shrink factor and its 97.5% value rapidly converge to 1 and stabilize, consistent with a satisfactory level of model convergence.
